# A comparison of three strategies to reduce the burden of osteoarthritis: A population-based microsimulation study

**DOI:** 10.1371/journal.pone.0261017

**Published:** 2021-12-08

**Authors:** Jacek A. Kopec, Eric C. Sayre, Anya Okhmatovskaia, Jolanda Cibere, Linda C. Li, Nick Bansback, Hubert Wong, Shahzad Ghanbarian, John M. Esdaile

**Affiliations:** 1 University of British Columbia, Vancouver, British Columbia, Canada; 2 Arthritis Research Canada, Richmond, British Columbia, Canada; 3 McGill University, Montreal, Québec, Canada; 4 Centre of Clinical Epidemiology and Evaluation, Vancouver, British Columbia, Canada; Icahn School of Medicine at Mount Sinai, UNITED STATES

## Abstract

**Objectives:**

The purpose of this study was to compare three strategies for reducing population health burden of osteoarthritis (OA): improved pharmacological treatment of OA-related pain, improved access to joint replacement surgery, and prevention of OA by reducing obesity and overweight.

**Methods:**

We applied a validated computer microsimulation model of OA in Canada. The model simulated a Canadian-representative open population aged 20 years and older. Variables in the model included demographics, body mass index, OA diagnosis, OA treatment, mortality, and health-related quality of life. Model parameters were derived from analyses of national surveys, population-based administrative data, a hospital-based cohort study, and the literature. We compared 8 what-if intervention scenarios in terms of disability-adjusted life years (DALYs) relative to base-case, over a wide range of time horizons.

**Results:**

Reductions in DALYs depended on the type of intervention, magnitude of the intervention, and the time horizon. Medical interventions (a targeted increase in the use of painkillers) tended to produce effects quickly and were, therefore, most effective over a short time horizon (a decade). Surgical interventions (increased access to joint replacement) were most effective over a medium time horizon (two decades or longer). Preventive interventions required a substantial change in BMI to generate a significant impact, but produced more reduction in DALYs than treatment strategies over a very long time horizon (several decades).

**Conclusions:**

In this population-based modeling study we assessed the potential impact of three different burden reduction strategies in OA. Data generated by our model may help inform the implementation of strategies to reduce the burden of OA in Canada and elsewhere.

## Introduction

Osteoarthritis (OA) is the most common joint disease and a massive public health problem in Canada and around the world [1–3]. In the US and Canada, about 10–15% of all adults have clinical OA [1, 2]. OA is one of the top reasons for seeing a doctor [4]. According to the Global Burden of Disease (GBD) study, prevalence of OA in high-income countries has increased by 55% since 1990 [5]. The population health burden of OA is expected to continue to rise due to population aging and increasing levels of obesity [2, 3].

Over the past two decades, a number of strategies to halt the OA pandemic have been identified [6–8]. A report sponsored by several European organizations recommended a wide range of strategies, from primary prevention (avoiding obesity, gain in physical fitness, injury prevention) to case finding, better management of pain, biomechanical devices, exercise, and timely access to surgery [6]. Similar approaches have been advocated by patient groups in the US [7] and Canada [8]. Evidence for the effectiveness of pharmacological interventions, non-pharmacological therapies (physical, psychosocial, and mind-body approaches) and surgical treatments have been synthesized in practice guidelines issued by professional organizations, such as the European League Against Rheumatism [9], Osteoarthritis Research International [10], American College of Rheumatology [11], or American College of Orthopaedic Surgeons [12, 13].

To identify and implement the most effective burden-reducing strategies in OA, policymakers need projections of their future population-level health and quality-of-life impact. This information is currently lacking. Recommendations based on individual-level risk and treatment effectiveness data from clinical and epidemiological research are critically important. However, they may not automatically translate into burden reductions at the population level. For example, adherence to guidelines is often far from perfect, resulting in smaller than expected uptake of recommended treatments or preventive behaviors [14, 15]. Furthermore, projecting the impact of interventions or policies of known efficacy on disease burden in an open population is not a trivial task. It may require that several additional factors are taken into consideration, for example, population aging, current treatment and risk factor distribution, frequency of adverse events, and their impact on quality of life. The purpose of this study was to compare three strategies for reducing OA burden at the population level, namely improved pharmacological treatment of OA-related pain, improved access to joint replacement surgery (JRS), and prevention of OA by reducing obesity.

## Methods

### Microsimulation model

We used a previously validated microsimulation model of OA (MSM-OA) [16]. The model generates individual life and health histories by assigning events to individuals over time (continuously or discretely) based on statistical prediction equations. The structure of MSM-OA has been described in previous studies [16, 17]. MSM-OA uses the Population Health Model (POHEM) simulation platform developed at Statistics Canada [18]. It is a model of a Canadian-representative open population 20 years of age and older. As the simulation progresses, individuals age and can be removed from the population by death or emigration. People can also enter the population by becoming 20 years of age or immigrating to it. Key variables in MSM-OA are sociodemographic variables, presence of OA, body mass index (BMI), medication use and side effects, JRS, and health-related quality of life (HRQOL). Model parameters, along with their methods of derivation and sources of data are provided in Appendix 1 in S1 File.

The initial population has been derived from the 2001 cycle of the Canadian Community Health Survey (CCHS) [19]. The CCHS is an ongoing cross-sectional survey of about 130,000 Canadians per two-year cycle. Projected changes in the demographic structure of the population are based on Statistics Canada’s mortality and other demographic projections [20]. BMI has been modeled using self-reported data from the 1994–2006 National Population Health Survey (NPHS), a nationally representative cohort of over 17,000 individuals who were surveyed every 2 years [21]. Log-transformed BMI is predicted from an autoregressive linear model. Predictor variables include prior values of BMI, age, education, income and region. Separate models were developed for males and females.

OA incidence in MSM-OA is a function of age, sex and BMI. Baseline incidence of OA was obtained as the age/sex-specific incidence of physician-diagnosed OA of any joint observed in a comprehensive administrative (claims) database in the province of British Columbia (BC), Canada [22], and applied to the Canadian population age/sex structure. In administrative data, OA was defined as at least two visits to a health professional within two years or one hospital separation with the ICD-9 code 715 or the ICD-10 code M15-M19 [23]. We modeled overall OA since no data on joint-specific OA or multiple joint involvement were available. The initial age/sex-specific prevalence of OA was calibrated such that the population was in a steady state at the beginning of the simulation. Age-adjusted relative risks for the effect of BMI on OA incidence, separately for males and females, have been derived by analyzing multiple cycles of the NPHS in a survival regression model [16].

HRQOL was measured with a well-established multi-attribute measure of health utility, the Health Utilities Index Mark 3 (HUI3) [24]. The attributes (domains or components) are vision, hearing, speech, mobility, dexterity, emotion, cognition, and pain. They were initially measured on a 5 or 6-point ordinal scale; however, all attributes except pain were collapsed to various extent prior to implementation due to reporting limitations of public-use CCHS and small numbers of respondents in some categories, leading to model instability. For pain, the levels were as follows: 1—no pain or discomfort; 2—mild to moderate pain that prevented no activities; 3—moderate pain that prevented a few activities; 4—moderate to severe pain that prevented some activities; and 5—severe pain that prevented most activities. The HUI3 prediction model has been developed from the 1994–2012 cycles of the NPHS. We have modeled each of the 8 HUI3 attributes individually using ordinal logistic regression with proportional odds. Predictors included previous values of the attribute (2 years back), calendar time, demographics (age, sex, education, and income), smoking, BMI, OA, and selected comorbidities (diabetes, hypertension, cardiovascular disease, stroke, ulcer, and non-ulcer dyspepsia), some of which may be affected by OA medication. Additional predictors included other concurrent HUI3 attributes according to a conceptual, hierarchical model. The attributes were updated one at a time, each model including concurrent values of the already-updated attributes.

The frequency of use of four types of pain medication in OA (acetaminophen, NSAIDs, COX-2 inhibitors, and opioids) has been derived from two sources, the PharmaNet database in BC and self-reports of drug use in Canada from the 1996–2012 cycles of the NPHS. PharmaNet includes virtually all prescription drugs dispensed by pharmacies in BC [25]. Self-report data were necessary to account for over-the-counter use, which is common with acetaminophen and NSAIDs. We estimated the probability of medication use in Canada by modeling the ratio of all use to prescription use for acetaminophen and NSAIDs in BC. We estimated the use (point prevalence) of each medication according to age, sex, OA stage (diagnosis of OA<5 years prior, OA≥5 years, post-JRS<5 years, and post-JRS≥5 years), and level of pain as measured by HUI3. We included five types of side effects of pain medication: serious gastrointestinal (GI) complications (ulcer with bleeding or perforation), cardiovascular disease, stroke, dyspepsia, and lethal opioid overdose. Probabilities of developing these side effects were obtained from the literature [26–36]. For GI complications, we applied excess rates among medication users. For CVD and stroke, we obtained baseline incidence for Canada from the GBD study [5] and applied the relative risk among users. For prescription opioid overdose, we obtained the number of deaths in Canada from published reports and calibrated the probabilities by titration [35, 36].

We used administrative data from BC to derive baseline joint (knee or hip) replacement surgery rates [22]. Rates of primary and revision JRS were obtained using a Poisson regression model with three predictors, age, sex, and calendar year. The odds of getting JRS in people with OA according to the level of each HUI3 attribute were derived from a population-based case-control study in which cases were 220 patients with OA scheduled to undergo hip/knee replacement at the Vancouver General Hospital between 2000 and 2005 and controls were age- and sex-matched (3:1) participants in the 2001 CCHS who reported being diagnosed with OA [37, 38].

In our model, medical (pharmacological) treatments of OA could have a positive direct effect on the level of pain in HUI3. Other HUI3 domains could be affected indirectly, because of their relationship with pain. For each class of drugs, their impact on pain, which incorporated placebo effect and was expressed as change in pain relative to baseline, was obtained from an extensive review of the literature [39, 40]. In addition, all HUI3 domains could be negatively affected by the side effects of medical treatment. In particular, certain classes of drugs increase the risk of CVD and stroke and can cause ulcer and non-ulcer dyspepsia. Model coefficients for CVD, stroke, and ulcer were derived from the 2001 cycle of the CCHS and the coefficient for dyspepsia was obtained from the literature [41, 42]. The impact of JRS on HUI3 domains was modeled using data from a cohort of patients treated at the Vancouver General Hospital [37]. Each side effect of medical treatment (except dyspepsia) and JRS were associated with a specified risk of death based on data from the literature [5, 35, 36, 43–47].

### Selection of intervention (counterfactual) scenarios

In a preliminary analysis, we compared a number of intervention scenarios within each treatment strategy in order to select the scenarios for the between-strategy comparisons. This step was necessary to reduce the scenarios to a manageable number and avoid redundancy. For the medical treatment strategy, our goal was to identify target groups in which the benefits of pain reduction outweighed the negative side effects. Thus our question was how much can OA burden be reduced by an appropriately targeted intervention, rather than indiscriminate use of medication. For the surgical treatment strategy, the same principle was applied. Given the limitations on surgical capacity, we additionally considered the net benefits per number of additional surgeries. For the prevention intervention strategy, because everyone who did not have OA was at risk of developing OA, the target group was everyone in the population who was obese or overweight.

For each of the four medication types, we developed scenarios that differed in the odds multiplier (amount by which the odds of using a given drug were increased or decreased) and the intervention target group defined by pain level (2 to 5). We did not include scenarios for patients without pain or discomfort (level 1) as they would not benefit from treatment in our model. Scenarios were compared in terms of differences relative to base-case in average quality-adjusted life years (QALYs) per person in the population, for age groups 20–69 and 70+ years. According to our model, side-effects of treatment outweighed the benefits of pain reduction is some scenarios. Specifically, increased use of traditional NSAIDs and coxibs in OA patients aged 70+ years with mild (level 2) pain reduced population QALYs, and opioids reduced QALYs in all patients with mild pain (Table A2-1, Appendix 2 in S1 File). These results were used to select the final scenarios in which the intervention was applied to those who would, on average, benefit from it.

We developed two scenarios with different multipliers to allow for extrapolation and interpolation of results. In selecting the multipliers, we aimed to generate robust results that are clearly distinguishable from the random noise in the model. In addition, we sought to strike a balance between what is realistic and what is achievable under optimal conditions. Although maximum improvement in population QALYs would ensue if all patients experiencing net benefit (and none experiencing net harm) received medication, we considered such a scenario as too implausible. Given these considerations, we selected the odds multipliers 2 and 3. These scenarios are referred to as Medication x2 and Medication x3.

Within the surgical treatment strategy, we compared scenarios with different JRS rate multipliers and target groups, defined by age (20–49, 50–69 and 70+ years) and pain level (2 to 5). The main outcome was the difference in average QALYs per person in the population compared with base-case. In addition, we compared the scenarios in terms of effectiveness ratio, defined as a ratio of the difference in average QALYs to the difference in the number of JRSs, relative to base-case. As expected, our model predicted that all categories of OA patients with pain ≥2 would, on average, benefit from an increased access to JRS, although the gain in QALYs varied by age, pain level, and rate multiplier (Appendix 2, Table A2-2 in S1 File). In selecting the scenarios for inter-strategy comparisons we applied similar principles to those used for medical scenarios. In addition, we wanted the results to be easily comparable across different strategies. Based on these considerations and the results from the model, we selected two scenarios, in which the rates of JRS were doubled or tripled in all age and pain ≥2 groups. Applying a higher rate multiplier would generally produce more gain in QALYs at the population level, but extremely high rates of JRS (e.g., a multiplier of 20) would be unrealistic and had a lower effectiveness ratio. The selected scenarios are referred to as Surgery x2 and Surgery x3.

For the prevention strategy, we selected four intervention scenarios. The scenarios were selected based on the results of a previously published study of the effect of BMI reduction on OA prevalence [48]. We sought to cover a broad range of trajectories in obesity prevalence, from a stable trend to a strongly declining one. Therefore, we assumed a reduction in BMI ranging from 0.1 to 1.0 units per year in each person with BMI>25. The intervention would last until the individual reached normal weight (BMI<25). The scenarios are referred to as BMI-0.1, BMI-0.3, BMI-0.5 and BMI-1.0. All intervention scenarios and the base-case scenario are described in Table 1.

**Table 1:** Description of the scenarios for inter-strategy comparisons.

Scenario name	Description of intervention
Base-case	No intervention. All variables in the simulation change over time according to underlying statistical prediction models
Medication x2	The odds of taking each of the four classes of medication are multiplied by 2 in the following groups of simulated individuals: Acetaminophen: in all persons with OA and pain≥2; NSAIDs: in all persons with OA aged 20–69 and pain ≥2 and all persons aged 70+ and pain ≥3 Coxibs: in all persons with OA aged 20–69 and pain ≥2 and all persons aged 70+ and pain ≥3 Opioids: in all persons with OA and pain ≥3
Medication x3	The odds of taking medication are multiplied by 3 in the same groups as above
Surgery x2	The hazard rate of obtaining JRS (a parameter in MSM-OA), is multiplied by 2 in all persons with OA and pain ≥2
Surgery x3	The hazard rate of obtaining JRS is multiplied by 3 in all persons with pain ≥2
BMI-0.1	BMI is reduced by 0.1 units per year in all persons with BMI ≥25 until they reach BMI<25
BMI-0.3	BMI is reduced by 0.3 units per year in the same groups as above
BMI-0.5	BMI is reduced by 0.5 units per year in the same groups as above
BMI-1.0	BMI is reduced by 1 unit per year in the same groups as above

OA = Osteoarthritis; BMI = Body mass index; NSAIDs = Non-steroidal anti-inflammatory drugs; MSM = microsimulation model; JRS = Joint replacement surgery

In the medical intervention scenarios, all 4 types of drugs were allowed to be used in the same patient, assuming independent probabilities. The drugs were assumed to act independently in terms of both pain reduction and side effects. Incorporating interactions between drugs was not feasible due to a lack of data and additional model complexity this would entail. In the surgical scenarios, each subject could have up to 4 primary JRSs and any number of revision JRSs. In all counterfactual scenarios, interventions were assumed to be effective starting in 2020. Although actual implementation of medical and surgical interventions would likely stretch over a longer period, modeling details of the implementation process was beyond the scope of our study.

### Sensitivity analysis

We estimated the impact of uncertainty in 17 key model parameters on average lifetime QALYs per person in a one-way sensitivity analysis. For each parameter, we ran the model for the base-case and three intervention scenarios (one per strategy), assuming three values for the parameter, the mean, upper limit, and lower limit of the estimated 95% confidence interval (Appendix 3 in S1 File). We specified the sample size as 1 million for the duration of the simulation, which resulted in about 360,000 simulated individuals in 2020.

### Outcomes

The simulated population was described according to age, sex, BMI distribution, and OA incidence and prevalence. For those with OA, the key variables were medication use, hip/knee replacement surgery rates, side effects of treatment (including mortality) and HRQOL measured by HUI3. Intervention scenarios were compared in terms of disability-adjusted life years (DALYs) due to OA relative to base-case (DALYs averted) over time. We used OA-related DALYs rather than QALYs to compare OA burden associated with treatment and prevention strategies because reducing BMI in the population affects HRQOL and mortality through various mechanisms, some of which are not related to OA. While it might be important from a policymaker’s point of view to estimate all effects associated with an intervention, our goal was more circumscribed—to assess reduction in OA-related burden—and our model did not include other effects of high BMI in the general population. BMI interventions reduced OA-related DALYs by decreasing OA incidence, while medical and surgical interventions improved HRQOL in people with OA. However, the latter interventions also increased DALYs through side effects affecting HRQOL or causing death.

Differences in DALYs due to OA in the simulated population for each scenario relative to baseline (DALYs averted) were computed as a difference in years lived with disability (YLDs) plus difference in years of life lost (YLLs) [49]. YLDs for each year were calculated for males and females as OA prevalence rate x disability weight (scenario-specific) x population person-time. Differences in YLLs due to OA for each year were calculated as person-time in each intervention scenario minus person-time in the base-case scenario. For calculating OA-related DALYs, we assumed no effect of BMI reduction on OA-related YLLs. Disability weights were calculated as a difference in mean HUI3 between people with and without OA, separately for males and females. To calculate DALYs averted for each intervention as a proportion of total DALYs, total YLDs in the base-case scenario were calculated as described above and OA-related YLLs were calculated as a difference in person-time between base-case and a scenario in which all effects of drugs and surgery were suppressed. The study was approved by the University of British Columbia Behavioral Research Ethics Board.

## Results

### Simulated open population (base-case scenario)

The sample size for the duration of the simulation was 10 million, resulting in a simulated open population of about 3,600,000 individuals in 2020, or 12% of the Canadian population 20 years of age and older. In the base-case scenario, the projected proportion of person-time in the age group 70+ years increased from 12.6% in males and 15.5% in females in 2020 to 20.0% and 23.4%, respectively, in 2040 (Table 2).

**Table 2:** Descriptive data for the simulated population (base-case scenario).

Variable	Males		Females	
	2020	2040	2020	2040
General population				
Age (%)				
20–69	87.4	80.0	84.5	76.6
≥70	12.6	20.0	15.5	23.4
BMI (%)				
Underweight (<18.5)	1.0	0.9	5.3	5.6
Normal weight (18.5–24.9)	32.7	29.1	44.2	39.7
Overweight (25.0–29.9)	41.6	40.4	29.3	28.6
Obese (≥30.0)	24.7	29.6	21.3	26.1
Osteoarthritis (OA)				
Incidence per 1000	8.1	9.8	11.1	13.3
Prevalence (%)	12.2	15.6	17.0	21.4
People with osteoarthritis				
HUI3				
Mean	0.783	0.757	0.742	0.723
Pain level (%)				
1 (No pain or discomfort)	74.1	74.1	70.4	70.9
2 (Mild pain)	6.0	6.0	6.2	6.1
3 (Moderate pain)	8.6	8.6	9.2	9.2
4 (Moderate-to-severe pain)	7.0	7.0	8.3	8.1
5 (Severe pain)	4.2	4.3	5.8	5.7
Medication use (%)				
NSAIDs	37.7	38.9	38.5	39.4
Acetaminophen	16.6	19.2	24.0	26.9
Coxibs	3.7	3.6	4.4	4.2
Opioids	4.1	3.9	5.0	4.8
Joint replacement surgery (JRS)				
Primary JRS rate per 1000	45.0	55.0	40.1	47.4
Revision JRS rate per 1000	4.0	4.6	3.3	3.6

BMI = Body mass index; HUI3 = Health Utilities Index Mark 3; NSAIDs = Non-steroidal anti-inflammatory drugs; JRS = Joint replacement surgery

In males, the proportion with normal BMI (18.5–24.9) diminished from 32.7% in 2020 to 29.1% in 2040, whereas the proportion obese increased from 24.7% to 29.6%. In females, normal BMI decreased from 44.2% to 39.7% and obese increased from 21.3% to 26.1%. Prevalence of OA showed an increasing trend in both males and females. In 2020, 12.2% of males and 17.0% of females had OA. By 2040, these proportions were projected to increase to 15.6% and 21.4%, respectively.

Among males with OA, mean projected HUI3 decreased from 0.783 in 2020 to 0.757 in 2040 (Table 2). HUI3 was lower in females and showed a similar trend, from 0.742 to 0.723, respectively. In 2020, the majority of males with OA (74.1%) reported no pain or discomfort (pain level 1), 6.0% had pain level 2, 8.6% had pain level 3, 7.0% had pain level 4, and 4.2% had pain level 5. The corresponding proportions in females were 70.4%, 6.2%, 9.2%, 8.3%, and 5.8%. Pain levels remained steady between 2020 and 2040 in both sexes.

In both males and females with OA, the most common pain medication was NSAIDs, followed by acetaminophen, opioids, and coxibs. In 2020, 37.7% of males and 38.5% of females were estimated to use NSAIDs on any given day (prescribed and over-the-counter combined). The corresponding proportions for other medications in males and females were 16.6% and 24.0% for acetaminophen, 4.1% and 5.0% for opioids, and 3.7% and 4.4% for coxibs, respectively. The projected use of acetaminophen increased slightly from 2020 to 2040 in both sexes, whereas changes in the use of other analgesics were minimal (Table 2).

Joint (hip and knee combined) replacement rates among persons with OA in 2020 were higher in males (45.0 per 1000 person-years) compared with females (40.1 per 1000 p-y). From 2020 to 2040, the rates were projected to increase by 22.3% (to 55.0 per 1000) in males and 18.2% (to 47.4 per 1000) in females. Revision rates increased as well during that time, from 4.0 to 4.6 per 1000 in males and from 3.3 to 3.6 in females (Table 2).

### Simulated changes due to interventions

**Fig 1:**
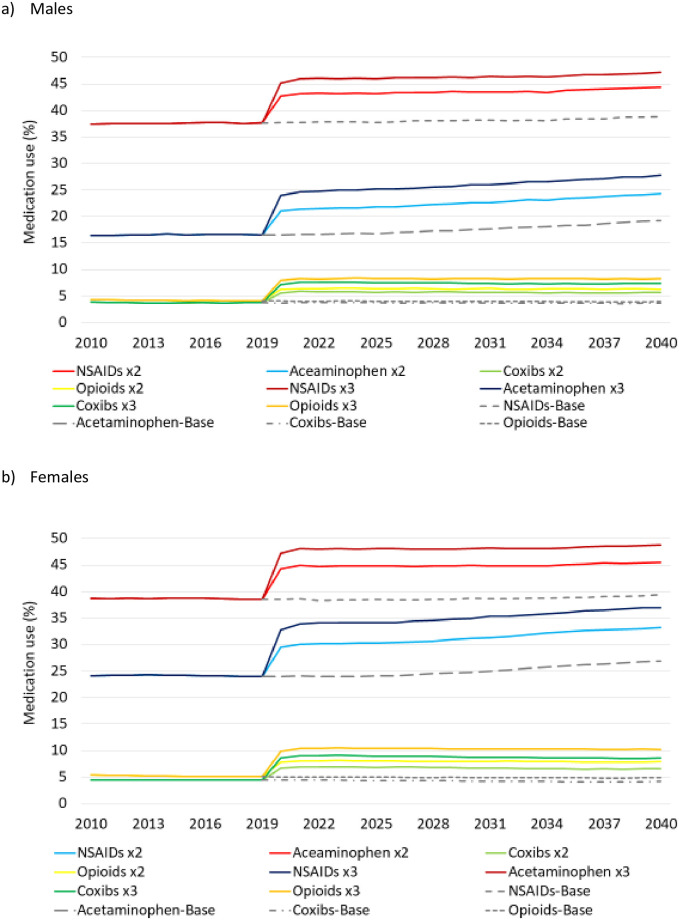
Use of four classes of analgesics before and after Medication x2 and Medication x3 interventions in people with osteoarthritis. All interventions are described in Table 1.

Changes in the use of analgesics as a result of the simulated medical interventions are shown in Fig 1a and 1b. In males with OA, NSAIDs increased to 43.1% in 2021 under the Medication x2 scenario and 46.0% under the Medication x3 scenario, equivalent to relative increases by 14.5% and 22.0%. The use of acetaminophen increased by 29.3% and 49.2%, opioids by 55.4% and 100.8% and coxibs by 55.0% and 102.0%, respectively (Fig 1a). In females with OA, the use of NSAIDs increased to 44.9% and 48.2% under Medication x2 and Medication x3, respectively (relative increase by 16.8% and 25.2%). Of the other analgesics, acetaminophen increased by 25.7% and 41.6%, opioids by 58.9% and 105.3%, and coxibs by 58.2% and 106.8%, respectively (Fig 1b).

**Fig 2:**
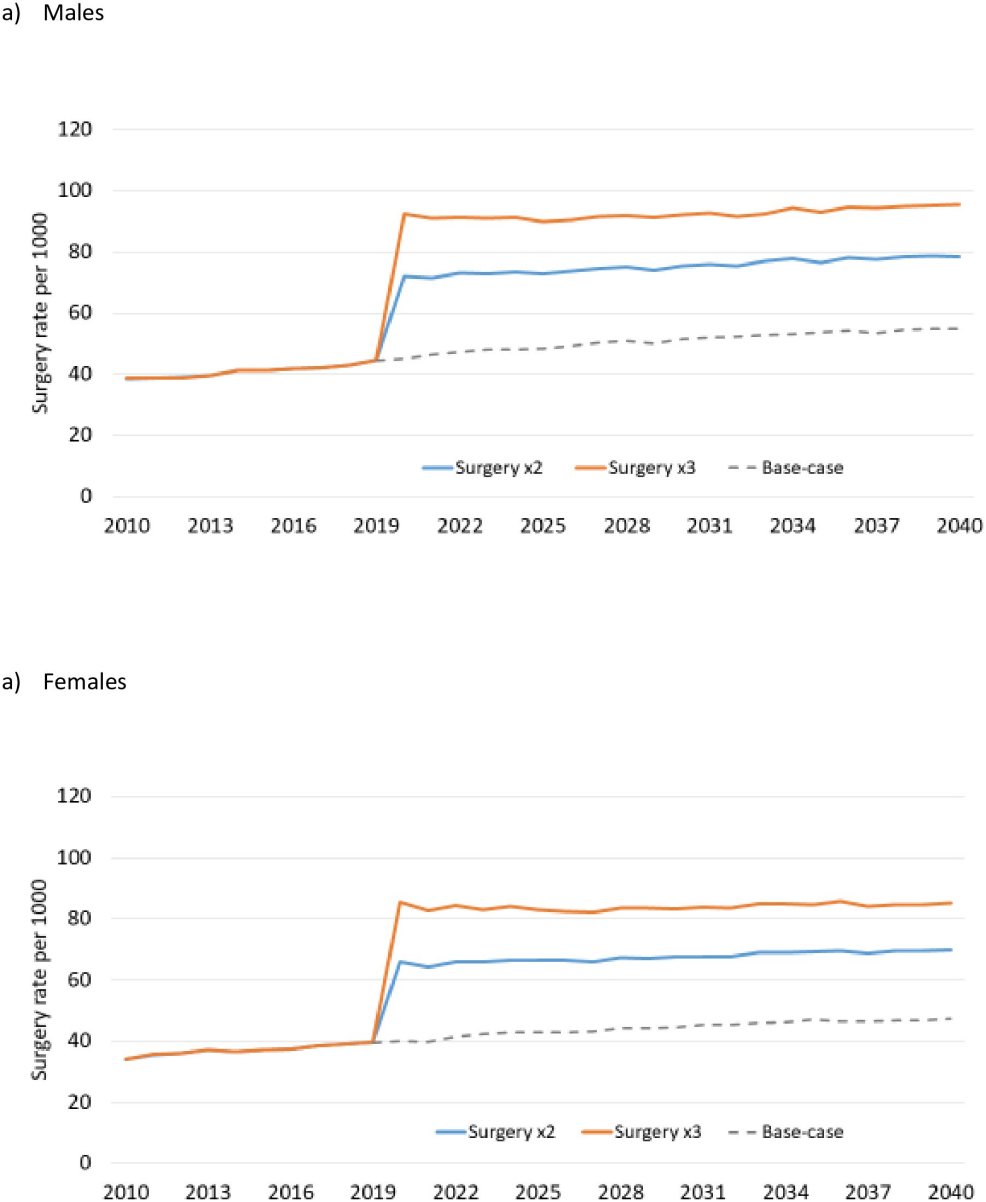
Joint replacement rates before and after Surgery x2 and Surgery x3 interventions in people with osteoarthritis. All interventions are described in Table 1.

Changes in JRS rates resulting from the simulated surgical interventions in persons with OA are described in Fig 2a (males) and Fig 2b (females). In males, the rates increased to 72.0 per 1000 in 2020 (by 61.6%) under Surgery x2 and 92.6 per 1000 (by 107.9%) under Surgery x3. In females, rates increased by 66.6% and 116.3%, respectively, but remained lower than those in males (Fig 2b).

**Fig 3:**
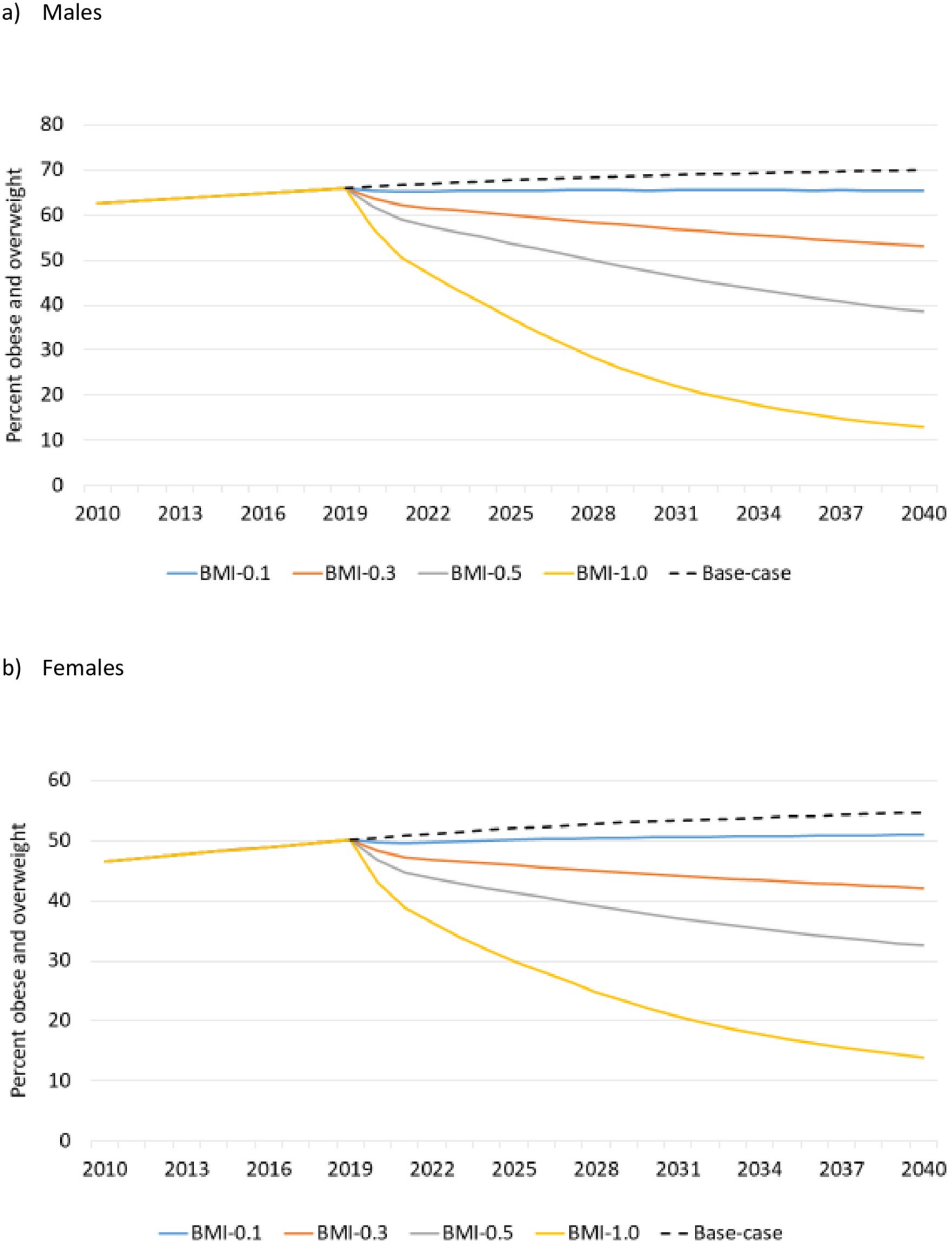
Percentage obese and overweight in the population under four preventive intervention scenarios. All interventions are described in Table 1.

Weight reduction interventions produced gradual changes in the distribution of BMI in the entire population (not just persons with OA) over time, as shown in Fig 3a and 3b. Under the BMI-0.1 scenario, the proportions obese and overweight remained approximately constant in both sexes. With stronger interventions, the proportion declined substantially over time, and the percent with normal BMI increased. The proportion overweight did not change much under BMI-0.3 and BMI-0.5, as persons moving from obese to overweight were offset by those moving from overweight to normal BMI. However, with BMI-1.0, the proportion overweight diminished to 11.5% in 2040 in males and 11.3% in females, whereas obesity was reduced to 1.4% in males and 2.6% in females. Only about 1% of males and 5–6% of females were in the underweight category throughout the study period.

### Comparing the impact of interventions on OA burden

**Fig 4:**
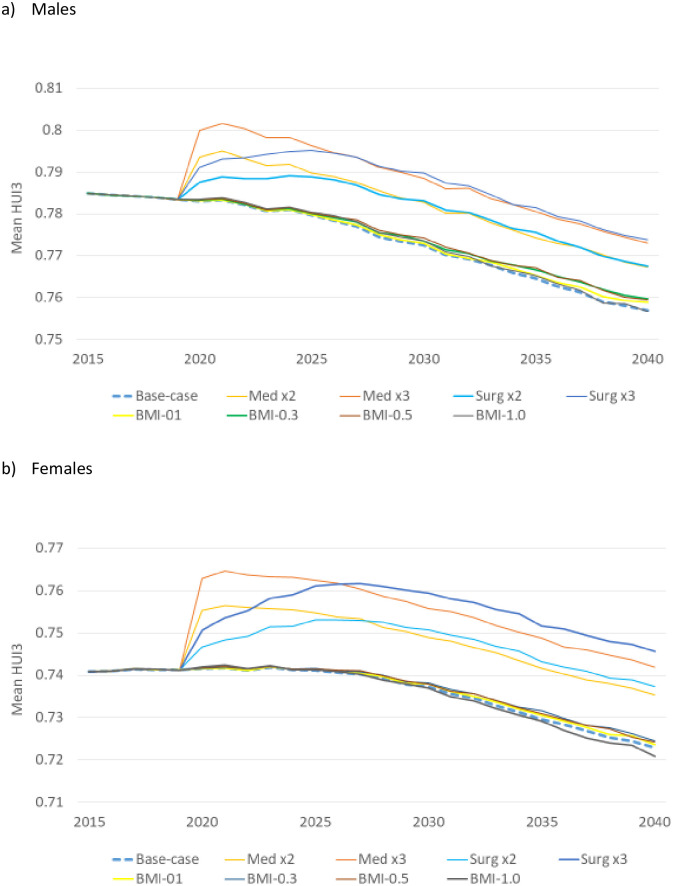
Mean HUI3 among persons with osteoarthritis before and after each intervention. HUI3, Health Utilities Index Mark 3. All interventions are described in Table 1. The trend in HUI3 may appear steep because the Y-axis range was restricted to better visualize the impact of interventions.

The average HUI3 in males with OA increased rapidly in 2020 as a result of the Medication x2 and Medication x3 interventions, achieving a peak in 2021 and resuming its downward trend (largely due to population aging) thereafter, whereas the surgical interventions took longer for a full impact, with a peak in 2024 (Surgery x2) and 2025 (Surgery x3) (Fig 4). In females, the pattern was similar, but the effect of medical and surgical interventions on HUI3 was stronger. In females, both medical interventions achieved a peak in 2021, whereas Surgery x2 achieved a peak in 2026 and Surgery x3 in 2027. BMI reductions had, on average, very little impact on HUI3 in persons with OA.

**Fig 5:**
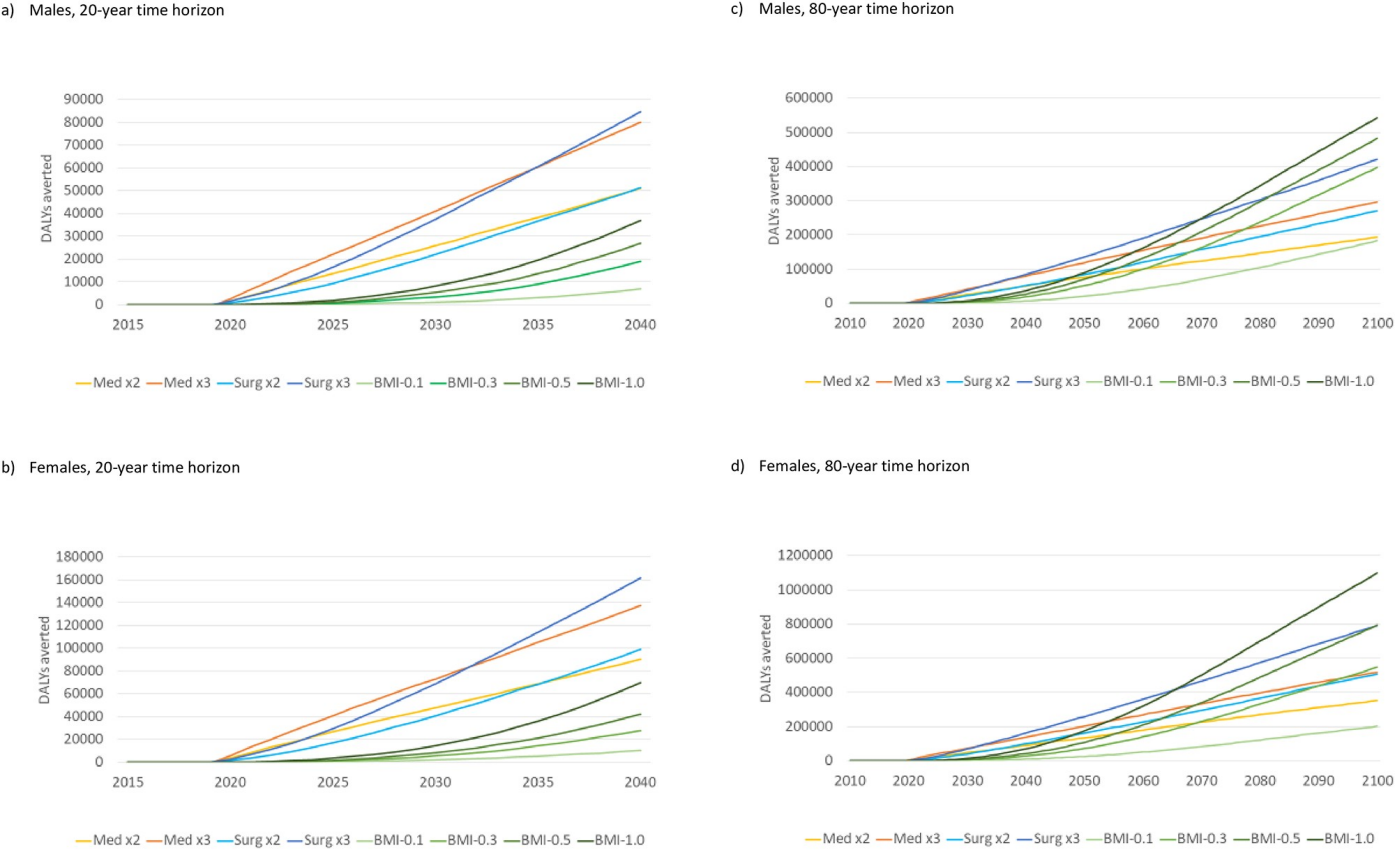
Total cumulative reductions in OA-related DALYs (DALYs averted) from 8 interventions in the simulated population. DALYs, disability-adjusted life years. OA, osteoarthritis. All interventions are described in Table 1.

In Fig 5a and 5b we compare all interventions in terms of OA-related DALYs averted, relative to base-case, for males and females, accumulated over 20 years (2020–2040). In both males and females, Medication x3, which showed a linear accumulation, resulted in more DALYs averted in the first decade than all other interventions. Surgery x3 showed a slower growth initially but a steeper accumulation later on. As a result, Medication x3 was surpassed by Surgery x3 in 2035 in males and 2032 in females. Similarly, Medication x2 was surpassed by Surgery x2 in both males and females before 2040. The impact of preventive interventions in the first two decades of the interventions was relatively small. Reductions in DALYs for all scenarios were greater in females. By 2040, accumulated reductions in the simulated population ranged from 7,107 DALYs averted for BMI-0.1 to 84,657 for Surgery x3 in males, and from 10,277 to 161,615 in females, respectively.

The impact of interventions looked quite different when we extended the time horizon to 80 years (until 2100) as DALYs averted from preventive interventions accumulated much faster than those from treatment interventions over a longer term (Fig 5c and 5d). By the end of the century, the greatest impact in both males and females was achieved by BMI-1.0, followed by BMI-0.5, Surgery x3, BMI-0.3, Medication x3, Surgery x2, Medication x2, and BMI-0.1.

**Fig 6:**
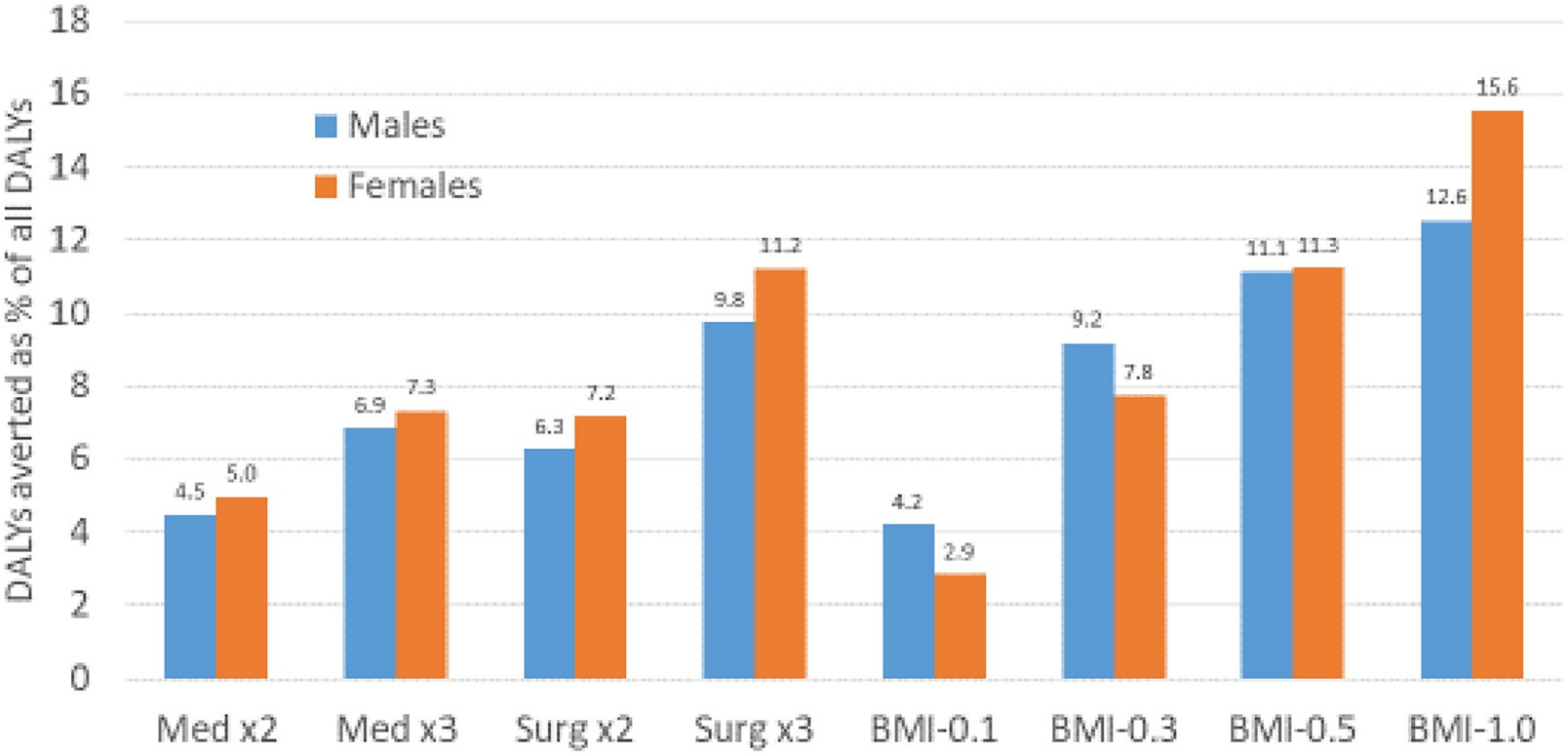
DALYs averted due to 8 interventions as a percentage of all OA-related DALYs, relative to base-case, over an 80-year time horizon. DALYs, disability-adjusted life years. OA, osteoarthritis. All interventions are described in Table 1.

In Fig 6, we show DALYs averted as a percentage of all DALYs due to OA in the population. By 2100, reductions in OA burden were 12.6% in males and 15.6% in females for BMI-1.0, 11.1% and 11.3%, respectively, for BMI-0.5, 9.8% and 11.2% for Surgery x3, 9.2% and 7.8% for BMI-0.3, 6.9 and 7.3% for Medication x3, 6.3% and 7.2% for Surgery x2, 4.5% and 5.0% for Medication x2, and 4.2% and 2.9% for BMI-0.1.

### Sensitivity analysis

Our sensitivity analysis showed a large variation in the impact of different parameters on the results (Fig A3-1—A3-3, Appendix 3 in S1 File). In the BMI scenarios, the relative risk of OA associated with obesity was the most influential parameter, as expected. In both medical and surgical scenarios, the greatest impact was seen for model parameters determining the effect of JRS on pain. These parameters were derived from a relatively small study and had wide confidence intervals. Variation in the effects and side effects of medications, which was estimated from the literature, had relatively little impact on the results, and the impact was difficult to distinguish from random error in the model.

## Discussion

To our knowledge, this is the first population-based comparative simulation study of the potential impact of different burden reduction strategies in OA. Using a previously validated microsimulation model, we compared two medical treatment interventions, two surgical treatment interventions, and four preventive interventions against a base-case scenario. Reductions in DALYs depended on the type and magnitude of the intervention and the time horizon. Among the 8 specific interventions studied, Medication x3 reduced DALYs very quickly through its impact on pain and was, therefore, most effective over a short time horizon (a decade). Surgery x3 took longer to produce large reductions in DALYs, but was more effective than Medication x3 over a longer time horizon (2 decades or more). Preventive interventions did not make a strong impact in the short-to-intermediate term, but BMI-1.0, followed by BMI-0.5, resulted in the largest reductions in DALYs over a very long time horizon (several decades).

Our data provide estimates of how much burden reduction, both in absolute DALY counts and in relative terms, can be expected from an intervention of a given scope and duration, and how quickly the reduction in DALYs can be expected to accumulate. We hope this kind of information may be useful to policymakers in developing and implementing interventions to reduce OA burden within a desired timeframe. We did not, however, model the mechanisms through which the specified changes in medication use, JRS rates, or obesity levels could be realized. Therefore, we are not suggesting that such changes would occur as a result of any specific intervention or policy. Rather, our data can be used to estimate the impact of any intervention of the general type we analyzed through interpolation or extrapolation of our findings. The advantage of this approach compared with modeling a specific intervention is greater generalizability of results.

Our assumption that the medical and surgical interventions would be effective immediately was a simplification. However, this does not imply that the amount of change was implausible. For example, in the Medication x2 intervention, NSAIDs increased by 15% among males and 17% among females with OA. Changes of this magnitude are not unrealistic, as larger fluctuations in the use of some analgesics have been observed [50]. Similarly, surges in JRS rates approaching those assumed in our model actually took place in Canada in the past in response to government policies [51]. With respect to OA prevention, our BMI-0.1 scenario resulted in stable levels of obesity/overweight. Although reducing obesity has been a challenge in Canada and many other countries, obesity rates have stabilized or declined slightly in some countries in the past decade [5]. Examples of population-level interventions whose effects on obesity rates have been modeled include physician counselling, food labeling, fiscal measures, worksite interventions, mass media campaigns, food advertising regulation, and school-based interventions [52].

The results can also be interpreted in terms of equivalence between different types of interventions. For example, Medication x3 and Surgery x3 are equivalent in terms of DALYs averted around 2035, i.e., at 15 years since intervention onset, in males, and around 2032, i.e., at 12 years, in females. Prior to that Medication x3 is more effective and after that Surgery x3 is more effective. Similarly, Medication x2 is equivalent to Surgery x2 around 2039 in males and 2035 in females. In other words, if a 10-year time horizon is important from a policy perspective, a surgical intervention stronger than Surgery x3 is needed to match the effects of Medication x3. We could also infer from Fig 5 that in 2030 (over a 10-year time horizon) Surgery x2 is approximately equivalent to a medical intervention with a multiplier of 1.7 in both sexes, and Surgery x3 is approximately equivalent to a medical intervention with a multiplier of 2.7 in males and 2.8 in females.

Differences in the impact of different strategies with respect to time horizon in our model seem plausible. Pain in OA is often undertreated [53, 54]. On the other hand, for many older patients with relatively mild pain, harms likely outweigh the benefits. Appropriately selective and guideline-consistent pharmacological treatment of pain, with a stronger focus on adherence, would likely bring about improvements in quality of life and potentially reduce the need for surgery.

It is useful to consider the medical treatment strategies analyzed here in the context of current clinical guidelines for the management of OA. Current guidelines recommend the use of NSAIDs; they discourage the use of acetaminophen because of its low effectiveness compared to placebo. They also discourage the use of opioids because of their potential for addiction [9, 10]. We assumed an increase in the use of all 4 types of analgesics, but only in the types of patients who would, on average, benefit from their increased use. In our model, the risks outweighed the benefits of treatment for oral NSAIDs, coxibs, and opioids in older persons with mild pain, hence acetaminophen was the only option in this group.

It is also well-established that many OA patients who would benefit from joint replacement do not receive surgery [55]. At the population level, the effect of increased access to JRS on OA burden could be significant, although it would take a little longer to materialize than the effect of a pharmacological intervention. Our model predicted an average net benefit from hip/knee replacement in all patients with pain, and the two surgical scenarios we considered assumed a proportional increase in JRS rates in all pain levels. However, the individual benefits were greater in those with more severe pain. Hence, for the same number of additional surgeries, reductions in DALYs would be greater if the increase in JRS was restricted to patients with more severe pain. On the other hand, increasing access to surgical treatment may be associated with JRS being performed on patients with less pain. This would result in less benefit than observed in our study. Finally, preventive measures that reduce OA incidence can be effective in the long-term, but require a substantial reduction in obesity, which seems difficult to achieve in most populations at this time.

We are not aware of other population-based simulation studies comparing the impact of pharmacological, surgical, and preventive interventions in OA. Segal et al. compared cost-effectiveness of various treatment and prevention options of OA using the Health-sector Wide model in Australia [56]. They found JRS to be most cost-effective, whereas the net benefits from NSAIDs were close to none and prevention programs could not be evaluated due to limited data. A number of modeling studies examined cost-effectiveness of specific treatments, such as knee and hip arthroplasty [57–59], hip resurfacing [60], viscosupplementation [61], and various pharmacological agents [62–68]. The results of these studies are difficult to compare with our data because our objectives, simulated population, and types of interventions were different, and we did not include costs in the current analyses.

For example, Ponnusamy et al. [59] used a Markov model to estimate the cost-effectiveness of knee replacement against non-operative management among 6 BMI cohorts. Surgical treatment was more expensive but also more effective in obese patients, making it cost-effective in all groups. Losina et al. evaluated the cost-effectiveness of generic celecoxib relative to naproxen using the Osteoarthritis Policy (OAPol) model [67]. The study compared 8 specific medical treatment scenarios in terms of effectiveness, costs, and incremental cost-effectiveness ratios in a simulated cohort of patients with knee OA and severe pain. They found that celecoxib was not cost-effective at its current price. Another application of the OAPol model was a recent study to estimate QALYs lost due to inactivity in the US population aged >45 years with knee OA [69]. What-if scenarios assumed that 5%, 10%, or 20% of the inactive population were instead active. The results demonstrated considerable potential benefits of increased physical activity among persons with knee OA.

Losina and colleagues estimated the burden of knee OA in terms of QALYs lost over lifetime in people aged 50–84 in the US [70]. They found a loss of 1.71 QALYs per person. Abbott et al. reported QALYs lost due to OA in New Zealand to be, depending on the method of measuring health utilities, 3.44 or 1.65 [71]. Although our simulation was designed to compare reductions in OA burden relative to base-case, rather than measure the burden itself, our model did provide an estimate of average lifetime QALYs in persons aged 70+ years with and without OA. The difference was 1.89 QALYs, consistent with the aforementioned studies.

A long history of successful applications of POHEM-based models [16, 17, 72–76] suggests a high degree of credibility of our computer simulation platform. The conceptual structure of MSM-OA has been discussed in prior publications [16, 17, 77]. Sources of data, methods of analysis, and computer implementation are described in Methods and in greater detail in Appendix 1 in S1 File. The distribution of sociodemographic variables in our population at the start of the simulation reflected the household population of Canada in 2001. In 2018, the proportion of persons aged 70+ years under base-case scenario matched the corresponding proportion in the CCHS (13.4% vs. 14.0%, Table A1-19), supporting our demographic projections. For other variables, model projections also generally agree with actual data for the periods when such data are available. Specifically, the distribution of BMI and its changes over time agree with data reported by the Public Health Agency of Canada [78]. OA prevalence in our model is almost identical to OA prevalence observed in the Canadian Chronic Disease Surveillance System [79]. Use of major classes of analgesics in OA in our simulation is similar to that reported by Kingsbury et al. from the Osteoarthritis Initiative in the US [80], despite some differences in populations and methodology. The projected rates of JRS among persons with OA in our model are close to the rates reported by the Canadian Joint Replacement Registry [81].

Although our model is based on the Canadian population, generalizability of our findings extends to other high-income countries. Some key parameters have been obtained from the literature and are likely generalizable across many populations. This applies to the effect of analgesics on pain and frequency of side effects. For the parameters derived from Canadian studies, such as the impact of side effects on HUI3 or the effect of JRS on pain, there is no reason to assume these relationships vary substantially across countries. Even if the absolute changes in DALYs vary due to differences in population age structure, risk factor distribution, or access to and quality of healthcare, it is likely that the relative benefits from different interventions are broadly generalizable [82].

Microsimulation is a powerful methodology that has allowed us to develop a complex (and therefore more realistic) stochastic model of OA, with a large number of parameters (Appendix 1 in S1 File). This is an important strength of our study. However, complexity of the model is also a limitation. In particular, it was not feasible to generate uncertainty bands for the DALY estimates. When interpreting our results it is, therefore, important to acknowledge that the results represent mean values and are subject to uncertainty. This uncertainty comes almost exclusively from parameter error, as Monte Carlo error (due to stochastic nature of the model) is practically negligible. In our sensitivity analysis we only tested a small fraction of all parameters in the model. The analysis demonstrated that uncertainty is much greater for some parameters than others, and this translates into a greater potential impact on the outcomes of the study. Generally, variation in outcomes associated with parameter uncertainty was significantly less than the effect of the interventions. Nonetheless, when extrapolating our data to interventions with smaller effects, the level of uncertainty in model parameters we observed need to be taken into consideration. Uncertainty could potentially be reduced in the future by replacing parameters derived from relatively small studies with more robust parameters from published meta-analyses, when such data become available.

Other limitations of the study should be mentioned. We did not consider topical NSAIDs and non-pharmacological therapies such as exercise, both of which are strongly recommended by current guidelines [9–11]. The main reason was a lack of data on the use of these modalities in our databases. Given the strong evidence of the benefits of exercise, especially in knee OA, in terms of both pain reduction and functional improvement, and a very favorable safety profile compared with most pharmacological therapies, modeling of the population impact of exercise-based interventions should be a priority. Data limitations also did not allow us to build separate models of OA of different joints and to account for OA of multiple joints. In addition, our BMI estimates were based on self-reported weight and height, which are known to be less accurate than measured values.

In developing and selecting disease burden reducing policies, cost of the intervention is often an important consideration. Our study was designed to compare effectiveness rather than cost-effectiveness of interventions and we did not include costs in our analysis. However, since we have provided a detailed description of each what-if scenario, our results can be used in future studies to perform cost-effectiveness analyses.

In summary, we have for the first time projected and compared the population health impact of different OA treatment and prevention strategies. We have demonstrated that comparable reductions in OA burden can be achieved by improving pharmacological therapy, increasing access to JRS, and reducing obesity. We have also shown how burden reduction depends on the magnitude of each intervention and the time horizon for assessing impact. We hope these results might help healthcare providers and policymakers in developing strategies for addressing the population health impact of the OA pandemic.

## Supporting information

S1 File (PDF)

## References

[1] Lawrence RC, Felson DT, Helmick CG, Arnold LM, Choi H, Deyo RA, et al. Estimates of the prevalence of arthritis and other rheumatic conditions in the United States. Part II. Arthritis Rheum. 2008;58(1):26–35. doi: 10.1002/art.23176 18163497 PMC3266664

[2] Kopec JA, Rahman MM, Berthelot J-M, Le Petit C, Aghajanian J, Sayre EC et al. Descriptive epidemiology of osteoarthritis in British Columbia, Canada. J Rheumatol 2007;34(2):386–93 17183616

[3] Safiri S, Kolahi AA, Smith E, Hill C, Bettampadi D, Mansournia MA et al. Global, regional and national burden of osteoarthritis 1990–2017: a systematic analysis of the Global Burden of Disease Study 2017. Ann Rheum Dis. 2020;79(6):819–828. doi: 10.1136/annrheumdis-2019-216515 32398285

[4] St Sauver JL, Warner DO, Yawn BP, Jacobson DJ, McGree ME, Pankratz JJ et al. Why patients visit their doctors: assessing the most prevalent conditions in a defined American population. Mayo Clin Proc 2013;88(1):56–67. doi: 10.1016/j.mayocp.2012.08.020 23274019 PMC3564521

[5] Global Burden of Disease; Institute of Health Metrix and Evaluation. GBD Compare website. https://vizhub.healthdata.org/gbd-compare/. Accessed: October 7, 2020.

[6] Lidgren L, Smolen J, Bentley G, Delmas P. European action towards better musculoskeletal health. A public health strategy to reduce the burden of musculoskeletal conditions. Turning evidence into everyday practice. 2000. http://ec.europa.eu/health/ph_projects/2000/promotion/fp_promotion_2000_frep_15_en.pdf. Accessed: October 7, 2020.

[7] Arthritis Foundation, Center for Disease Control and Prevention. A national public health agenda for osteoarthritis. 2010. http://www.cdc.gov/arthritis/docs/oaagenda.pdf. Accessed: October 7, 2020.

[8] Arthritis Alliance of Canada. The Impact of Arthritis in Canada: Today and Over the Next 30 Years. 2011. www.arthritisalliance.ca. Accessed: October 7, 2020.

[9] Fernandes L, Hagen KB, Bijlsma JW, Andreassen O, Christensen P, Conaghan PG et al. European League Against Rheumatism (EULAR). EULAR recommendations for the non-pharmacological core management of hip and knee osteoarthritis. Ann Rheum Dis. 2013;72(7):1125–35. doi: 10.1136/annrheumdis-2012-202745 23595142

[10] Bannuru RR, Osani MC, Vaysbrot EE, Arden NK, Bennell K, Bierma-Zeinstra SMA et al. OARSI guidelines for the non-surgical management of knee, hip, and polyarticular osteoarthritis. Osteoarthritis Cartilage. 2019;27(11):1578–1589. doi: 10.1016/j.joca.2019.06.011 31278997

[11] Kolasinski SL, Neogi T, Hochberg MC, Oatis C, Guyatt G, Block J et al. 2019 American College of Rheumatology/Arthritis Foundation guideline for the management of osteoarthritis of the hand, hip, and knee. Arthritis Rheumatol. 2020;72(2):220–233 doi: 10.1002/art.41142 31908163 PMC10518852

[12] Quinn RH, Murray J, Pezold R, Hall Q. Management of osteoarthritis of the hip. J Am Acad Orthop Surg. 2018;26(20):e434–e436. doi: 10.5435/JAAOS-D-18-00351 30134309

[13] Quinn RH, Murray J, Pezold R, Hall Q, Sevarino KS. Surgical management of osteoarthritis of the knee. J Am Acad Orthop Surg 2018;26(9):e191–e193. doi: 10.5435/JAAOS-D-17-00424 29688919

[14] Spitaels D, Vankrunkelsven P, Desfosses J, Luyten F, Verschueren S, Van Assche D et al. Barriers for guideline adherence in knee osteoarthritis care: A qualitative study from the patients’ perspective. J Eval Clin Pract. 2017 Feb;23(1):165–172. doi: 10.1111/jep.12660 27859970

[15] Meiyappan KP, Cote MP, Bozic KJ, Halawi MJ. Adherence to the American Academy of Orthopaedic Surgeons clinical practice guidelines for nonoperative management of knee osteoarthritis. J Arthroplasty 2020;35(2):347–352. doi: 10.1016/j.arth.2019.08.051 31563393

[16] Kopec JA, Sayre EC, Flanagan WM, Fines P, Cibere J, Rahman MM et al. Development of a population-based microsimulation model of osteoarthritis in Canada. Osteoarthritis Cartilage. 2010;18(3):303–11. doi: 10.1016/j.joca.2009.10.010 19879999

[17] Sharif B, Kopec J, Bansback N, Rahman MM, Flanagan WM, Wong H et al. Projecting the direct cost burden of osteoarthritis in Canada using a microsimulation model. Osteoarthritis Cartilage. 2015;23(10):1654–63. doi: 10.1016/j.joca.2015.05.029 26050868

[18] Wolfson MC. POHEM—a framework for understanding and modelling the health of human populations. World Health Stat Q 1994;47:157–76. 7740830

[19] Statistics Canada. Canadian Community Health Survey. Detailed information for 2000–2001 (Cycle 1.1). https://www23.statcan.gc.ca/imdb/p2SV.pl?Function=getSurvey&Id=3359. Accessed: October 7, 2020

[20] George MV, Loh S, Verma RBP, Shin YE. Population Projections for Canada, Provinces and Territories 2000e2026. Statistics Canada, Demography Division. Catalogue no. 91-520-XPB ISBN 0-660-60634-8. Ministry of Industry; 2001. http://www.statcan.gc.ca/pub/91-520-x/91-520-x2010001-eng.htm. Accessed: October 7, 2020

[21] Statistics Canada. National Population Health Survey (NPHS) http://www.statcan.gc.ca/eng/survey/household/3225. Accessed: October 8, 2020.

[22] Ark TK, Kesselring S, Hills B, McGrail KM. Population Data BC: Supporting population data science in British Columbia. Int J Popul Data Sci. 2020;26;4(2):1133. doi: 10.23889/ijpds.v5i1.1133 32935036 PMC7480325

[23] Rahman MM, Cibere J, Goldsmith CH, Anis AH, Kopec JA. Osteoarthritis incidence and trends in administrative health records from British Columbia, Canada. J Rheumatol. 2014;41(6):1147–54 doi: 10.3899/jrheum.131011 24737915

[24] Feeny D, Furlong W, Torrance GW, Goldsmith CH, Zhu Z, DePauw S, et al. Multiattribute and single-attribute utility functions for the health utilities index mark 3 system. Med Care. 2002;40(2):113–28. doi: 10.1097/00005650-200202000-00006 11802084

[25] British Columbia Ministry of Health [creator] (2011): PharmaCare. British Columbia Ministry of Health [publisher]. Data Extract. MOH (2011). Internet address: http://www2.gov.bc.ca/gov/content/health/conducting-health-research-evaluation/data-access-health-data-central.

[26] Martín Arias LH, Martín González A, Sanz Fadrique R, Vazquez ES. Cardiovascular risk of nonsteroidal anti-inflammatory drugs and classical and selective cyclooxygenase-2 inhibitors: a meta-analysis of observational studies. J Clin Pharmacol. 2019;59(1):55–73. doi: 10.1002/jcph.1302 30204233

[27] Martín Arias LH, Martín González A, Sanz Fadrique R, Salgueiro Vázquez E. Gastrointestinal safety of coxibs: systematic review and meta-analysis of observational studies on selective inhibitors of cyclo-oxygenase 2. Fundam Clin Pharmacol. 2019;33(2):134–147. doi: 10.1111/fcp.12430 30383903

[28] Bally M, Dendukuri N, Rich B, Nadeau L, Helin-Salmivaara A, Garbe E et al. Risk of acute myocardial infarction with NSAIDs in real world use: Bayesian meta-analysis of individual patient data. BMJ. 2017;357:j1909. doi: 10.1136/bmj.j1909 28487435 PMC5423546

[29] Schmidt M, Sørensen HT, Pedersen L. Diclofenac use and cardiovascular risks: series of nationwide cohort studies. BMJ. 2018;362:k3426. doi: 10.1136/bmj.k3426 30181258 PMC6122252

[30] Coxib and traditional NSAID Trialists’ (CNT) Collaboration, Bhala N, Emberson J, Merhi A, Abramson S, Arber N, et al. Vascular and upper gastrointestinal effects of non-steroidal anti-inflammatory drugs: meta-analyses of individual participant data from randomised trials. Lancet. 2013;31;382(9894):769–79. doi: 10.1016/S0140-6736(13)60900-9 23726390 PMC3778977

[31] Schink T, Kollhorst B, Varas Lorenzo C, Arfè A, Herings R, Lucchi S et al. Risk of ischemic stroke and the use of individual non-steroidal anti-inflammatory drugs: A multi-country European database study within the SOS Project. PLoS One. 2018;13(9):e0203362. doi: 10.1371/journal.pone.0203362 30231067 PMC6145581

[32] Solomon DH, Rassen JA, Glynn RJ, Lee J, Levin R, Schneeweiss S. The comparative safety of analgesics in older adults with arthritis. Arch Intern Med. 2010 Dec 13;170(22):1968–76. doi: 10.1001/archinternmed.2010.391 21149752

[33] Makris UE, Kohler MJ, Fraenkel L. Adverse effects of topical nonsteroidal antiinflammatory drugs in older adults with osteoarthritis: a systematic literature review. J Rheumatol. 2010;37(6):1236–43. doi: 10.3899/jrheum.090935 20360183 PMC2880214

[34] Zeng C, Dubreuil M, LaRochelle MR, Lu N, Wei J, Choi HK et al. Association of tramadol with all-cause mortality among patients with osteoarthritis. JAMA. 2019 Mar 12;321(10):969–982. doi: 10.1001/jama.2019.1347 30860559 PMC6439672

[35] Belzak L, Halverson J. Evidence synthesis: The opioid crisis in Canada: a national perspective. Health Promot Chronic Dis Prev Can 2018;38(6):224–233. doi: 10.24095/hpcdp.38.6.02 29911818 PMC6034966

[36] Government of Canada. Opioid-related harms in Canada (September 2020). https://health-infobase.canada.ca/substance-related-harms/opioids/. Accessed: November 20, 2020.

[37] Garbuz DS, Xu M, Sayre EC. Patients’ outcome after total hip arthroplasty: a comparison between the Western Ontario and McMaster Universities index and the Oxford 12-item hip score. J Arthroplasty. 2006;21(7):998–1004. doi: 10.1016/j.arth.2006.01.014 17027542

[38] Kopec JA, Sayre EC, Cibere J, Li LC, Wong H, Bansback N et al. Health-related quality of life and total knee or hip replacement in persons with osteoarthritis: A case-control study. Presented at the 21st World Congress of Epidemiology, Saitama, Japan, August 19–22, 2017.

[39] Kang W, Atashband S, Aghajanian J, Bansback N, Rahman MM, Sayre EC et al. Direct comparison of efficacy of the four major categories of drugs in the treatment of osteoarthritis. Osteoarthritis Cartilage 2007;15(Suppl C):C155 (Abstract). Presented at the Annual Meeting of the Osteoarthritis Research Society International, Ft. Lauderdale, December 6–9, 2007.

[40] Stewart M, Cibere J, Sayre EC, Kopec JA. Efficacy of commonly prescribed analgesics in the management of osteoarthritis: a systematic review and meta-analysis. Rheumatol Int. 2018;38(11):1985–199. doi: 10.1007/s00296-018-4132-z 30120508

[41] Aro P, Talley NJ, Agréus L, Johansson SE, Bolling-Sternevald E, Storskrubb T et al. Functional dyspepsia impairs quality of life in the adult population. Aliment Pharmacol Ther. 2011;33(11):1215–24. doi: 10.1111/j.1365-2036.2011.04640.x 21443537

[42] Groeneveld PW, Lieu TA, Fendrick AM, Hurley LB, Ackerson LM, Levin TR et al. Quality of life measurement clarifies the cost-effectiveness of Helicobacter pylori eradication in peptic ulcer disease and uninvestigated dyspepsia. Am J Gastroenterol. 2001;96(2):338–47. doi: 10.1111/j.1572-0241.2001.03516.x 11232673

[43] Wang YR, Richter JE, Dempsey DT. Trends and outcomes of hospitalizations for peptic ulcer disease in the United States, 1993 to 2006. Ann Surg. 2010;251(1):51–8. doi: 10.1097/SLA.0b013e3181b975b8 20009753

[44] Quan S, Frolkis A, Milne K, Molodecky N, Yang H, Dixon E et al. Upper-gastrointestinal bleeding secondary to peptic ulcer disease: incidence and outcomes. World J Gastroenterol. 2014;20(46):17568–77. doi: 10.3748/wjg.v20.i46.17568 25516672 PMC4265619

[45] Berstock JR, Beswick AD, Lenguerrand E, Whitehouse MR, Blom AW. Mortality after total hip replacement surgery: A systematic review. Bone Joint Res. 2014;3(6):175–82. doi: 10.1302/2046-3758.36.2000239 24894596 PMC4054013

[46] Lie SA, Pratt N, Ryan P, Engesaeter LB, Havelin LI, Furnes O et al. Duration of the increase in early postoperative mortality after elective hip and knee replacement. J Bone Joint Surg Am. 2010;92(1):58–63. doi: 10.2106/JBJS.H.01882 20048096

[47] Hunt LP, Ben-Shlomo Y, Clark EM, Dieppe P, Judge A, MacGregor AJ et al. National Joint Registry for England, Wales and Northern Ireland. 90-day mortality after 409,096 total hip replacements for osteoarthritis, from the National Joint Registry for England and Wales: a retrospective analysis. Lancet. 2013;382(9898):1097–104. doi: 10.1016/S0140-6736(13)61749-3 24075049

[48] Kopec JA, Sayre EC, Fines P, Flanagan WM, Nadeau C, Okhmatovskaia A et al; Simulation Technology for Applied Research Team. Effects of reductions in body mass index on future osteoarthritis burden in Canada: A population-based microsimulation study. Arthritis Care Res (Hoboken). 2016; 68(8):1098–105. doi: 10.1002/acr.22796 26606744 PMC5023424

[49] Murray CJ, Ezzati M, Flaxman AD, Lim S, Lozano R, Michaud C et al. GBD 2010: design, definitions, and metrics. Lancet. 2012;380(9859):2063–6. doi: 10.1016/S0140-6736(12)61899-6 23245602

[50] Kopec J, Cibere J, Lu N, Xie H, Avina-Zubieta J, Esdaile J. Trends in prescribing of NSAIDs and opioids among osteoarthritis patients in British Columbia, Canada, 1998–2014. 2019 American College of Rheumatology ACR/AHRP Annual Scientific Meeting, Atlanta, GA, USA, November 8–13, 2019. Arthritis Rheumatol. 2019; 71 (suppl 10). Abstract # 2202.

[51] Bohm ER, Dunbar MJ, Bourne R. The Canadian Joint Replacement Registry-what have we learned? Acta Orthop. 2010;81(1):119–21. doi: 10.3109/17453671003685467 20170434 PMC2856215

[52] Cecchini M, Sassi F, Lauer JA, Lee YY, Guajardo-Barron V, Chisholm D. Tackling of unhealthy diets, physical inactivity, and obesity: health effects and cost-effectiveness. Lancet. 2010;20;376(9754):1775–84. doi: 10.1016/S0140-6736(10)61514-0 21074255

[53] Marcum ZA, Perera S, Donohue JM, Boudreau RM, Newman AB, Ruby CM et al. Health, aging and body composition study. Analgesic use for knee and hip osteoarthritis in community-dwelling elders. Pain Med. 2011;12(11):1628–36. doi: 10.1111/j.1526-4637.2011.01249.x 21992521 PMC3221937

[54] Knoop J, van Tunen J, van der Esch M, Roorda LD, Dekker J, van der Leeden M et al. Analgesic use in patients with knee and/or hip osteoarthritis referred to an outpatient center: a cross-sectional study within the Amsterdam Osteoarthritis Cohort. Rheumatol Int. 2017;37(10):1747–1755. doi: 10.1007/s00296-017-3785-3 28821939

[55] Hawker GA, Wright JG, Badley EM, Coyte PC. Perceptions of, and willingness to consider, total joint arthroplasty in a population-based cohort of individuals with disabling hip and knee arthritis. Arthritis Rheum. 2004;51(4):635–41. doi: 10.1002/art.20524 15334438

[56] Segal L, Day SE, Chapman AB, Osborne RH. Can we reduce disease burden from osteoarthritis? Med J Aust. 2004;180(S5):S11–7. doi: 10.5694/j.1326-5377.2004.tb05907.x 14984357

[57] Losina E, Walensky RP, Kessler CL, Emrani PS, Reichmann WM, Wright EA et al. Cost-effectiveness of total knee arthroplasty in the United States: patient risk and hospital volume. Arch Intern Med. 2009;169(12):1113–21. doi: 10.1001/archinternmed.2009.136 19546411 PMC2731300

[58] Daigle ME, Weinstein AM, Katz JN, Losina E. The cost-effectiveness of total joint arthroplasty: a systematic review of published literature. Best Pract Res Clin Rheumatol. 2012;26(5):649–58. doi: 10.1016/j.berh.2012.07.013 23218429 PMC3879923

[59] Ponnusamy KE, Vasarhelyi EM, McCalden RW, Somerville LE, Marsh JD. Cost-effectiveness of total hip arthroplasty versus nonoperative management in normal, overweight, obese, severely obese, morbidly obese, and super obese patients: A Markov model. J Arthroplasty. 2018;33(12):3629–3636. doi: 10.1016/j.arth.2018.08.023 30266324

[60] Heintzbergen S, Kulin NA, Ijzerman MJ, Steuten LM, Werle J, Khong H et al. Cost-utility of metal-on-metal hip resurfacing compared to conventional total hip replacement in young active patients with osteoarthritis. Value Health. 2013;16(6):942–52. doi: 10.1016/j.jval.2013.06.021 24041344

[61] Castro JC, Daza AM, Misas JD. Cost-effectiveness Analysis of Viscosupplementation versus Conventional Supportive Therapy for Knee Osteoarthritis in Colombia. Value Health Reg Issues. 2015;8:56–61. doi: 10.1016/j.vhri.2015.03.018 29698172

[62] Katz JN, Smith SR, Collins JE, Solomon DH, Jordan JM, Hunter DJ et al. Cost-effectiveness of nonsteroidal anti-inflammatory drugs and opioids in the treatment of knee osteoarthritis in older patients with multiple comorbidities. Osteoarthritis Cartilage. 2016;24(3):409–18. doi: 10.1016/j.joca.2015.10.006 26525846 PMC4761310

[63] Kamath CC, Kremers HM, Vanness DJ, O’Fallon WM, Cabanela RL, Gabriel SE. The cost-effectiveness of acetaminophen, NSAIDs, and selective COX-2 inhibitors in the treatment of symptomatic knee osteoarthritis. Value Health. 2003;6(2):144–57. doi: 10.1046/j.1524-4733.2003.00215.x 12641865

[64] Marshall JK, Pellissier JM, Attard CL, Kong SX, Marentette MA. Incremental cost-effectiveness analysis comparing rofecoxib with nonselective NSAIDs in osteoarthritis: Ontario Ministry of Health perspective. Pharmacoeconomics. 2001;19(10):1039–49. doi: 10.2165/00019053-200119100-00005 11735672

[65] Smith SR, Katz JN, Collins JE, Solomon DH, Jordan JM, Suter LG et al. Cost-effectiveness of tramadol and oxycodone in the treatment of knee osteoarthritis. Arthritis Care Res (Hoboken). 2017;69(2):234–242. doi: 10.1002/acr.22916 27111538 PMC5378156

[66] Losina E, Daigle ME, Suter LG, Hunter DJ, Solomon DH, Walensky RP et al. Disease-modifying drugs for knee osteoarthritis: can they be cost-effective? Osteoarthritis Cartilage. 2013;21(5):655–67. doi: 10.1016/j.joca.2013.01.016 23380251 PMC3670115

[67] Losina E, Usiskin IM, Smith SR, Sullivan JK, Smith KC, Hunter DJ et al. Cost-effectiveness of generic celecoxib in knee osteoarthritis for average-risk patients: a model-based evaluation. Osteoarthritis Cartilage. 2018;26(5):641–650. doi: 10.1016/j.joca.2018.02.898 29481917 PMC6334297

[68] Bruyère O, Detilleux J, Reginster JY. Cost-effectiveness assessment of different glucosamines in patients with knee osteoarthritis: a simulation model adapted to Germany. Curr Aging Sci. 2021 Apr 14. doi: 10.2174/1874609814666210415092845 Online ahead of print. 33858318

[69] Losina E, Silva GS, Smith KC, Collins JE, Hunter DJ, Shrestha S et al. Quality-adjusted life-years lost due to physical inactivity in a us population with osteoarthritis. Arthritis Care Res (Hoboken). 2020;72(10):1349–1357. doi: 10.1002/acr.24035 31350803 PMC6982563

[70] Losina E, Walensky RP, Reichmann WM, Holt HL, Gerlovin H, Solomon DH et al. Impact of obesity and knee osteoarthritis on morbidity and mortality in older Americans. Ann Intern Med. 2011;154(4):217–26. doi: 10.7326/0003-4819-154-4-201102150-00001 21320937 PMC3260464

[71] Abbott JH, Usiskin IM, Wilson R, Hansen P, Losina E. The quality-of-life burden of knee osteoarthritis in New Zealand adults: A model-based evaluation. PLoS One. 2017;12(10):e0185676. doi: 10.1371/journal.pone.0185676 29065119 PMC5655469

[72] Will BP, Berthelot J-M, Nobrega KM, Flanagan W, Evans WK. Canada’s Population Health Model (POHEM): A tool for performing economic evaluations of cancer control interventions. Eur J Cancer 2001; 37: 1797–1804. doi: 10.1016/s0959-8049(01)00204-0 11549434

[73] Berthelot J-M, Will BP, Evans WK, Coyle D, Earle CC, Bordeleau L. Decision framework for chemotherapeutic interventions for metastatic non-small cell lung cancer. J Natl Cancer Inst 2000; 92(16):1321–29. doi: 10.1093/jnci/92.16.1321 10944554

[74] Flanagan W, Le Petit C, Berthelot J-M, White KJ, Coombs BA, Jones-McLean E. Potential impact of population-based colorectal cancer screening in Canada. Chronic Dis Can 2003: 24(4): 81–88. 14733756

[75] Hennessy DA, Flanagan WM, Tanuseputro P, Bennett C, Tuna M, Kopec J et al. The Population Health Model (POHEM): an overview of rationale, methods and applications. Popul Health Metr. 2015;13:24. doi: 10.1186/s12963-015-0057-x 26339201 PMC4559325

[76] Manuel DG, Garner R, Finès P, Bancej C, Flanagan W, Tu K et al. Alzheimer’s and other dementias in Canada, 2011 to 2031: a microsimulation Population Health Modeling (POHEM) study of projected prevalence, health burden, health services, and caregiving use. Popul Health Metr. 2016;14:37. doi: 10.1186/s12963-016-0107-z 27822143 PMC5095994

[77] Kopec JA, Finès P, Manuel DG, Buckeridge DL, Flanagan WM, Oderkirk J et al. Validation of population-based disease simulation models: a review of concepts and methods. BMC Public Health. 2010;10:710. doi: 10.1186/1471-2458-10-710 21087466 PMC3001435

[78] Public Health Agency of Canada and the Canadian Institute for Health Information. Obesity in Canada: A joint report from the Public Health Agency of Canada and the Canadian Institute for Health Information. © Her Majesty the Queen in Right of Canada, 2011, Cat.: HP5-107/2011E-PDF ISBN: 978-1-100-18133-2. http://www.phac-aspc.gc.ca/hp-ps/hl-mvs/oic-oac/assets/pdf/oic-oac-eng.pdf. Accessed on October 7, 2020.

[79] Government of Canada. Canadian Chronic Disease Surveillance System (CCDSS). https://health-infobase.canada.ca/ccdss/data-tool/. Accessed on October 7, 2020.

[80] Kingsbury SR, Hensor EM, Walsh CA, Hochberg MC, Conaghan PG. How do people with knee osteoarthritis use osteoarthritis pain medications and does this change over time? Data from the Osteoarthritis Initiative. Arthritis Res Ther. 2013;15(5):R106. doi: 10.1186/ar4286 24008023 PMC3978852

[81] Canadian Institute for Health Information. Hip and knee replacements in Canada: CJRR Annual Statistics Summary, 2018–2019. Ottawa, ON; CIHI; August, 2020.

[82] National Research Council. 1991. Improving Information for Social Policy Decisions—The Uses of Microsimulation Modeling: Volume I, Review and Recommendations. Washington, DC: The National Academies Press. 10.17226/1835. Accessed on: October 8, 2020.

