# Supplementary material for: A comparison of three strategies to reduce the burden of osteoarthritis: A population-based microsimulation study
Source: PLoS One. 2021 Dec 8;16(12):e0261017. doi: 10.1371/journal.pone.0261017 (PMC8654220; doi:10.1371/journal.pone.0261017)
Supplement: S1 File — (PDF) [file pone.0261017.s001.pdf]

Supporting information

Appendix 1: Model parameters, derivation methods, and data sources

Initial population

The initial population in MSM-OA was derived from the 2001 cycle of the Canadian Community Health Survey (CCHS) [19]. The distribution of all variables in CCHS (public use data) is available from Statistics Canada at:

https://www23.statcan.gc.ca/imdb/p2SV.pl?Function=getSurvDocument&Item_Id=43635&InstaId=3359

Weighted data reflect the distribution in the Canadian household population. For the demographic variables, the distributions have been provided in a previous publication [16]. The MSM-OA population is an open population consisting of persons 20 years of age and older. Throughout the simulation run, people can exit the population through death or emigration and enter it by becoming 20-years old or immigrating into it. In the current study, the initial population (2001) was composed of about 2,800,000 simulated individuals.

Baseline death rates

Age and sex-specific death rates for Canada in MSM-OA were modeled using Statistics Canada's mortality data and projections [20], as implemented in the Population Health Model (POHEM) microsimulation platform. These projections have been used in many published studies [e.g., 72-77]. Death rates for selected ages and years are shown in Table A1-1. Please note that the projected death rates are frozen after the year 2026.

Body Mass Index (BMI)

The BMI model that we used in MSM-OA had been developed at Statistics Canada using biennial data from the longitudinal National Population Health Survey 1994-2006 [21]. The model predicts current

BMI separately for men and women, based on BMI history and other covariates, such as region of residence, income, and education. The response variable is log BMI, treated as a continuous, normally distributed variable. The model includes the most recent BMI value (2 years back) and up to 3 earlier BMI values, i.e., 4, 6 and 8 years back. The model can be presented as:

$$\text{Log}(BMI_t) = b_0 + b_1 \log(\text{BMI history}) + b_2\,\text{Age} + b_3\,\text{Education} + b_4\,\text{Income} + b_5\,\text{Region} + \text{error}(t).$$

In this equation, BMI history may include BMI_{t-2}, BMI_{t-4}, BMI_{t-6}, and BMI_{t-8}, depending on data availability. There are regression equations separately for males and females. Within each sex stratum, there are four models, depending on the number of prior BMI values used, resulting in 8 models (equations). The parameters (regression coefficients) are shown in Table A1-2.

Baseline OA incidence rates

Age and sex-specific baseline OA incidence rates for MSM-OA were derived from administrative data in British Columbia (BC). A random sample of 742,070 records was obtained from Population Data BC, an administrative database which covers virtually all hospitalizations and visits to health professionals in BC since 1986. Similar to prior studies [16,23], we defined incident OA as a hospital discharge or two visits to a health professional within 2 years (and not on the same day) with a diagnostic code for OA (715 in ICD-9 or M15-M19 in ICD-10) in a person without OA within the previous 10 years. Visits to all types of health professionals were included. The date of the second code was the date of diagnosis. Person-years were obtained from individual data on registration with the provincial health plan. Baseline OA incidence rates by age and sex are shown in Table A1-3.

Initial prevalence of OA

The initial age/sex-specific prevalence of OA was calibrated in a simulation run using the incidence rates from Population Data BC (Table A1-3), such that the population was in a steady state at the beginning of

the simulation [16]. To ensure that the distribution of BMI by OA status in the initial population was correct, we assigned OA to subjects who self-reported OA in the CCHS. In the age/sex groups in which the proportion self-reporting OA was greater than the calibrated prevalence from administrative data, we randomly reduced the number of persons with OA to match the calibrated prevalence, and in the age/sex groups in which the proportion self-reporting OA was smaller than the calibrated prevalence, we randomly increased the number with OA. The proportion of subjects in different stages of OA in the initial population was assigned based on the distribution observed in the Population Data BC database.

Effect of BMI on OA incidence

Relative risks (hazard ratios) for the effect of BMI on OA incidence was derived by analyzing longitudinal data from multiple cycles (1994/96/98/00/02) of the NPHS [16] (Table A1-4). Both OA and BMI were self-reported. We applied a survival regression model, adjusting for age. For the underweight category, because the sample size in the NPHS was too small, we obtained the relative risks from the CCHS (cross-sectional data) and adjusted for cross-sectional bias by applying as a multiplier the mean ratio of the relative risks for the other BMI categories from the NPHS to the relative risks from the CCHS.

Medication use

We computed a drug use table, which contains stratified estimated probabilities of medication use (each of four medication categories) according to age group, sex, OA stage, and level of pain (Table A1-5). The drugs modeled, Drug(i) (i=1 to 4) were: 1) Acetaminophen (paracetamol); 2) Non-steroidal anti-inflammatory drugs (NSAIDs); 3) COX-2 inhibitors (coxibs) and 4) Opioids. The level of pain was obtained from the pain domain of the Health Utilities Index Mark 3 (HUI3), a well-established measure of health utility, which consists of 8 domains (vision, hearing, speech, mobility, dexterity, cognition, emotion, and pain/discomfort). For details of modeling HUI3, please see the Section “HUI3 domain-specific model”. Modelling drug use was carried out in the following steps:

Step 1. Using NPHS data, we modeled each Drug(i) as a function HUI3 Pain domain + age + sex + SROA, where age is continuous, and SROA is self-reported OA (0/1). Analysis dataset was the NPHS combined cycles, age 12+ only. We obtained odds ratios (ORs) estimating adjusted effect of pain levels on drug use.

Step 2a. Using NPHS data, we estimated Canadian proportion using Drug(i), separately for each cycle (2000, 2002, 2004, 2006, 2008, 2010), age 12+.

Step 2b. We fit models to estimate Canadian use in 2012 of each Drug(i) from the 6 data points obtained in Step 2a. We used linear models with dependent variable percent use, including the following independent variables: Acetaminophen: $\log_{10}$(year-1990); NSAIDS: $\log_{10}$(year-1990); Coxibs: (year-1990)2 + (year-1990) and Opioids: $\log_{10}$(year-1990).

Step 3a. Using NPHS data, we estimated British Columbia (BC) proportion using Drug(i), separately for each cycle (2000, 2002, 2004, 2006, 2008, 2010), age 12+.

Step 3b. We fit models to estimate BC use in 2012 of each Drug(i) from the 6 data points obtained in Step 3a. We used linear models with dependent variable percent use, using the same independent variables as before.

Step 4. Using public use CCHS 2001 cycle, we estimated Canadian proportions in each HUI3 Pain level according to age group (12-49, 50-59, 60-69, 70-79, 80+), sex and SROA.

Step 5. Using BC administrative data, we estimated proportion using Drug(i) on a given day in 2012, according to age group (12-49, 50-59, 60-69, 70-79, 80+), sex and OA stage (NoOA, OA<5 year, OA>=5 years, JR<5 years, JR>=5 years).

Step 6. Using BC administrative data (12+), we estimated the aggregate proportion using Drug(i) on a given day in 2012.

Step 7. We estimated drug use ratios to convert probability estimates for Drug(i) from administrative to self-report metric, as follows. Let SRprob_Drug(i) = P(Drug(i)) in 2012 estimated in Step 2b on self-report aggregate Canadian data, and ADMINprob_Drug(i) = P(Drug(i)) in 2012 estimated in Step 6 on administrative aggregate BC data. Then Ratio_Drug(i) = logit (SRprob_Drug(i)) - logit (ADMINprob_Drug(i)) (note, this is a difference in logits, not a ratio of logits).

Step 8. For each Drug(i), we estimated Canadian_Proportion_Using_Drug(i) according to age group, sex and OA stage, per 1/(1+exp(-1*(Ratio_Drug(i)(Step 8)+logit(BCAdmin_Proportion_Using_Drug(i)(Step 5)))).

Step 9. We applied titration combining estimates from Steps 1, 4 and 9, to solve for P(Drug(i) |HUIPain=1) such that Sum[j=1 to 5](P(Drug(i)|HUIPain=j)*CanadianProportion(HUIPain=j)= Canadian_Proportion_Using_Drug(i). Note, we only solved for the one unknown, since from P(Drug(i) |HUIPain=1) and the ORs, all the other Pj=P(Drug(i) |HUIPain=j) are determined per Pj=(ORj*P1/(1-P1))/(1+ORj*P1/(1-P1)).

Side effects of medication

We considered four side-effects (complications) associated with the use of NSAIDs, coxibs and opioids in OA: serious GI complications (ulcer with bleeding or perforation); dyspepsia; cardiovascular disease (CVD, except stroke); and stroke. In addition, we included lethal overdose as a side effect of opioids. We assumed no side effects from acetaminophen. The parameters describing the risk of side effects were derived from the literature [26-36]. However, we used different types of parameters for different side effects. For CVD and stroke, we obtained baseline incidence rates of these conditions in Canada from 2001 to 2017 (assumed to be constant thereafter) by age, sex and year from the Global Burden of Disease Study (GBD 2017) (Tables A1-6 and A1-7) and multiplied them by the relative risks (RRs) associated with the use of each medication, derived from the literature (Table A1-8). These RRs were

assumed to be constant across age and sex strata and similar for all three classes of drugs [32,34). For GI complications, we obtained excess rates of ulcer and dyspepsia among patients taking medication from the literature (Table A1-8). We assumed those rates to vary by age (<70 vs. 70+ years). For opioid overdose, we used an estimate of the total number of deaths in Canada due to prescription opioids, excluding deaths due to illicit drugs (Table A1-8) and derived the probability of lethal overdose among patients with OA. Probabilities of death due to CVD and stroke by age, sex, and year were obtained from GBD 2017 as mortality/incidence ratios (Tables A1-9 and A1-10), and the risk of death from bleeding or perforated ulcer was obtained from the literature [43,44] (Table A1-11).

Impact of medication on HUI3

We assumed pain was the only HUI3 domain affected directly by medication (please see the Section on modeling HUI3). Other domains could have been affected indirectly, because of their relationship with pain. The effects of each medication type on pain was obtained from meta-analyses of published randomized trials (Table A1-12). The effect was expressed as change in pain relative to baseline, in a before-after comparison (rather than against placebo). This was done to better reflect the real-life effect of treatment, which includes the placebo effect. Each simulated subject was randomly assigned a probability of medication being effective. It should be noted that in our model pain was one of the factors determining the use of medication (Table A1-5) and the use of medication affected the level of pain. To avoid a circular relationship, we modeled two types of pain for each simulated subject taking medication, latent or counterfactual pain that the patient would have experienced without medication and actual pain (on medication). The probability of taking pain medication was determined by the former type of pain, whereas effect on HUI3 was determined by the latter. Rather than modeling the level of pain reduction in each simulated individual, we used the pain reduction parameter to simulate the proportion of users who get 100% reduction in pain (which is equivalent).

Frequency of joint replacement surgery (JRS)

We used administrative data from BC (Pop Data BC – Medical Services Plan Database) to derive baseline JRS rates. We defined primary and revision JRS in hospital data as a Canadian Classification of Diagnostic, Therapeutic, and Surgical Procedures (CCP) procedure code 935 or 934.1, or a Canadian Classification of Health Interventions (CCI) code 1VA53LAPN or 1VA53PNPN or 1VG53. We excluded cases if concurrently there was a diagnosis in the hospital record of the following ICD9 codes: 800-999, E800-E869, E880-E928, E950-E999, 140-208, 235-239, except codes 996.4, 996.6, 996.7, or T84, or the following ICD10 codes: S00-S99, V01-V99, W00-W99, X093-X99, C00-C97 and D37-D48. We defined joint replacement surgery as revision if concurrently there was a diagnosis in that hospital record with codes 996.4, 996.6, 996.7, or T84, otherwise, it was defined as primary. Baseline rates of primary and revision JRS by age, sex and time were obtained from a Poisson regression model with predictors age group, sex, and $\log_{10}$ (year-2000). The latter term was needed to accommodate a time trend, and necessitated that the model not be applied until the year 2001 at the earliest (current starting year for MSM-OA). Subjects could have up to 4 primary surgeries and any number of revision surgeries over lifetime. These models were only applied once the subject had OA. The parameters (model coefficients) are in Table A1-13.

To model JRS as a function of HUI3 domains, we carried out a case-control study in which cases were 220 patients with OA scheduled to undergo hip/knee replacement at Vancouver General Hospital between April 2000 and May 2005, and controls were age- and sex-matched (3:1) participants in the 2000/2001 Canadian Community Health Survey who reported being diagnosed with OA [38]. The predictor variables were 8 HUI3 domains (vision, hearing, speech, dexterity, ambulation, pain, cognition, and emotion). HUI3 was measured prior to surgery in the cases and as part of the survey in the controls. We used conditional logistic regression to model the association of JRS with each of the dichotomized (for pain there were 3 categories) HUI3 domains (Table A1-14). The baseline JRS rates by age and year for males and females are shown in Table A1-15 (primary) and Table A1-16 (revisions).

We fit the following model to predict a run-in time correction factor (the ratio of asymptotically estimated OA prevalence over the estimated prevalence at a given run-in time): OA_prev ~ years + years^2 + $\log_{10}$(years). The plots in Figure 1 show that the model fits the published numbers very well, and predictions asymptotically approach a sensible level. The second plot illustrates our approach to subsequently level off the estimated asymptotic prevalence rather than let it begin to drop when too far beyond the data range.

Since our data begin in 1990, we inflated the OA person-time for each subject within each calendar year they contributed to, by the factor prev(20) / (prev(min(20,max(year,1991)-1990)), where prev(t) is to be read off the right plot (Figure 1). We then fit Poisson models for primary and revision surgery separately, as ln(E(count)) ~ age group + sex + $\log_{10}$(year-2000), with an offset to put estimated regression coefficients in the per 1000 person-year scale.

Mortality post-surgery

The risk of death post JRS relative to age/sex-specific general population risk was assumed to be 1.80 during the first 90 days post-JRS based on data from the literature [45-47]. After 90 days the risk of death was assumed to return to the general population risk.

HUI3 domain-specific model

We modeled each HUI3 domain (attribute) separately. To this end, we used NPHS (longitudinal data, 1994-2012) for model development. The statistical method was ordinal logistic regression with proportional odds. The HUI3 domains were initially measured on a 5 or 6-point ordinal scale; however, all domains except pain were collapsed due to reporting limitations of public-use CCHS and/or for model stability, as follows: Vision: (1, 2, 3): 1, 2, 3+4+5+6; Hearing: (1, 2, 3): 1, 2+3, 4+5+6; Speech: (1, 2): 1, 2+3+4+5; Mobility: (1, 2, 3, 4): 1, 2, 3+4, 5+6; Dexterity: (1, 2): 1, 2+3+4+5+6; Emotion: (1, 2, 3, 4): 1, 2, 3, 4+5; Cognition: (1, 2, 3, 4, 5): 1, 2, 3, 4, 5+6; Pain: (1, 2, 3, 4, 5): 1, 2, 3, 4, 5.

For each domain, predictors included previous values of the domain (2 years back), calendar time, demographics (age, sex, education, and income), smoking, BMI, OA, and selected comorbidities (diabetes, hypertension, CVD). Additional predictors included other selected concurrent HUI3 domains, according to a conceptual, hierarchical model. Specifically, domains were updated one at a time, each model including concurrent values of the already-updated domains, as described below. In separate models, HUI3 domains could also be influenced by medical and surgical treatment of OA and side effects of treatment, as discussed previously.

We applied domain-specific proportional odds (PO) models to predict each domain of HUI, applying models and updating domains in the following order: Vision, Hearing, Speech, Pain, Cognition, Dexterity, Mobility, Emotion. The order was important because models were nested. The vision model did not include any other domains, the hearing model included vision as a predictor, the speech model contained vision and hearing as predictors, etc., until finally the emotion model included all the other domains as predictors. After each domain-specific model was run, that domain was updated before applying the next model.

To update a given component, the cumulative logit model was applied. To this end, we calculated the linear predictor etai=X*beta for each level of the domain except level 1. The ith intercept was selected for the ith level's calculation. The intercept selected depended on the level being calculated, this was calculated once with each intercept. Then a cumulative probability was calculated as P(level≥i)=1/(1+exp(-etai)). This was repeated for each intercept from i=2 to k (where there are k levels in the domain). We used subtraction to calculate point probabilities from the cumulative probabilities as follows: P(level=j)=P(level≥j)-P(level≥j+1), where P(level≥1)=1. This produced a probability mass function (PMF) from which POHEM randomly selected a level for the given HUI3 domain to assign to the subject. When calculating a linear predictor, we applied a multiplier to each coefficient of either 0 or 1 (for

categorical variables, where a 1 is for the applicable category), or the variable's value for a continuous variable.

After all domains were updated, we re-calculated the aggregate HUI3 from the individual components using modified HUI3 coefficients derived as population-weighted averages of the coefficients for the un-collapsed categories that are available at http://www.healthutilities.com/hui3.htm. The population counts used in the weighting were obtained from the master CCHS file. The coefficients were calculated as follows:

Vision:

coefvis{1}=1.00;

coefvis{2}=0.98;

coefvis{3}=(0.89*132.834+0.84*158.162+0.75*83.976+0.61*19.971)/(132.834+158.162+83.976+19.971)
;

Hearing:

coefher{1}=1.00;

coefher{2}=(0.95*470.276+0.89*181.772)/(470.276+181.772);

coefher{3}=(0.80*221.945+0.74*38.196+0.61*39.907)/(221.945+38.196+39.907);

Speech:

coefspe{1}=1.00;

coefspe{2}=(0.94*110.670+0.89*19.314+0.81*25.545+0.68*14.088)/(110.670+19.314+25.545+14.088);

Mobility:

coefmob{1}=1.00;

coefmob{2}=0.93;

coefmob{3}=(0.86*429.728+0.73*32.725)/(429.728+32.725);

coefmob{4}=(0.65*198.938+0.58*85.353)/(198.938+85.353);

Dexterity:

coefdex{1}=1.00;

coefdex{2}=(0.95*105.380+0.88*5.111+0.76*52.431+0.65*35.058+0.56*15.119)/(105.380+5.111+52.431+35.058+15.119);

Emotion:

coefemo{1}=1.00;

coefemo{2}=0.95;

coefemo{3}=0.85;

coefemo{4}=(0.64*186.387+0.46*46.601)/(186.387+46.601);

Cognition:

coefcog{1}=1.00;

coefcog{2}=0.92;

coefcog{3}=0.95;

coefcog{4}=0.83;

coefcog{5}=(0.60*475.860+0.42*46.225)/(475.860+46.225);

Pain or discomfort:

coefpad{1}=1.00;

coefpad{2}=0.96;

coefpad{3}=0.90;

coefpad{4}=0.77;

coefpad{5}=0.55;

HUI3 aggregate was then calculated as: HUI3 =1.371*(b1*b2*b3*b4*b5*b6*b7*b8)-0.371, where b1-b8 are the modified coefficients for the 8 domains.

Model coefficients for the side effects of medication (stroke and ulcer/bleeding) for the HUI3 domains were obtained from the CCHS 2001 data. The effects of the side effects were adjusted for the same variables as the HUI3 domain models as follows:

1. Ordinal logistic models (proportional odds models) were fit regressing each collapsed ordinal HUI3 domain on stroke and ulcer in the same model plus the same variables as in the HUI3 model (Table A1-17) except for calendar year and the previous values of the given domain (since CCHS is cross-sectional). One other difference is that regular smoking in the past year was not available in CCHS 2001 public use. Instead we controlled for smoking not/occasional/regular per variable in CCHS.
2. Regression coefficients and SEs were added for non-ulcer dyspepsia, defined as 0.4 times those of ulcer, after consulting literature on the effects of the two side effects on various HRQOL measures [41-43].
3. We compared the regression coefficients for CVD, osteoarthritis, diabetes, and high blood pressure not adjusted for year and not including previous values of the domain vs. models

adjusted for log base 10 of year and either one or two 2-year cycles back values of the HUI3 outcome domain. For each domain, the median of the eight ratios thus obtained was taken as the domain ratio. This domain ratio was multiplied against the regression coefficients and SEs for stroke, ulcer and non-ulcer dyspepsia in the new no-previous-year CCHS models. The purpose of this step was to reflect in the estimates the effects of year and autoregressive variables in the model on the odds ratios.

The additional side effects were treated as 0s when applying the expanded HUI3 domain models, until such time as they appeared as side effects of drugs. The coefficients are shown in Table A1-17.

HUI3 post JRS

Data for modeling the HUI3 domains post-surgery were obtained from a cohort of patients undergoing JRS at the Vancouver General Hospital [37]. The domains post-JRS were updated using the same approach as described above for the general HUI3 model; however, the model included only age, sex, pre-JRS value of the HUI3 domain being modeled and either pre- or post-JRS values of each of the other domains, according to our hierarchical model. That is, vision included only pre-JRS values of the other domains, hearing included pre-JRS values of all other domains except vision and post-JRS value for vision, and so on. Emotion included post-JRS values of all other domains (Table A1-18). This model was applied once post-JRS, using data from a cohort study of patients undergoing JRS, obtained on average a year post-surgery. After that, HUI3 domains continued to be updated using the general HUI3 model (Table A1-17) that was used prior to JRS; however, prior levels of the domains in this model were those observed after surgery.

Table A1-1: Baseline death rates per 1000 in MSM-OA by sex for selected ages and years, including projected rates.

Age	2001	2006	2011	2016	2021	2026	2031	2036	2041
Male									
20	0.585	0.524	0.491	0.469	0.446	0.425	0.425	0.425	0.425
30	0.728	0.696	0.661	0.630	0.599	0.570	0.570	0.570	0.570
40	0.840	0.799	0.761	0.724	0.689	0.656	0.656	0.656	0.656
50	2.066	1.966	1.871	1.780	1.694	1.612	1.612	1.612	1.612
60	5.727	5.451	5.188	4.937	4.699	4.472	4.472	4.472	4.472
70	14.427	13.731	13.068	12.437	11.837	11.265	11.265	11.265	11.265
80	34.414	32.753	31.172	29.667	28.235	26.872	26.872	26.872	26.872
90	81.330	77.404	73.668	70.112	66.728	63.507	63.507	63.507	63.507
Female									
20	0.262	0.252	0.243	0.238	0.232	0.227	0.227	0.227	0.227
30	0.347	0.339	0.330	0.322	0.314	0.306	0.306	0.306	0.306
40	0.693	0.676	0.659	0.643	0.627	0.611	0.611	0.611	0.611
50	1.783	1.739	1.696	1.654	1.613	1.573	1.573	1.573	1.573
60	4.695	4.579	4.466	4.356	4.248	4.143	4.143	4.143	4.143
70	12.284	11.981	11.686	11.397	11.116	10.842	10.842	10.842	10.842
80	31.027	30.261	29.515	28.787	28.077	27.385	27.385	27.385	27.385
90	73.826	72.005	70.229	68.497	66.807	65.160	65.160	65.160	65.160

Footnote: Death rates are shown here for illustration purposes. In the actual simulation, we used data for each year of age and each calendar year.

Table A1-2: Coefficients for eight BMI models in MSM-OA.

	# prior BMI values = 1		# prior BMI values = 2		# prior BMI values = 3		# prior BMI values = 4	
	Female	Male	Female	Male	Female	Male	Female	Male
Intercept	0.15402	0.18426	0.05041	0.09689	0.01621	0.05386	-0.01122	0.07594
Age group								
20-24	0.03730	0.03321	0.04795	0.03725	0.05438	0.04186	0.06100	0.06031
25-29	0.03730	0.03321	0.04795	0.03725	0.05438	0.04186	0.06100	0.06031
30-34	0.02743	0.02545	0.03470	0.02815	0.04069	0.03184	0.04064	0.03719
35-39	0.02999	0.02356	0.03581	0.02378	0.03973	0.02671	0.04241	0.02669
40-44	0.02729	0.02702	0.03469	0.02643	0.03885	0.02914	0.04161	0.03346
45-49	0.02711	0.02369	0.03362	0.02411	0.03673	0.02667	0.03758	0.02827
50-54	0.02924	0.02284	0.03249	0.02294	0.03577	0.02467	0.03698	0.02701
55-59	0.02831	0.02309	0.03124	0.02002	0.03362	0.02142	0.03246	0.02454
60-64	0.02988	0.02146	0.03332	0.02007	0.03501	0.02051	0.03240	0.02381
65-69	0.02144	0.01093	0.02263	0.00932	0.02413	0.01058	0.02350	0.01275
70-74	0.01649	0.01177	0.01645	0.00804	0.01806	0.00774	0.01813	0.01138
75-79	0.01122	0.00919	0.01255	0.00845	0.01309	0.00700	0.01193	0.01161
80+ (ref)	0.00000	0.00000	0.00000	0.00000	0.00000	0.00000	0.00000	0.00000
Education								
Grp. 1	0.00103	-0.00049	-0.00081	-0.00006	-0.00087	0.00091	-0.00332	-0.00049
Grp. 2	0.00130	0.00133	0.00039	0.00080	0.00080	0.00112	0.00140	0.00021
Grp. 3	0.00187	0.00007	0.00103	0.00042	0.00156	0.00133	0.00291	0.00021
Grp. 4 (ref)	0.00000	0.00000	0.00000	0.00000	0.00000	0.00000	0.00000	0.00000
Income quartiles								
Q1	0.00458	0.00027	0.00323	0.00142	0.00238	0.00159	0.00327	-0.00209
Q2	0.00460	-0.00109	0.00436	-0.00098	0.00365	-0.00140	0.00100	-0.00055
Q3	0.00396	-0.00003	0.00337	0.00040	0.00328	0.00037	0.00292	-0.00276
Q4 (ref)	0.00000	0.00000	0.00000	0.00000	0.00000	0.00000	0.00000	0.00000
Province								
Atlantic	0.0052	0.0027	0.0040	0.0014	0.0028	0.0012	0.0054	-0.0008
Quebec	0.0028	-0.0002	0.0044	0.0016	0.0041	0.0026	0.0059	0.0000
Ontario	0.0018	0.0021	0.0025	0.0014	0.0019	0.0015	0.0046	0.0006
Prairies	0.0035	0.0005	0.0025	0.0003	0.0015	0.0008	0.0023	-0.0020
BC (ref)	0.0000	0.0000	0.0000	0.0000	0.0000	0.0000	0.0000	0.0000
Prior BMI								
2 years	0.94474	0.93899	0.68029	0.65988	0.6407	0.61521	0.61823	0.56538
4 years	0	0	0.29633	0.30661	0.22277	0.19815	0.21434	0.20767
6 years	0	0	0	0	0.12364	0.16618	0.07103	0.14224
8 years	0	0	0	0	0	0	0.09153	0.057682
Std. error	0.07005	0.05646	0.06676	0.05337	0.06618	0.05272	0.06645	0.05242

Education categories: Group 1 = <Secondary; Group 2 = Secondary graduate; Group 3 = Some post-secondary; Group 4 = Post-secondary graduate

Table A1-3: Baseline OA incidence rates per 1000 person-years by sex for selected ages

Age	Female	Male
20	0.580	0.541
25	0.704	0.765
30	1.214	1.434
35	2.029	2.178
40	3.106	3.513
45	5.342	5.531
50	8.239	6.802
55	12.726	10.091
60	16.381	13.132
65	19.256	15.197
70	23.154	18.273
75	26.894	20.270
80	29.921	23.810
85	28.405	24.438
90	31.114	28.142
95	30.253	25.540

Incidence rates for selected ages are shown for illustration purposes. In the simulation, we used rates for each single year of age.

Table A1-4: Relative risks (hazard ratios) for the effect of BMI on OA incidence.

	Underweight BMI<18.5	Normal weight 18.5≤BMI<25	Overweight 25≤BMI<30	Obese BMI≥30
Females	0.33	1.0	1.76	2.03
Males	0.01	1.0	1.07	1.69

The relative risk were estimated using a survival regression model.

Table A1-5: Probabilities of taking each medication according to sex, age, OA stage, and HUI3 level of pain.

Medication	Sex	Age	OA stage	Pain 1	Pain 2	Pain 3	Pain 4	Pain 5
Acetaminophen	Male	12-49	No OA	0.0153	0.0240	0.0294	0.0319	0.0347
Acetaminophen	Male	50-59	No OA	0.0319	0.0496	0.0604	0.0654	0.0709
Acetaminophen	Male	60-69	No OA	0.0356	0.0554	0.0672	0.0728	0.0788
Acetaminophen	Male	70-79	No OA	0.0631	0.0966	0.1162	0.1252	0.1350
Acetaminophen	Male	80+	No OA	0.1941	0.2765	0.3197	0.3385	0.3581
Acetaminophen	Female	12-49	No OA	0.0225	0.0353	0.0430	0.0467	0.0507
Acetaminophen	Female	50-59	No OA	0.0405	0.0628	0.0761	0.0824	0.0891
Acetaminophen	Female	60-69	No OA	0.0424	0.0657	0.0796	0.0860	0.0931
Acetaminophen	Female	70-79	No OA	0.0748	0.1137	0.1362	0.1466	0.1577
Acetaminophen	Female	80+	No OA	0.3019	0.4069	0.4577	0.4789	0.5004
Acetaminophen	Male	12-49	OA<5	0.0732	0.1114	0.1336	0.1437	0.1547
Acetaminophen	Male	12-49	OA≥5	0.0678	0.1035	0.1243	0.1339	0.1442
Acetaminophen	Male	12-49	JR<5	0.1154	0.1715	0.2029	0.2171	0.2321
Acetaminophen	Male	12-49	JR≥5	0.1882	0.2688	0.3114	0.3300	0.3493
Acetaminophen	Male	50-59	OA<5	0.0745	0.1133	0.1358	0.1461	0.1572
Acetaminophen	Male	50-59	OA≥5	0.0992	0.1487	0.1768	0.1896	0.2032
Acetaminophen	Male	50-59	JR<5	0.1020	0.1527	0.1814	0.1944	0.2082
Acetaminophen	Male	50-59	JR≥5	0.1674	0.2419	0.2818	0.2994	0.3178
Acetaminophen	Male	60-69	OA<5	0.0935	0.1407	0.1676	0.1798	0.1929
Acetaminophen	Male	60-69	OA≥5	0.0959	0.1440	0.1714	0.1838	0.1971
Acetaminophen	Male	60-69	JR<5	0.1136	0.1689	0.2000	0.2140	0.2288
Acetaminophen	Male	60-69	JR≥5	0.0953	0.1432	0.1705	0.1829	0.1962
Acetaminophen	Male	70-79	OA<5	0.1359	0.1997	0.2348	0.2505	0.2671
Acetaminophen	Male	70-79	OA≥5	0.1132	0.1684	0.1993	0.2133	0.2281
Acetaminophen	Male	70-79	JR<5	0.0703	0.1071	0.1285	0.1384	0.1490
Acetaminophen	Male	70-79	JR≥5	0.1080	0.1611	0.1911	0.2046	0.2190
Acetaminophen	Male	80+	OA<5	0.3393	0.4489	0.5004	0.5217	0.5432
Acetaminophen	Male	80+	OA≥5	0.3275	0.4359	0.4872	0.5086	0.5301
Acetaminophen	Male	80+	JR<5	0.1629	0.2359	0.2752	0.2925	0.3107
Acetaminophen	Male	80+	JR≥5	0.2998	0.4045	0.4552	0.4764	0.4979
Acetaminophen	Female	12-49	OA<5	0.1348	0.1982	0.2332	0.2488	0.2652
Acetaminophen	Female	12-49	OA≥5	0.1233	0.1824	0.2152	0.2300	0.2456
Acetaminophen	Female	12-49	JR<5	0.2028	0.2875	0.3317	0.3508	0.3707
Acetaminophen	Female	12-49	JR≥5	0.0381	0.0591	0.0717	0.0776	0.0840
Acetaminophen	Female	50-59	OA<5	0.0987	0.1480	0.1761	0.1888	0.2023
Acetaminophen	Female	50-59	OA≥5	0.1140	0.1695	0.2006	0.2146	0.2295
Acetaminophen	Female	50-59	JR<5	0.1192	0.1767	0.2088	0.2233	0.2386
Acetaminophen	Female	50-59	JR≥5	0.0467	0.0721	0.0873	0.0943	0.1019
Acetaminophen	Female	60-69	OA<5	0.0979	0.1468	0.1747	0.1873	0.2008
Acetaminophen	Female	60-69	OA≥5	0.1102	0.1642	0.1946	0.2083	0.2229
Acetaminophen	Female	60-69	JR<5	0.1261	0.1863	0.2197	0.2347	0.2505

Acetaminophen	Female	60-69	JR≥5	0.1668	0.2410	0.2809	0.2984	0.3168
Acetaminophen	Female	70-79	OA<5	0.1374	0.2017	0.2371	0.2529	0.2695
Acetaminophen	Female	70-79	OA≥5	0.1462	0.2136	0.2504	0.2667	0.2839
Acetaminophen	Female	70-79	JR<5	0.1478	0.2158	0.2529	0.2693	0.2866
Acetaminophen	Female	70-79	JR≥5	0.1562	0.2271	0.2654	0.2823	0.3001
Acetaminophen	Female	80+	OA<5	0.4594	0.5741	0.6237	0.6435	0.6630
Acetaminophen	Female	80+	OA≥5	0.4238	0.5384	0.5893	0.6097	0.6300
Acetaminophen	Female	80+	JR<5	0.2788	0.3801	0.4299	0.4510	0.4724
Acetaminophen	Female	80+	JR≥5	0.3948	0.5085	0.5599	0.5808	0.6017
NSAIDs	Male	12-49	No OA	0.0618	0.0878	0.1186	0.1153	0.1132
NSAIDs	Male	50-59	No OA	0.1362	0.1873	0.2438	0.2378	0.2342
NSAIDs	Male	60-69	No OA	0.1623	0.2206	0.2837	0.2771	0.2730
NSAIDs	Male	70-79	No OA	0.2007	0.2685	0.3392	0.3319	0.3274
NSAIDs	Male	80+	No OA	0.3231	0.4110	0.4939	0.4858	0.4807
NSAIDs	Female	12-49	No OA	0.0717	0.1014	0.1364	0.1326	0.1302
NSAIDs	Female	50-59	No OA	0.1165	0.1617	0.2124	0.2070	0.2037
NSAIDs	Female	60-69	No OA	0.1241	0.1716	0.2246	0.2189	0.2155
NSAIDs	Female	70-79	No OA	0.1704	0.2310	0.2958	0.2890	0.2849
NSAIDs	Female	80+	No OA	0.3265	0.4147	0.4978	0.4896	0.4845
NSAIDs	Male	12-49	OA<5	0.2755	0.3572	0.4373	0.4293	0.4244
NSAIDs	Male	12-49	OA≥5	0.2245	0.2973	0.3717	0.3641	0.3595
NSAIDs	Male	12-49	JR<5	0.2601	0.3394	0.4181	0.4102	0.4053
NSAIDs	Male	12-49	JR≥5	0.3203	0.4079	0.4907	0.4825	0.4775
NSAIDs	Male	50-59	OA<5	0.3195	0.4070	0.4898	0.4816	0.4766
NSAIDs	Male	50-59	OA≥5	0.3087	0.3950	0.4773	0.4691	0.4641
NSAIDs	Male	50-59	JR<5	0.3334	0.4224	0.5056	0.4974	0.4924
NSAIDs	Male	50-59	JR≥5	0.2886	0.3723	0.4534	0.4453	0.4403
NSAIDs	Male	60-69	OA<5	0.3339	0.4229	0.5061	0.4979	0.4929
NSAIDs	Male	60-69	OA≥5	0.3219	0.4096	0.4925	0.4843	0.4793
NSAIDs	Male	60-69	JR<5	0.3543	0.4451	0.5287	0.5205	0.5155
NSAIDs	Male	60-69	JR≥5	0.3392	0.4286	0.5120	0.5038	0.4988
NSAIDs	Male	70-79	OA<5	0.3119	0.3985	0.4810	0.4728	0.4678
NSAIDs	Male	70-79	OA≥5	0.3137	0.4005	0.4831	0.4749	0.4699
NSAIDs	Male	70-79	JR<5	0.3408	0.4305	0.5139	0.5057	0.5007
NSAIDs	Male	70-79	JR≥5	0.2934	0.3777	0.4591	0.4510	0.4460
NSAIDs	Male	80+	OA<5	0.3885	0.4815	0.5650	0.5569	0.5519
NSAIDs	Male	80+	OA≥5	0.4390	0.5335	0.6153	0.6076	0.6027
NSAIDs	Male	80+	JR<5	0.3523	0.4429	0.5265	0.5184	0.5133
NSAIDs	Male	80+	JR≥5	0.4131	0.5071	0.5900	0.5821	0.5772
NSAIDs	Female	12-49	OA<5	0.3082	0.3944	0.4767	0.4685	0.4635
NSAIDs	Female	12-49	OA≥5	0.2711	0.3522	0.4319	0.4239	0.4190
NSAIDs	Female	12-49	JR<5	0.3881	0.4811	0.5646	0.5566	0.5516
NSAIDs	Female	12-49	JR≥5	0.3110	0.3975	0.4799	0.4718	0.4667
NSAIDs	Female	50-59	OA<5	0.3038	0.3895	0.4715	0.4634	0.4584
NSAIDs	Female	50-59	OA≥5	0.3045	0.3902	0.4723	0.4641	0.4591
NSAIDs	Female	50-59	JR<5	0.3893	0.4823	0.5658	0.5578	0.5528

NSAIDs	Female	50-59	JR≥5	0.3598	0.4510	0.5346	0.5265	0.5215
NSAIDs	Female	60-69	OA<5	0.2940	0.3783	0.4598	0.4517	0.4467
NSAIDs	Female	60-69	OA≥5	0.2959	0.3805	0.4621	0.4540	0.4490
NSAIDs	Female	60-69	JR<5	0.3707	0.4627	0.5464	0.5383	0.5333
NSAIDs	Female	60-69	JR≥5	0.3995	0.4930	0.5763	0.5683	0.5633
NSAIDs	Female	70-79	OA<5	0.2703	0.3513	0.4310	0.4230	0.4180
NSAIDs	Female	70-79	OA≥5	0.2910	0.3750	0.4562	0.4482	0.4432
NSAIDs	Female	70-79	JR<5	0.3070	0.3930	0.4752	0.4671	0.4621
NSAIDs	Female	70-79	JR≥5	0.3513	0.4418	0.5254	0.5172	0.5122
NSAIDs	Female	80+	OA<5	0.3751	0.4673	0.5510	0.5429	0.5379
NSAIDs	Female	80+	OA≥5	0.3936	0.4869	0.5703	0.5622	0.5573
NSAIDs	Female	80+	JR<5	0.3410	0.4307	0.5141	0.5059	0.5009
NSAIDs	Female	80+	JR≥5	0.3880	0.4810	0.5645	0.5565	0.5515
Coxibs	Male	12-49	No OA	0.0017	0.0040	0.0092	0.0076	0.0104
Coxibs	Male	50-59	No OA	0.0048	0.0112	0.0253	0.0211	0.0288
Coxibs	Male	60-69	No OA	0.0056	0.0130	0.0293	0.0244	0.0332
Coxibs	Male	70-79	No OA	0.0047	0.0109	0.0246	0.0204	0.0279
Coxibs	Male	80+	No OA	0.0032	0.0076	0.0172	0.0143	0.0195
Coxibs	Female	12-49	No OA	0.0019	0.0044	0.0100	0.0083	0.0114
Coxibs	Female	50-59	No OA	0.0051	0.0119	0.0268	0.0223	0.0304
Coxibs	Female	60-69	No OA	0.0061	0.0141	0.0317	0.0264	0.0359
Coxibs	Female	70-79	No OA	0.0053	0.0124	0.0280	0.0233	0.0317
Coxibs	Female	80+	No OA	0.0037	0.0087	0.0196	0.0163	0.0223
Coxibs	Male	12-49	OA<5	0.0094	0.0217	0.0483	0.0403	0.0547
Coxibs	Male	12-49	OA≥5	0.0068	0.0158	0.0354	0.0295	0.0401
Coxibs	Male	12-49	JR<5	0.0137	0.0314	0.0691	0.0580	0.0780
Coxibs	Male	12-49	JR≥5	0.0075	0.0173	0.0388	0.0323	0.0439
Coxibs	Male	50-59	OA<5	0.0162	0.0371	0.0812	0.0682	0.0914
Coxibs	Male	50-59	OA≥5	0.0170	0.0389	0.0848	0.0713	0.0955
Coxibs	Male	50-59	JR<5	0.0212	0.0483	0.1041	0.0878	0.1169
Coxibs	Male	50-59	JR≥5	0.0157	0.0361	0.0790	0.0663	0.0890
Coxibs	Male	60-69	OA<5	0.0219	0.0498	0.1072	0.0904	0.1203
Coxibs	Male	60-69	OA≥5	0.0197	0.0450	0.0974	0.0821	0.1095
Coxibs	Male	60-69	JR<5	0.0373	0.0832	0.1721	0.1468	0.1914
Coxibs	Male	60-69	JR≥5	0.0310	0.0698	0.1466	0.1246	0.1637
Coxibs	Male	70-79	OA<5	0.0164	0.0376	0.0821	0.0690	0.0925
Coxibs	Male	70-79	OA≥5	0.0101	0.0233	0.0519	0.0434	0.0587
Coxibs	Male	70-79	JR<5	0.0176	0.0404	0.0879	0.0739	0.0988
Coxibs	Male	70-79	JR≥5	0.0164	0.0376	0.0820	0.0689	0.0924
Coxibs	Male	80+	OA<5	0.0100	0.0230	0.0512	0.0428	0.0579
Coxibs	Male	80+	OA≥5	0.0098	0.0226	0.0504	0.0421	0.0570
Coxibs	Male	80+	JR<5	0.0065	0.0150	0.0337	0.0281	0.0383
Coxibs	Male	80+	JR≥5	0.0166	0.0380	0.0830	0.0697	0.0934
Coxibs	Female	12-49	OA<5	0.0171	0.0391	0.0852	0.0716	0.0959
Coxibs	Female	12-49	OA≥5	0.0069	0.0160	0.0358	0.0299	0.0406
Coxibs	Female	12-49	JR<5	0.0197	0.0450	0.0973	0.0820	0.1093

Coxibs	Female	12-49	JR≥5	0.0122	0.0280	0.0619	0.0518	0.0699
Coxibs	Female	50-59	OA<5	0.0215	0.0490	0.1056	0.0891	0.1186
Coxibs	Female	50-59	OA≥5	0.0193	0.0441	0.0955	0.0804	0.1074
Coxibs	Female	50-59	JR<5	0.0349	0.0780	0.1623	0.1383	0.1808
Coxibs	Female	50-59	JR≥5	0.0443	0.0980	0.1992	0.1708	0.2208
Coxibs	Female	60-69	OA<5	0.0198	0.0451	0.0976	0.0822	0.1097
Coxibs	Female	60-69	OA≥5	0.0202	0.0461	0.0996	0.0839	0.1119
Coxibs	Female	60-69	JR<5	0.0520	0.1139	0.2274	0.1960	0.2511
Coxibs	Female	60-69	JR≥5	0.0393	0.0874	0.1800	0.1538	0.2000
Coxibs	Female	70-79	OA<5	0.0112	0.0258	0.0572	0.0478	0.0646
Coxibs	Female	70-79	OA≥5	0.0164	0.0376	0.0821	0.0689	0.0924
Coxibs	Female	70-79	JR<5	0.0216	0.0492	0.1059	0.0894	0.1189
Coxibs	Female	70-79	JR≥5	0.0186	0.0425	0.0923	0.0776	0.1037
Coxibs	Female	80+	OA<5	0.0105	0.0242	0.0538	0.0450	0.0608
Coxibs	Female	80+	OA≥5	0.0086	0.0200	0.0447	0.0373	0.0506
Coxibs	Female	80+	JR<5	0.0125	0.0289	0.0638	0.0534	0.0720
Coxibs	Female	80+	JR≥5	0.0083	0.0192	0.0430	0.0359	0.0487
Opioids	Male	12-49	No OA	0.0043	0.0107	0.0276	0.0488	0.1150
Opioids	Male	50-59	No OA	0.0070	0.0176	0.0447	0.0781	0.1766
Opioids	Male	60-69	No OA	0.0058	0.0145	0.0371	0.0651	0.1499
Opioids	Male	70-79	No OA	0.0053	0.0131	0.0337	0.0594	0.1379
Opioids	Male	80+	No OA	0.0042	0.0105	0.0270	0.0478	0.1127
Opioids	Female	12-49	No OA	0.0046	0.0116	0.0300	0.0529	0.1239
Opioids	Female	50-59	No OA	0.0063	0.0158	0.0404	0.0708	0.1617
Opioids	Female	60-69	No OA	0.0056	0.0139	0.0356	0.0626	0.1447
Opioids	Female	70-79	No OA	0.0052	0.0130	0.0334	0.0589	0.1368
Opioids	Female	80+	No OA	0.0046	0.0114	0.0294	0.0519	0.1216
Opioids	Male	12-49	OA<5	0.0105	0.0260	0.0655	0.1125	0.2429
Opioids	Male	12-49	OA≥5	0.0073	0.0183	0.0466	0.0811	0.1827
Opioids	Male	12-49	JR<5	0.0180	0.0443	0.1083	0.1800	0.3573
Opioids	Male	12-49	JR≥5	0.0097	0.0240	0.0606	0.1045	0.2280
Opioids	Male	50-59	OA<5	0.0122	0.0302	0.0754	0.1285	0.2719
Opioids	Male	50-59	OA≥5	0.0131	0.0323	0.0805	0.1367	0.2862
Opioids	Male	50-59	JR<5	0.0117	0.0291	0.0729	0.1244	0.2646
Opioids	Male	50-59	JR≥5	0.0116	0.0288	0.0720	0.1231	0.2622
Opioids	Male	60-69	OA<5	0.0100	0.0249	0.0628	0.1081	0.2348
Opioids	Male	60-69	OA≥5	0.0122	0.0303	0.0758	0.1291	0.2730
Opioids	Male	60-69	JR<5	0.0138	0.0340	0.0845	0.1430	0.2971
Opioids	Male	60-69	JR≥5	0.0131	0.0325	0.0809	0.1373	0.2872
Opioids	Male	70-79	OA<5	0.0093	0.0232	0.0587	0.1013	0.2220
Opioids	Male	70-79	OA≥5	0.0086	0.0214	0.0543	0.0940	0.2080
Opioids	Male	70-79	JR<5	0.0109	0.0270	0.0678	0.1162	0.2498
Opioids	Male	70-79	JR≥5	0.0089	0.0221	0.0560	0.0969	0.2137
Opioids	Male	80+	OA<5	0.0075	0.0186	0.0473	0.0825	0.1854
Opioids	Male	80+	OA≥5	0.0068	0.0169	0.0432	0.0755	0.1714
Opioids	Male	80+	JR<5	0.0077	0.0193	0.0491	0.0854	0.1911

Opioids	Male	80+	JR≥5	0.0062	0.0156	0.0399	0.0698	0.1597
Opioids	Female	12-49	OA<5	0.0155	0.0382	0.0942	0.1583	0.3226
Opioids	Female	12-49	OA≥5	0.0121	0.0299	0.0746	0.1273	0.2697
Opioids	Female	12-49	JR<5	0.0178	0.0437	0.1069	0.1779	0.3540
Opioids	Female	12-49	JR≥5	0.0250	0.0607	0.1448	0.2345	0.4368
Opioids	Female	50-59	OA<5	0.0112	0.0277	0.0694	0.1189	0.2546
Opioids	Female	50-59	OA≥5	0.0130	0.0323	0.0804	0.1365	0.2858
Opioids	Female	50-59	JR<5	0.0198	0.0485	0.1179	0.1946	0.3796
Opioids	Female	50-59	JR≥5	0.0144	0.0355	0.0880	0.1486	0.3064
Opioids	Female	60-69	OA<5	0.0089	0.0222	0.0562	0.0971	0.2141
Opioids	Female	60-69	OA≥5	0.0114	0.0284	0.0711	0.1216	0.2596
Opioids	Female	60-69	JR<5	0.0186	0.0456	0.1113	0.1846	0.3644
Opioids	Female	60-69	JR≥5	0.0190	0.0466	0.1137	0.1882	0.3700
Opioids	Female	70-79	OA<5	0.0075	0.0187	0.0475	0.0827	0.1858
Opioids	Female	70-79	OA≥5	0.0096	0.0239	0.0603	0.1040	0.2272
Opioids	Female	70-79	JR<5	0.0103	0.0255	0.0641	0.1103	0.2389
Opioids	Female	70-79	JR≥5	0.0109	0.0270	0.0679	0.1164	0.2501
Opioids	Female	80+	OA<5	0.0061	0.0152	0.0390	0.0684	0.1567
Opioids	Female	80+	OA≥5	0.0066	0.0164	0.0418	0.0731	0.1665
Opioids	Female	80+	JR<5	0.0078	0.0195	0.0494	0.0860	0.1923
Opioids	Female	80+	JR≥5	0.0083	0.0206	0.0523	0.0907	0.2017

Table A1-6: Baseline incidence rates of cardiovascular disease (excluding stroke) per 1000 by sex, age and year.

Females	Age group								
Year	0-19	20-29	30-39	40-49	50-59	60-69	70-79	80-89	90+
2001	0.0915	0.8647	3.0007	9.1984	15.3848	33.4479	73.6154	119.8739	177.7839
2002	0.0912	0.8652	3.0049	9.1864	15.3857	33.4039	73.6683	120.0615	177.8628
2003	0.0909	0.8657	3.0089	9.1728	15.3860	33.3560	73.7055	120.2250	177.9110
2004	0.0907	0.8662	3.0127	9.1604	15.3871	33.3104	73.7077	120.3326	177.8615
2005	0.0908	0.8670	3.0164	9.1520	15.3904	33.2733	73.6553	120.3530	177.6478
2006	0.0911	0.8675	3.0180	9.1386	15.3730	33.1795	73.4637	120.2611	177.2855
2007	0.0913	0.8678	3.0168	9.1143	15.3248	33.0015	73.1287	120.0801	176.8437
2008	0.0914	0.8678	3.0145	9.0866	15.2664	32.8027	72.7520	119.8506	176.3362
2009	0.0914	0.8679	3.0127	9.0629	15.2184	32.6460	72.4350	119.6128	175.7765
2010	0.0912	0.8682	3.0131	9.0508	15.2014	32.5948	72.2794	119.4072	175.1782
2011	0.0910	0.8687	3.0153	9.0470	15.2061	32.6189	72.2470	119.2234	174.5371
2012	0.0906	0.8693	3.0176	9.0434	15.2098	32.6464	72.2359	119.0245	173.8408
2013	0.0902	0.8699	3.0200	9.0401	15.2128	32.6775	72.2516	118.8119	173.0902
2014	0.0896	0.8705	3.0226	9.0375	15.2153	32.7125	72.2997	118.5868	172.2864
2015	0.0891	0.8711	3.0255	9.0361	15.2179	32.7520	72.3859	118.3506	171.4303
2016	0.0886	0.8718	3.0287	9.0359	15.2208	32.7961	72.5157	118.1045	170.5231
2017	0.0882	0.8725	3.0322	9.0374	15.2243	32.8452	72.6947	117.8497	169.5658
Males	Age group								
Year	0-19	20-29	30-39	40-49	50-59	60-69	70-79	80-89	90+
2001	0.1255	0.8814	2.7990	8.5011	19.6218	46.0596	91.8752	144.5221	199.7099
2002	0.1254	0.8853	2.8178	8.5424	19.6283	45.8725	91.6025	144.3469	199.1996
2003	0.1250	0.8895	2.8386	8.5886	19.6363	45.6993	91.3303	144.1623	198.6747
2004	0.1249	0.8933	2.8571	8.6290	19.6432	45.5416	91.0642	143.9528	198.0213
2005	0.1254	0.8958	2.8688	8.6523	19.6463	45.4013	90.8102	143.7034	197.1254
2006	0.1261	0.8967	2.8694	8.6394	19.6096	45.2262	90.4753	143.3850	195.9847
2007	0.1267	0.8964	2.8613	8.5926	19.5201	44.9944	90.0273	143.0031	194.7060
2008	0.1271	0.8956	2.8495	8.5326	19.4106	44.7523	89.5636	142.5939	193.3388
2009	0.1271	0.8948	2.8393	8.4805	19.3137	44.5465	89.1815	142.1935	191.9327
2010	0.1269	0.8947	2.8360	8.4573	19.2620	44.4237	88.9785	141.8383	190.5374
2011	0.1264	0.8952	2.8378	8.4554	19.2440	44.3561	88.9040	141.5058	189.1530
2012	0.1257	0.8956	2.8396	8.4532	19.2271	44.2873	88.8469	141.1506	187.7467
2013	0.1249	0.8961	2.8413	8.4508	19.2119	44.2205	88.8134	140.7741	186.3184
2014	0.1239	0.8964	2.8431	8.4480	19.1991	44.1592	88.8095	140.3777	184.8678
2015	0.1228	0.8967	2.8449	8.4449	19.1893	44.1065	88.8416	139.9630	183.3951
2016	0.1218	0.8969	2.8469	8.4415	19.1831	44.0657	88.9157	139.5315	181.8999
2017	0.1211	0.8970	2.8491	8.4377	19.1814	44.0403	89.0380	139.0845	180.3822

Table A1-7: Baseline incidence rates of stroke per 1000 by sex, age and calendar year.

Females	Age group								
Year	0-19	20-29	30-39	40-49	50-59	60-69	70-79	80-89	90+
2001	0.1784	0.0917	0.3101	0.7940	1.4950	3.1774	5.7331	12.8362	25.3942
2002	0.1702	0.0908	0.3074	0.7873	1.4790	3.1529	5.6180	12.5919	25.0797
2003	0.1618	0.0899	0.3049	0.7809	1.4634	3.1278	5.5061	12.3446	24.7363
2004	0.1539	0.0892	0.3030	0.7762	1.4511	3.1060	5.4125	12.1192	24.3891
2005	0.1469	0.0888	0.3022	0.7743	1.4452	3.0916	5.3522	11.9409	24.0630
2006	0.1408	0.0890	0.3033	0.7763	1.4500	3.0875	5.3200	11.8045	23.7635
2007	0.1350	0.0894	0.3060	0.7811	1.4641	3.0914	5.2991	11.6865	23.4739
2008	0.1292	0.0900	0.3094	0.7874	1.4821	3.0991	5.2870	11.5853	23.1864
2009	0.1233	0.0905	0.3124	0.7934	1.4983	3.1065	5.2811	11.4990	22.8932
2010	0.1175	0.0907	0.3142	0.7977	1.5073	3.1098	5.2791	11.4257	22.5864
2011	0.1115	0.0907	0.3150	0.8006	1.5108	3.1095	5.2864	11.3595	22.2603
2012	0.1051	0.0907	0.3158	0.8035	1.5141	3.1092	5.3093	11.2969	21.9156
2013	0.0988	0.0908	0.3167	0.8063	1.5173	3.1089	5.3463	11.2394	21.5537
2014	0.0929	0.0907	0.3175	0.8090	1.5201	3.1087	5.3961	11.1884	21.1763
2015	0.0872	0.0907	0.3182	0.8115	1.5226	3.1086	5.4574	11.1456	20.7850
2016	0.0817	0.0906	0.3190	0.8139	1.5247	3.1086	5.5286	11.1124	20.3814
2017	0.0765	0.0905	0.3197	0.8161	1.5263	3.1087	5.6085	11.0903	19.9671
Males	Age group								
Year	0-19	20-29	30-39	40-49	50-59	60-69	70-79	80-89	90+
2001	0.1171	0.0769	0.2622	0.7656	1.7586	3.8010	6.8681	14.0301	25.6795
2002	0.1125	0.0766	0.2605	0.7614	1.7470	3.7510	6.7061	13.7776	25.4254
2003	0.1082	0.0763	0.2591	0.7577	1.7361	3.7022	6.5458	13.5186	25.1400
2004	0.1043	0.0761	0.2580	0.7550	1.7274	3.6593	6.4013	13.2732	24.8411
2005	0.1005	0.0760	0.2576	0.7540	1.7220	3.6268	6.2868	13.0614	24.5461
2006	0.0969	0.0762	0.2585	0.7556	1.7255	3.6028	6.1947	12.8801	24.2587
2007	0.0934	0.0766	0.2608	0.7595	1.7387	3.5825	6.1109	12.7131	23.9673
2008	0.0898	0.0771	0.2635	0.7643	1.7559	3.5665	6.0401	12.5609	23.6665
2009	0.0862	0.0776	0.2658	0.7685	1.7712	3.5553	5.9868	12.4242	23.3510
2010	0.0828	0.0777	0.2668	0.7707	1.7788	3.5496	5.9555	12.3035	23.0155
2011	0.0795	0.0776	0.2669	0.7714	1.7804	3.5480	5.9405	12.1893	22.6565
2012	0.0761	0.0775	0.2670	0.7720	1.7818	3.5489	5.9341	12.0720	22.2746
2013	0.0727	0.0772	0.2670	0.7726	1.7827	3.5529	5.9380	11.9526	21.8708
2014	0.0696	0.0769	0.2668	0.7731	1.7833	3.5604	5.9536	11.8320	21.4462
2015	0.0667	0.0765	0.2664	0.7735	1.7834	3.5719	5.9826	11.7109	21.0016
2016	0.0640	0.0760	0.2657	0.7736	1.7830	3.5877	6.0265	11.5905	20.5382
2017	0.0615	0.0754	0.2648	0.7734	1.7819	3.6083	6.0868	11.4715	20.0568

Table A1-8: Parameters determining the frequency of side effects of medication.

Condition	Parameter	Medication type			
		Acetaminophen	NSAIDS	Coxibs	Opioids
CVD	Relative risk vs. non-users	1.0	1.3	1.3	1.3
Stroke	Relative risk vs. non-users	1.0	1.1	1.1	1.1
Ulcer	Excess risk / 1000 users	0	7.4	1.1	7.4
Ulcer	Age multiplier for <70	NA	0.70	0.72	0.70
Ulcer	Age multiplier for 70+	NA	1.45	1.44	1.45
Dyspepsia	Excess risk / 1000 users	0	57.3	8.8	117.6
Overdose	Excess risk / 1000 users	0	0	0	1.26

Table A1-9: Probabilities of death from CVD (excluding stroke) by sex, age and year.

Females	Age group								
Year	0-19	20-29	30-39	40-49	50-59	60-69	70-79	80-89	90+
2001	0.0707	0.0176	0.0130	0.0131	0.0252	0.0447	0.0736	0.1697	0.3916
2002	0.0686	0.0172	0.0131	0.0132	0.0246	0.0430	0.0711	0.1659	0.3868
2003	0.0648	0.0174	0.0123	0.0128	0.0238	0.0406	0.0668	0.1593	0.3768
2004	0.0619	0.0173	0.0118	0.0123	0.0228	0.0378	0.0629	0.1527	0.3651
2005	0.0637	0.0172	0.0116	0.0125	0.0222	0.0361	0.0603	0.1466	0.3534
2006	0.0615	0.0165	0.0113	0.0121	0.0212	0.0337	0.0564	0.1388	0.3393
2007	0.0605	0.0163	0.0110	0.012 2	0.0211	0.0328	0.0540	0.1342	0.3311
2008	0.0581	0.0167	0.0114	0.0122	0.0211	0.0321	0.0525	0.1309	0.3247
2009	0.0553	0.0165	0.0114	0.0119	0.0204	0.0307	0.0496	0.1251	0.3137
2010	0.0542	0.0157	0.0106	0.0109	0.0194	0.0291	0.0464	0.1185	0.3035
2011	0.0524	0.0156	0.0106	0.0107	0.0193	0.0285	0.0450	0.1157	0.2973
2012	0.0528	0.0160	0.0106	0.0108	0.0192	0.0283	0.0441	0.1144	0.2938
2013	0.0551	0.0157	0.0103	0.0105	0.0186	0.0271	0.0428	0.1112	0.2854
2014	0.0562	0.0159	0.0106	0.0111	0.0191	0.0276	0.0446	0.1135	0.2843
2015	0.0574	0.0160	0.0108	0.0113	0.0194	0.0278	0.0452	0.1145	0.2868
2016	0.0570	0.0155	0.0105	0.0108	0.0189	0.0273	0.0442	0.1130	0.2876
2017	0.0560	0.0153	0.0103	0.0106	0.0187	0.0271	0.0439	0.1127	0.2896
Males	Age group								
Year	0-19	20-29	30-39	40-49	50-59	60-69	70-79	80-89	90+
2001	0.0616	0.0312	0.0345	0.0455	0.0634	0.0821	0.1163	0.2061	0.4023
2002	0.0614	0.0308	0.0343	0.0440	0.0606	0.0777	0.1097	0.1980	0.3957
2003	0.0617	0.0322	0.0348	0.0441	0.0594	0.0743	0.1058	0.1925	0.3885
2004	0.0606	0.0323	0.0343	0.0422	0.0561	0.0696	0.0982	0.1831	0.3747
2005	0.0615	0.0335	0.0338	0.0415	0.0544	0.0664	0.0926	0.1757	0.3652
2006	0.0587	0.0323	0.0325	0.0396	0.0518	0.0624	0.0862	0.1664	0.3516
2007	0.0577	0.0316	0.0324	0.0391	0.0524	0.0620	0.0839	0.1619	0.3439
2008	0.0556	0.0306	0.0311	0.0381	0.0519	0.0601	0.0799	0.1569	0.3375
2009	0.0517	0.0295	0.0301	0.0375	0.0500	0.0577	0.0754	0.1501	0.3277
2010	0.0486	0.0277	0.0293	0.0359	0.0480	0.0553	0.0713	0.1435	0.3197
2011	0.0446	0.0266	0.0272	0.0336	0.0452	0.0529	0.0676	0.1364	0.3088
2012	0.0438	0.0266	0.0274	0.0338	0.0459	0.0529	0.0671	0.1354	0.3080
2013	0.0457	0.0256	0.0276	0.0334	0.0452	0.0518	0.0652	0.1306	0.3002
2014	0.0484	0.0258	0.0274	0.0349	0.0470	0.0527	0.0669	0.1297	0.2960
2015	0.0503	0.0258	0.0271	0.0351	0.0477	0.0530	0.0677	0.1299	0.2985
2016	0.0498	0.0251	0.0261	0.0337	0.0466	0.0522	0.0666	0.1288	0.2999
2017	0.0490	0.0246	0.0256	0.0331	0.0460	0.0519	0.0663	0.1289	0.3020

Table A1-10: Probabilities of death from stroke by sex, age and year.

Females	Age group								
Year	0-19	20-29	30-39	40-49	50-59	60-69	70-79	80-89	90+
2001	0.0154	0.0777	0.0705	0.0711	0.0893	0.1288	0.3007	0.5838	0.9874
2002	0.0151	0.0725	0.0699	0.0703	0.0875	0.1274	0.3006	0.5843	0.9900
2003	0.0144	0.0722	0.0635	0.0673	0.0852	0.1227	0.2911	0.5743	0.9798
2004	0.0141	0.0697	0.0600	0.0638	0.0811	0.1142	0.2805	0.5613	0.9634
2005	0.0156	0.0675	0.0588	0.0650	0.0802	0.1099	0.2747	0.5482	0.9425
2006	0.0160	0.0655	0.0549	0.0634	0.0767	0.1038	0.2616	0.5282	0.9214
2007	0.0162	0.0623	0.0514	0.0631	0.0757	0.1018	0.2540	0.5221	0.9204
2008	0.0155	0.0639	0.0536	0.0622	0.0746	0.0988	0.2488	0.5182	0.9194
2009	0.0148	0.0601	0.0527	0.0581	0.0705	0.0937	0.2373	0.5026	0.8994
2010	0.0147	0.0563	0.0483	0.0523	0.0665	0.0870	0.2227	0.4792	0.8777
2011	0.0148	0.0566	0.0481	0.0511	0.0658	0.0843	0.2156	0.4700	0.8671
2012	0.0152	0.0559	0.0462	0.0496	0.0658	0.0836	0.2107	0.4657	0.8595
2013	0.0171	0.0577	0.0443	0.0481	0.0632	0.0802	0.2033	0.4513	0.8389
2014	0.0185	0.0586	0.0468	0.0519	0.0659	0.0830	0.2117	0.4650	0.8486
2015	0.0202	0.0591	0.0478	0.0531	0.0673	0.0845	0.2130	0.4714	0.8676
2016	0.0212	0.0572	0.0465	0.0512	0.0659	0.0839	0.2068	0.4674	0.8836
2017	0.0221	0.0560	0.0457	0.0504	0.0653	0.0841	0.2034	0.4669	0.9028
Males	Age group								
Year	0-19	20-29	30-39	40-49	50-59	60-69	70-79	80-89	90+
2001	0.0231	0.1052	0.0722	0.0809	0.1006	0.1662	0.3669	0.6146	0.9599
2002	0.0239	0.1013	0.0731	0.0794	0.0979	0.1588	0.3563	0.6028	0.9568
2003	0.0241	0.1029	0.0736	0.0792	0.0966	0.1523	0.3517	0.5966	0.9503
2004	0.0244	0.1068	0.0738	0.0767	0.0926	0.1440	0.3338	0.5727	0.9160
2005	0.0261	0.1096	0.0738	0.0764	0.0915	0.1397	0.3207	0.5570	0.9020
2006	0.0255	0.1056	0.0693	0.0724	0.0869	0.1315	0.3049	0.5389	0.8838
2007	0.0260	0.1029	0.0693	0.0721	0.0888	0.1315	0.3021	0.5365	0.8814
2008	0.0256	0.0996	0.0662	0.0700	0.0864	0.1272	0.2917	0.5293	0.8758
2009	0.0244	0.0970	0.0633	0.0683	0.0829	0.1229	0.2791	0.5164	0.8616
2010	0.0235	0.0916	0.0595	0.0662	0.0803	0.1186	0.2652	0.4994	0.8455
2011	0.0221	0.0843	0.0530	0.0627	0.0754	0.1131	0.2499	0.4771	0.8223
2012	0.0220	0.0850	0.0528	0.0636	0.0775	0.1135	0.2475	0.4777	0.8240
2013	0.0236	0.0814	0.0524	0.0623	0.0758	0.1100	0.2389	0.4610	0.8080
2014	0.0262	0.0830	0.0533	0.0660	0.0799	0.1132	0.2472	0.4644	0.8108
2015	0.0285	0.0838	0.0531	0.0667	0.0818	0.1145	0.2502	0.4706	0.8303
2016	0.0291	0.0815	0.0516	0.0641	0.0804	0.1130	0.2458	0.4736	0.8486
2017	0.0295	0.0803	0.0511	0.0631	0.0799	0.1124	0.2435	0.4795	0.8691

Table A1-11: Probability of death due to side effects of treatment other than CVD or stroke.

Side effect	Probability
Ulcer (bleeding or perforation)	4.0%
Dyspepsia	0.0%
Overdose (lethal)	100%

Table A1-12: Relative (%) reduction in pain (measured on a numerical scale) resulting from the use of pain medication.

Drug class	Pain reduction (%)
NSAIDS	38.2
Coxibs	38.2
Acetaminophen	28.2
Opioids	40.2

Table A1-13: Effect of age, sex and time on the risk of primary and revision JRS.

Variable	Primary JRS		Revision JRS	
	Coefficient	Hazard ratio	Coefficient	Hazard ratio
Intercept	-5.4861	0.0041	-7.1397	0.0008
Log_{10}(year-2000)	0.3834	1.4673	0.0169	1.0170
Age 40-49	0.7394	2.0947	0.5365	1.7100
Age 50-59	1.4081	4.0882	0.9212	2.5123
Age 60-69	1.8060	6.0861	1.1739	3.2346
Age 70-79	1.8991	6.6799	1.4652	4.3284
Age 80-89	1.2318	3.4274	1.1756	3.2401
Age <40 (ref)	0.0000	1.0000	0.0000	1.0000
Female	-0.0795	0.9236	-0.2113	0.8095

The coefficients were estimated from a Poisson regression model.

Table A1-14: Effect of HUI3 domains on the risk of primary and revision JRS.

Variable	Primary JRS		Revision JRS	
	Coefficient	Odds ratio	Coefficient	Odds ratio
Vision	-0.6162	0.542	-0.6162	0.542
Hearing	0.0329	1.033	0.0329	1.033
Speech	2.9272	18.676	2.9272	18.676
Mobility	2.9475	19.059	2.9475	19.059
Dexterity	0.6779	1.970	0.6779	1.970
Emotion	0.6361	1.889	0.6361	1.889
Cognition	-0.9075	0.404	-0.9075	0.404
Pain level 2	3.7812	43.868	3.7812	43.868
Pain levels 3-5	4.3370	76.480	4.3370	76.480

The coefficients were obtained from a conditional logistic regression model.

Table A-15: Rates of primary JRS per 1000 person-years by age, sex and year.

Males						
Year \ Age	<40	40-49	50-59	60-69	70-79	80+
2001	4.14	8.68	16.94	25.22	27.68	14.20
2002	4.65	9.74	19.01	28.31	31.07	15.94
2003	4.98	10.42	20.34	30.28	33.24	17.06
2004	5.22	10.93	21.34	31.77	34.87	17.89
2005	5.42	11.35	22.15	32.97	36.19	18.57
2006	5.58	11.70	22.83	33.99	37.30	19.14
2007	5.73	12.00	23.43	34.87	38.27	19.64
2008	5.86	12.27	23.95	35.66	39.14	20.08
2009	5.97	12.52	24.43	36.36	39.91	20.48
2010	6.08	12.74	24.86	37.01	40.62	20.84
2011	6.18	12.94	25.26	37.60	41.27	21.17
2012	6.27	13.13	25.63	38.15	41.87	21.48
2013	6.35	13.31	25.97	38.66	42.43	21.77
2014	6.43	13.47	26.29	39.14	42.96	22.04
2015	6.51	13.63	26.60	39.59	43.45	22.30
2016	6.58	13.77	26.88	40.02	43.92	22.54
2017	6.64	13.91	27.16	40.43	44.37	22.77
2018	6.71	14.05	27.42	40.81	44.79	22.98
2019	6.77	14.17	27.66	41.18	45.20	23.19
2020	6.82	14.30	27.90	41.54	45.59	23.39
2021	6.88	14.41	28.13	41.87	45.96	23.58
2022	6.93	14.52	28.35	42.20	46.32	23.77
2023	6.99	14.63	28.56	42.51	46.66	23.94
2024	7.04	14.74	28.76	42.82	46.99	24.11
2025	7.08	14.84	28.96	43.11	47.31	24.28
2026	7.13	14.93	29.15	43.39	47.62	24.44
2027	7.17	15.03	29.33	43.66	47.92	24.59
2028	7.22	15.12	29.51	43.93	48.21	24.74
2029	7.26	15.21	29.68	44.19	48.50	24.88
2030	7.30	15.29	29.85	44.44	48.77	25.03
2031	7.34	15.38	30.01	44.68	49.04	25.16
2032	7.38	15.46	30.17	44.92	49.30	25.30
2033	7.42	15.54	30.33	45.15	49.55	25.43
2034	7.46	15.62	30.48	45.37	49.80	25.55
2035	7.49	15.69	30.63	45.59	50.04	25.68
2036	7.53	15.77	30.77	45.81	50.27	25.80
2037	7.56	15.84	30.91	46.02	50.50	25.91
2038	7.59	15.91	31.05	46.22	50.73	26.03
2039	7.63	15.98	31.18	46.42	50.95	26.14
2040	7.66	16.05	31.31	46.62	51.16	26.25
2041	7.69	16.11	31.44	46.81	51.38	26.36
2042	7.72	16.18	31.57	47.00	51.58	26.47

2043	7.75	16.24	31.69	47.18	51.78	26.57
2044	7.78	16.30	31.82	47.36	51.98	26.67
2045	7.81	16.36	31.93	47.54	52.18	26.77
2046	7.84	16.42	32.05	47.72	52.37	26.87
2047	7.87	16.48	32.17	47.89	52.56	26.97
2048	7.90	16.54	32.28	48.05	52.74	27.06
2049	7.92	16.60	32.39	48.22	52.92	27.16
2050	7.95	16.65	32.50	48.38	53.10	27.25
2051	7.98	16.71	32.61	48.54	53.28	27.34
2052	8.00	16.76	32.71	48.70	53.45	27.43
2053	8.03	16.81	32.82	48.85	53.62	27.51
2054	8.05	16.87	32.92	49.01	53.79	27.60
2055	8.08	16.92	33.02	49.16	53.95	27.68
2056	8.10	16.97	33.12	49.30	54.11	27.77
2057	8.13	17.02	33.22	49.45	54.27	27.85
2058	8.15	17.07	33.31	49.59	54.43	27.93
2059	8.17	17.12	33.41	49.73	54.59	28.01
2060	8.19	17.17	33.50	49.87	54.74	28.09
2061	8.22	17.21	33.59	50.01	54.89	28.16
2062	8.24	17.26	33.69	50.15	55.04	28.24
2063	8.26	17.31	33.78	50.28	55.19	28.32
2064	8.28	17.35	33.86	50.41	55.33	28.39
2065	8.30	17.40	33.95	50.54	55.47	28.46
2066	8.33	17.44	34.04	50.67	55.61	28.54
2067	8.35	17.48	34.12	50.80	55.75	28.61
2068	8.37	17.53	34.21	50.92	55.89	28.68
2069	8.39	17.57	34.29	51.05	56.03	28.75
2070	8.41	17.61	34.37	51.17	56.16	28.82
2071	8.43	17.65	34.45	51.29	56.29	28.89
2072	8.45	17.69	34.53	51.41	56.43	28.95
2073	8.47	17.74	34.61	51.53	56.56	29.02
2074	8.49	17.78	34.69	51.65	56.68	29.09
2075	8.50	17.82	34.77	51.76	56.81	29.15
2076	8.52	17.86	34.85	51.88	56.94	29.22
2077	8.54	17.89	34.92	51.99	57.06	29.28
2078	8.56	17.93	35.00	52.10	57.18	29.34
2079	8.58	17.97	35.07	52.21	57.30	29.40
2080	8.60	18.01	35.15	52.32	57.42	29.47
2081	8.61	18.05	35.22	52.43	57.54	29.53
2082	8.63	18.08	35.29	52.54	57.66	29.59
2083	8.65	18.12	35.36	52.64	57.78	29.65
2084	8.67	18.16	35.43	52.75	57.89	29.71
2085	8.68	18.19	35.50	52.85	58.01	29.76
2086	8.70	18.23	35.57	52.96	58.12	29.82
2087	8.72	18.26	35.64	53.06	58.23	29.88
2088	8.73	18.30	35.71	53.16	58.34	29.94

2089	8.75	18.33	35.78	53.26	58.45	29.99
2090	8.77	18.36	35.84	53.36	58.56	30.05
2091	8.78	18.40	35.91	53.46	58.67	30.10
2092	8.80	18.43	35.97	53.55	58.78	30.16
2093	8.82	18.47	36.04	53.65	58.88	30.21
2094	8.83	18.50	36.10	53.75	58.99	30.27
2095	8.85	18.53	36.17	53.84	59.09	30.32
2096	8.86	18.56	36.23	53.93	59.19	30.37
2097	8.88	18.60	36.29	54.03	59.30	30.43
2098	8.89	18.63	36.35	54.12	59.40	30.48
2099	8.91	18.66	36.42	54.21	59.50	30.53
2100	8.92	18.69	36.48	54.30	59.60	30.58
Females						
Year \ Age	<40	40-49	50-59	60-69	70-79	80+
2001	3.83	8.02	15.65	23.29	25.56	13.12
2002	4.30	9.00	17.56	26.14	28.69	14.72
2003	4.60	9.63	18.79	27.97	30.70	15.75
2004	4.82	10.10	19.71	29.34	32.20	16.52
2005	5.00	10.48	20.46	30.45	33.42	17.15
2006	5.16	10.80	21.09	31.39	34.45	17.68
2007	5.29	11.09	21.63	32.21	35.35	18.14
2008	5.41	11.33	22.12	32.93	36.14	18.55
2009	5.52	11.56	22.56	33.58	36.86	18.91
2010	5.62	11.76	22.96	34.18	37.51	19.25
2011	5.71	11.95	23.33	34.72	38.11	19.56
2012	5.79	12.13	23.67	35.23	38.67	19.84
2013	5.87	12.29	23.98	35.70	39.19	20.11
2014	5.94	12.44	24.28	36.15	39.67	20.36
2015	6.01	12.59	24.56	36.57	40.13	20.59
2016	6.07	12.72	24.83	36.96	40.57	20.81
2017	6.13	12.85	25.08	37.34	40.98	21.03
2018	6.19	12.97	25.32	37.69	41.37	21.23
2019	6.25	13.09	25.55	38.03	41.74	21.42
2020	6.30	13.20	25.77	38.36	42.10	21.60
2021	6.35	13.31	25.98	38.67	42.44	21.78
2022	6.40	13.41	26.18	38.97	42.77	21.95
2023	6.45	13.51	26.37	39.26	43.09	22.11
2024	6.50	13.61	26.56	39.54	43.40	22.27
2025	6.54	13.70	26.74	39.81	43.69	22.42
2026	6.58	13.79	26.92	40.07	43.98	22.57
2027	6.63	13.88	27.09	40.33	44.26	22.71
2028	6.67	13.96	27.25	40.57	44.53	22.85
2029	6.71	14.05	27.41	40.81	44.79	22.98
2030	6.74	14.13	27.57	41.04	45.04	23.11
2031	6.78	14.20	27.72	41.26	45.29	23.24
2032	6.82	14.28	27.87	41.48	45.53	23.36

2033	6.85	14.35	28.01	41.70	45.76	23.48
2034	6.89	14.42	28.15	41.90	45.99	23.60
2035	6.92	14.49	28.28	42.11	46.21	23.71
2036	6.95	14.56	28.42	42.30	46.43	23.82
2037	6.98	14.63	28.55	42.50	46.64	23.93
2038	7.01	14.69	28.67	42.69	46.85	24.04
2039	7.04	14.76	28.80	42.87	47.05	24.14
2040	7.07	14.82	28.92	43.05	47.25	24.25
2041	7.10	14.88	29.04	43.23	47.45	24.35
2042	7.13	14.94	29.16	43.40	47.64	24.44
2043	7.16	15.00	29.27	43.57	47.82	24.54
2044	7.19	15.06	29.38	43.74	48.01	24.63
2045	7.21	15.11	29.49	43.91	48.19	24.73
2046	7.24	15.17	29.60	44.07	48.36	24.82
2047	7.27	15.22	29.71	44.23	48.54	24.91
2048	7.29	15.27	29.81	44.38	48.71	24.99
2049	7.32	15.33	29.91	44.53	48.88	25.08
2050	7.34	15.38	30.01	44.68	49.04	25.16
2051	7.37	15.43	30.11	44.83	49.20	25.25
2052	7.39	15.48	30.21	44.98	49.36	25.33
2053	7.41	15.53	30.31	45.12	49.52	25.41
2054	7.44	15.58	30.40	45.26	49.67	25.49
2055	7.46	15.63	30.50	45.40	49.83	25.57
2056	7.48	15.67	30.59	45.53	49.98	25.64
2057	7.50	15.72	30.68	45.67	50.12	25.72
2058	7.53	15.76	30.77	45.80	50.27	25.79
2059	7.55	15.81	30.85	45.93	50.41	25.87
2060	7.57	15.85	30.94	46.06	50.55	25.94
2061	7.59	15.90	31.03	46.19	50.69	26.01
2062	7.61	15.94	31.11	46.31	50.83	26.08
2063	7.63	15.98	31.19	46.44	50.97	26.15
2064	7.65	16.02	31.27	46.56	51.10	26.22
2065	7.67	16.07	31.36	46.68	51.23	26.29
2066	7.69	16.11	31.44	46.80	51.36	26.35
2067	7.71	16.15	31.51	46.91	51.49	26.42
2068	7.73	16.19	31.59	47.03	51.62	26.49
2069	7.75	16.23	31.67	47.15	51.74	26.55
2070	7.76	16.27	31.74	47.26	51.87	26.61
2071	7.78	16.30	31.82	47.37	51.99	26.68
2072	7.80	16.34	31.89	47.48	52.11	26.74
2073	7.82	16.38	31.97	47.59	52.23	26.80
2074	7.84	16.42	32.04	47.70	52.35	26.86
2075	7.85	16.45	32.11	47.80	52.47	26.92
2076	7.87	16.49	32.18	47.91	52.58	26.98
2077	7.89	16.53	32.25	48.01	52.70	27.04
2078	7.91	16.56	32.32	48.12	52.81	27.10

2079	7.92	16.60	32.39	48.22	52.92	27.16
2080	7.94	16.63	32.46	48.32	53.03	27.21
2081	7.96	16.67	32.53	48.42	53.14	27.27
2082	7.97	16.70	32.59	48.52	53.25	27.32
2083	7.99	16.73	32.66	48.62	53.36	27.38
2084	8.00	16.77	32.72	48.72	53.47	27.43
2085	8.02	16.80	32.79	48.81	53.57	27.49
2086	8.04	16.83	32.85	48.91	53.68	27.54
2087	8.05	16.87	32.91	49.00	53.78	27.60
2088	8.07	16.90	32.98	49.09	53.88	27.65
2089	8.08	16.93	33.04	49.19	53.98	27.70
2090	8.10	16.96	33.10	49.28	54.08	27.75
2091	8.11	16.99	33.16	49.37	54.18	27.80
2092	8.13	17.02	33.22	49.46	54.28	27.85
2093	8.14	17.05	33.28	49.55	54.38	27.90
2094	8.16	17.08	33.34	49.64	54.48	27.95
2095	8.17	17.11	33.40	49.72	54.57	28.00
2096	8.18	17.14	33.46	49.81	54.67	28.05
2097	8.20	17.17	33.52	49.90	54.76	28.10
2098	8.21	17.20	33.57	49.98	54.86	28.15
2099	8.23	17.23	33.63	50.07	54.95	28.20
2100	8.24	17.26	33.69	50.15	55.04	28.24

Table A1-16: Rates of revision JRS per 1000 person-years by age, sex and year.

Males						
Year \ Age	<40	40-49	50-59	60-69	70-79	80+
2001	0.79	1.36	1.99	2.56	3.43	2.57
2002	0.80	1.36	2.00	2.58	3.45	2.58
2003	0.80	1.37	2.01	2.59	3.46	2.59
2004	0.80	1.37	2.01	2.59	3.47	2.60
2005	0.80	1.37	2.02	2.60	3.47	2.60
2006	0.80	1.37	2.02	2.60	3.48	2.60
2007	0.80	1.38	2.02	2.60	3.48	2.61
2008	0.81	1.38	2.02	2.60	3.49	2.61
2009	0.81	1.38	2.02	2.61	3.49	2.61
2010	0.81	1.38	2.03	2.61	3.49	2.61
2011	0.81	1.38	2.03	2.61	3.49	2.61
2012	0.81	1.38	2.03	2.61	3.50	2.62
2013	0.81	1.38	2.03	2.61	3.50	2.62
2014	0.81	1.38	2.03	2.62	3.50	2.62
2015	0.81	1.38	2.03	2.62	3.50	2.62
2016	0.81	1.38	2.03	2.62	3.50	2.62
2017	0.81	1.38	2.03	2.62	3.50	2.62
2018	0.81	1.39	2.04	2.62	3.51	2.62
2019	0.81	1.39	2.04	2.62	3.51	2.63
2020	0.81	1.39	2.04	2.62	3.51	2.63
2021	0.81	1.39	2.04	2.62	3.51	2.63
2022	0.81	1.39	2.04	2.62	3.51	2.63
2023	0.81	1.39	2.04	2.62	3.51	2.63
2024	0.81	1.39	2.04	2.63	3.51	2.63
2025	0.81	1.39	2.04	2.63	3.51	2.63
2026	0.81	1.39	2.04	2.63	3.52	2.63
2027	0.81	1.39	2.04	2.63	3.52	2.63
2028	0.81	1.39	2.04	2.63	3.52	2.63
2029	0.81	1.39	2.04	2.63	3.52	2.63
2030	0.81	1.39	2.04	2.63	3.52	2.63
2031	0.81	1.39	2.04	2.63	3.52	2.63
2032	0.81	1.39	2.04	2.63	3.52	2.64
2033	0.81	1.39	2.04	2.63	3.52	2.64
2034	0.81	1.39	2.04	2.63	3.52	2.64
2035	0.81	1.39	2.04	2.63	3.52	2.64
2036	0.81	1.39	2.05	2.63	3.52	2.64
2037	0.81	1.39	2.05	2.63	3.52	2.64
2038	0.81	1.39	2.05	2.63	3.53	2.64
2039	0.81	1.39	2.05	2.63	3.53	2.64
2040	0.81	1.39	2.05	2.64	3.53	2.64
2041	0.81	1.39	2.05	2.64	3.53	2.64
2042	0.82	1.39	2.05	2.64	3.53	2.64

2043	0.82	1.39	2.05	2.64	3.53	2.64
2044	0.82	1.39	2.05	2.64	3.53	2.64
2045	0.82	1.39	2.05	2.64	3.53	2.64
2046	0.82	1.39	2.05	2.64	3.53	2.64
2047	0.82	1.39	2.05	2.64	3.53	2.64
2048	0.82	1.40	2.05	2.64	3.53	2.64
2049	0.82	1.40	2.05	2.64	3.53	2.64
2050	0.82	1.40	2.05	2.64	3.53	2.64
2051	0.82	1.40	2.05	2.64	3.53	2.64
2052	0.82	1.40	2.05	2.64	3.53	2.64
2053	0.82	1.40	2.05	2.64	3.53	2.65
2054	0.82	1.40	2.05	2.64	3.53	2.65
2055	0.82	1.40	2.05	2.64	3.53	2.65
2056	0.82	1.40	2.05	2.64	3.54	2.65
2057	0.82	1.40	2.05	2.64	3.54	2.65
2058	0.82	1.40	2.05	2.64	3.54	2.65
2059	0.82	1.40	2.05	2.64	3.54	2.65
2060	0.82	1.40	2.05	2.64	3.54	2.65
2061	0.82	1.40	2.05	2.64	3.54	2.65
2062	0.82	1.40	2.05	2.64	3.54	2.65
2063	0.82	1.40	2.05	2.64	3.54	2.65
2064	0.82	1.40	2.05	2.64	3.54	2.65
2065	0.82	1.40	2.05	2.64	3.54	2.65
2066	0.82	1.40	2.05	2.64	3.54	2.65
2067	0.82	1.40	2.05	2.65	3.54	2.65
2068	0.82	1.40	2.05	2.65	3.54	2.65
2069	0.82	1.40	2.06	2.65	3.54	2.65
2070	0.82	1.40	2.06	2.65	3.54	2.65
2071	0.82	1.40	2.06	2.65	3.54	2.65
2072	0.82	1.40	2.06	2.65	3.54	2.65
2073	0.82	1.40	2.06	2.65	3.54	2.65
2074	0.82	1.40	2.06	2.65	3.54	2.65
2075	0.82	1.40	2.06	2.65	3.54	2.65
2076	0.82	1.40	2.06	2.65	3.54	2.65
2077	0.82	1.40	2.06	2.65	3.54	2.65
2078	0.82	1.40	2.06	2.65	3.54	2.65
2079	0.82	1.40	2.06	2.65	3.54	2.65
2080	0.82	1.40	2.06	2.65	3.54	2.65
2081	0.82	1.40	2.06	2.65	3.54	2.65
2082	0.82	1.40	2.06	2.65	3.55	2.65
2083	0.82	1.40	2.06	2.65	3.55	2.65
2084	0.82	1.40	2.06	2.65	3.55	2.65
2085	0.82	1.40	2.06	2.65	3.55	2.65
2086	0.82	1.40	2.06	2.65	3.55	2.65
2087	0.82	1.40	2.06	2.65	3.55	2.65
2088	0.82	1.40	2.06	2.65	3.55	2.66

2089	0.82	1.40	2.06	2.65	3.55	2.66
2090	0.82	1.40	2.06	2.65	3.55	2.66
2091	0.82	1.40	2.06	2.65	3.55	2.66
2092	0.82	1.40	2.06	2.65	3.55	2.66
2093	0.82	1.40	2.06	2.65	3.55	2.66
2094	0.82	1.40	2.06	2.65	3.55	2.66
2095	0.82	1.40	2.06	2.65	3.55	2.66
2096	0.82	1.40	2.06	2.65	3.55	2.66
2097	0.82	1.40	2.06	2.65	3.55	2.66
2098	0.82	1.40	2.06	2.65	3.55	2.66
2099	0.82	1.40	2.06	2.65	3.55	2.66
2100	0.82	1.40	2.06	2.65	3.55	2.66
Females						
Year \ Age	<40	40-49	50-59	60-69	70-79	80+
2001	0.64	1.10	1.61	2.08	2.78	2.08
2002	0.65	1.10	1.62	2.09	2.79	2.09
2003	0.65	1.11	1.63	2.09	2.80	2.10
2004	0.65	1.11	1.63	2.10	2.81	2.10
2005	0.65	1.11	1.63	2.10	2.81	2.10
2006	0.65	1.11	1.63	2.10	2.82	2.11
2007	0.65	1.11	1.64	2.11	2.82	2.11
2008	0.65	1.11	1.64	2.11	2.82	2.11
2009	0.65	1.12	1.64	2.11	2.82	2.11
2010	0.65	1.12	1.64	2.11	2.83	2.12
2011	0.65	1.12	1.64	2.11	2.83	2.12
2012	0.65	1.12	1.64	2.11	2.83	2.12
2013	0.65	1.12	1.64	2.12	2.83	2.12
2014	0.65	1.12	1.64	2.12	2.83	2.12
2015	0.65	1.12	1.65	2.12	2.83	2.12
2016	0.66	1.12	1.65	2.12	2.84	2.12
2017	0.66	1.12	1.65	2.12	2.84	2.12
2018	0.66	1.12	1.65	2.12	2.84	2.12
2019	0.66	1.12	1.65	2.12	2.84	2.13
2020	0.66	1.12	1.65	2.12	2.84	2.13
2021	0.66	1.12	1.65	2.12	2.84	2.13
2022	0.66	1.12	1.65	2.12	2.84	2.13
2023	0.66	1.12	1.65	2.12	2.84	2.13
2024	0.66	1.12	1.65	2.13	2.84	2.13
2025	0.66	1.12	1.65	2.13	2.84	2.13
2026	0.66	1.12	1.65	2.13	2.85	2.13
2027	0.66	1.12	1.65	2.13	2.85	2.13
2028	0.66	1.12	1.65	2.13	2.85	2.13
2029	0.66	1.13	1.65	2.13	2.85	2.13
2030	0.66	1.13	1.65	2.13	2.85	2.13
2031	0.66	1.13	1.65	2.13	2.85	2.13
2032	0.66	1.13	1.65	2.13	2.85	2.13

2033	0.66	1.13	1.65	2.13	2.85	2.13
2034	0.66	1.13	1.66	2.13	2.85	2.13
2035	0.66	1.13	1.66	2.13	2.85	2.13
2036	0.66	1.13	1.66	2.13	2.85	2.14
2037	0.66	1.13	1.66	2.13	2.85	2.14
2038	0.66	1.13	1.66	2.13	2.85	2.14
2039	0.66	1.13	1.66	2.13	2.85	2.14
2040	0.66	1.13	1.66	2.13	2.85	2.14
2041	0.66	1.13	1.66	2.13	2.86	2.14
2042	0.66	1.13	1.66	2.13	2.86	2.14
2043	0.66	1.13	1.66	2.13	2.86	2.14
2044	0.66	1.13	1.66	2.13	2.86	2.14
2045	0.66	1.13	1.66	2.14	2.86	2.14
2046	0.66	1.13	1.66	2.14	2.86	2.14
2047	0.66	1.13	1.66	2.14	2.86	2.14
2048	0.66	1.13	1.66	2.14	2.86	2.14
2049	0.66	1.13	1.66	2.14	2.86	2.14
2050	0.66	1.13	1.66	2.14	2.86	2.14
2051	0.66	1.13	1.66	2.14	2.86	2.14
2052	0.66	1.13	1.66	2.14	2.86	2.14
2053	0.66	1.13	1.66	2.14	2.86	2.14
2054	0.66	1.13	1.66	2.14	2.86	2.14
2055	0.66	1.13	1.66	2.14	2.86	2.14
2056	0.66	1.13	1.66	2.14	2.86	2.14
2057	0.66	1.13	1.66	2.14	2.86	2.14
2058	0.66	1.13	1.66	2.14	2.86	2.14
2059	0.66	1.13	1.66	2.14	2.86	2.14
2060	0.66	1.13	1.66	2.14	2.86	2.14
2061	0.66	1.13	1.66	2.14	2.86	2.14
2062	0.66	1.13	1.66	2.14	2.86	2.14
2063	0.66	1.13	1.66	2.14	2.86	2.14
2064	0.66	1.13	1.66	2.14	2.86	2.14
2065	0.66	1.13	1.66	2.14	2.86	2.14
2066	0.66	1.13	1.66	2.14	2.87	2.14
2067	0.66	1.13	1.66	2.14	2.87	2.15
2068	0.66	1.13	1.66	2.14	2.87	2.15
2069	0.66	1.13	1.66	2.14	2.87	2.15
2070	0.66	1.13	1.66	2.14	2.87	2.15
2071	0.66	1.13	1.66	2.14	2.87	2.15
2072	0.66	1.13	1.66	2.14	2.87	2.15
2073	0.66	1.13	1.66	2.14	2.87	2.15
2074	0.66	1.13	1.66	2.14	2.87	2.15
2075	0.66	1.13	1.66	2.14	2.87	2.15
2076	0.66	1.13	1.66	2.14	2.87	2.15
2077	0.66	1.13	1.67	2.14	2.87	2.15
2078	0.66	1.13	1.67	2.14	2.87	2.15

2079	0.66	1.13	1.67	2.14	2.87	2.15
2080	0.66	1.13	1.67	2.14	2.87	2.15
2081	0.66	1.13	1.67	2.14	2.87	2.15
2082	0.66	1.13	1.67	2.14	2.87	2.15
2083	0.66	1.13	1.67	2.14	2.87	2.15
2084	0.66	1.13	1.67	2.14	2.87	2.15
2085	0.66	1.13	1.67	2.15	2.87	2.15
2086	0.66	1.13	1.67	2.15	2.87	2.15
2087	0.66	1.13	1.67	2.15	2.87	2.15
2088	0.66	1.13	1.67	2.15	2.87	2.15
2089	0.66	1.13	1.67	2.15	2.87	2.15
2090	0.66	1.13	1.67	2.15	2.87	2.15
2091	0.66	1.13	1.67	2.15	2.87	2.15
2092	0.66	1.13	1.67	2.15	2.87	2.15
2093	0.66	1.13	1.67	2.15	2.87	2.15
2094	0.66	1.13	1.67	2.15	2.87	2.15
2095	0.66	1.14	1.67	2.15	2.87	2.15
2096	0.66	1.14	1.67	2.15	2.87	2.15
2097	0.66	1.14	1.67	2.15	2.87	2.15
2098	0.66	1.14	1.67	2.15	2.87	2.15
2099	0.66	1.14	1.67	2.15	2.87	2.15
2100	0.66	1.14	1.67	2.15	2.87	2.15

Table A1-17: Estimated coefficients in models for each HUI3 domain.

Parameter	Emotion	Mobility	Dexterity	Cognition	Pain	Speech	Hearing	Vision
Intercept 2	-2.3940	-6.7368	-6.6789	-2.2430	-3.6410	-6.6061	-5.5793	-2.1406
Intercept 3	-5.0244	-7.0893		-2.4303	-4.1129		-7.2977	-8.4216
Intercept 4	-6.8009	-9.0861		-4.2130	-5.0737			
Intercept 5				-5.6484	-6.3890			
Female	-0.0994	0.0903	0.2594	-0.0360	0.1363	-0.4899	-0.3976	0.3063
Age 30-39	0.0754	0.5653	-0.6252	-0.0515	0.3179	-0.3488	0.2902	-0.0294
Age 40-49	0.0822	0.8646	-0.0841	-0.2743	0.5789	0.1996	0.8617	0.8131
Age 50-59	0.0440	1.1842	-0.2801	-0.2887	0.5528	-0.2309	1.1592	1.2820
Age 60-69	-0.1501	1.3928	0.1096	-0.3082	0.3028	0.0909	1.6567	1.2769
Age 70-79	-0.1433	2.1129	0.4175	-0.0366	0.2894	-0.0354	2.3701	1.2679
Age 80+	-0.0843	2.9860	0.2798	0.1157	0.4408	0.3823	2.9878	1.5193
Educ. grp 1	-0.0497	0.1819	0.5013	0.2064	0.1184	0.8678	0.0002	-0.0159
Educ. grp 2	-0.0634	-0.0072	-0.1191	0.1154	0.1768	0.9022	-0.0182	-0.1441
Educ. grp 3	-0.0366	0.2200	0.1993	0.1428	0.1580	0.0545	0.1177	-0.0907
Income quart 1	0.5081	0.7242	0.3092	0.3663	0.5985	1.3944	0.0623	0.0650
Income quart 2	0.3451	0.5288	0.2609	0.2339	0.3760	1.1431	0.2073	0.0631
Income quart 3	0.2362	0.3567	0.2267	0.1301	0.2014	0.3322	0.2774	0.0259
Smoking	0.2240	0.0082	0.1095	0.0284	0.2131	0.0611	0.1824	0.0520
BMI <18.5	0.2268	0.7516	0.2456	-0.0802	0.1747	-0.1351	0.3366	0.2930
BMI 25.0-29.9	-0.0783	-0.1601	-0.2696	-0.0717	0.0085	-0.5682	-0.0268	0.0343
BMI 30+	-0.0519	0.1963	-0.0285	0.0295	0.2798	-0.7315	-0.0102	-0.0058
HUI3 Emotion 2YR 2	1.6027							
HUI3 Emotion 2YR 3	2.5555							
HUI3 Emotion 2YR 4	3.2375							
HUI3 Mobility 2YR 2		2.0441						
HUI3 Mobility 2YR 3		3.1042						
HUI3 Mobility 2YR 4		4.4582						
HUI3 Dexterity 2YR 2			3.2772					
HUI3 Cognition 2YR 2				1.1752				
HUI3 Cognition 2YR 3				1.7614				
HUI3 Cognition 2YR 4				2.5809				
HUI3 Cognition 2YR 5				3.8584				
HUI3 Pain 2YR 2					1.6031			
HUI3 Pain 2YR 3					2.1149			
HUI3 Pain 2YR 4					2.7346			
HUI3 Pain 2YR 5					3.5874			

HUI3 Speech 2YR 2						4.0593		
HUI3 Hearing 2YR 2							3.2954	
HUI3 Hearing 2YR 3							3.6222	
HUI3 Vision 2YR 2								3.2813
HUI3 Vision 2YR 3								5.8796
HUI3 Vision 2	0.0640	0.1465	0.4326	0.1851	0.2271	0.0028	0.3944	
HUI3 Vision 3	0.3806	0.8775	1.1139	0.3662	0.8634	1.0215	0.7823	
HUI3 Hearing 2	-0.0925	0.2631	0.2904	0.0942	0.1155	0.4614		
HUI3 Hearing 3	0.3091	0.1911	0.4687	0.5768	0.5491	1.9380		
HUI3 Speech 2	0.0568	0.8741	1.9634	1.1619	0.6218			
HUI3 Pain 2	0.4368	0.6621	0.8342	0.2393				
HUI3 Pain 3	0.5771	1.1544	1.8215	0.4609				
HUI3 Pain 4	0.6875	1.5441	1.9544	0.6885				
HUI3 Pain 5	0.7713	2.4722	2.7766	0.9045				
HUI3 Cognition 2	1.3051	0.8188	0.0435					
HUI3 Cognition 3	0.4359	0.1217	0.3209					
HUI3 Cognition 4	1.3617	0.4902	0.6304					
HUI3 Cognition 5	1.4614	0.9406	0.3099					
HUI3 Dexterity 2	0.4444	1.3707						
HUI3 Mobility 2	0.0336							
HUI3 Mobility 3	-0.1006							
HUI3 Mobility 4	0.3423							
Osteoarthritis	-0.0119	0.2856	0.3273	0.0404	1.0004	-0.6262	0.2255	0.1271
Diabetes	0.1144	0.4601	0.3657	-0.0334	0.1905	0.1411	-0.2285	-0.0346
High blood pressure	0.0855	0.2347	-0.4145	0.0152	0.1375	0.0770	-0.0216	0.0548
CVD	0.1054	0.3363	0.0945	0.2683	0.3058	-0.1942	0.1044	0.2258
Stroke	0.0609	0.8996	0.8654	0.2688	0.4838	1.9335	0.1939	0.3437
Ulcer	0.0929	0.0827	-0.0232	0.1606	0.6438	0.6892	0.2083	0.1522
Dyspepsia	0.0372	0.0331	-0.0093	0.0642	0.2575	0.2757	0.0833	0.0609
Log10year-2000+1	0.0131	0.0178	-0.4691	0.4111	0.0674	0.7969	-0.1604	-0.0303

The coefficients were estimated from an ordinal logistic regression model with proportional odds. To avoid causal circularity, models are hierarchical in the reverse order as that presented in the table (vision, hearing, speech, pain, cognition, dexterity, mobility, emotion). For each domain, the model includes updated values of other domains that are lower in the hierarchy (already updated in earlier steps) as well as prior values of the specific domain being modeled in the current step. For example, emotion can be affected by current values of all other domains, whereas vision cannot be affected by current values of any other domain. 2YR=value 2 years back. CVD=cardiovascular disease; Numbers next to domain acronyms indicate domain levels. LOG10YEAR-2000+1 denotes the effect of calendar year (time trend). Reference levels were: age<30, male, post-secondary education, highest income (quartile 4), normal BMI, non-smoker, no disease. Levels of education were: less than secondary school

graduation=1, secondary school graduation=2; some post-secondary=3, post-secondary graduation=4 (reference). Income adequacy levels were: lowest=1, lower middle=2, upper middle=3, highest=4 (reference).

Table A1-18: Estimated coefficients in models for each HUI3 domain post-surgery.

Parameter	Emotion	Mobility	Dexterity	Cognition	Pain	Speech	Hearing	Vision
Intercept 5				-9.7476	-7.6135			
Intercept 4	-8.7582			-8.3029	-5.5282			
Intercept 3	-6.7960	-8.1428		-6.9831	-3.6067		-10.223	-10.735
Intercept 2	-5.1686	-7.1598	-6.7832	-6.6327	-2.4114	-11.791	-9.1157	-4.6599
Female	-0.0104	0.6029	-0.1435	-0.8290	-0.2350	0.5617	-0.4037	0.7724
Age	-0.0022	0.0045	0.0065	-0.0036	-0.0108	0.0526	-0.0069	0.0085
Vision after JRS	-0.7751	0.1158	-0.2983	0.7784	-0.0461	0.5384	1.7216	
Hearing after JRS	-0.2350	0.0944	-0.2248	0.1737	0.1155	1.0846		
Speech after JRS	1.4115	-0.0379	-2.0481	-0.1373	1.4256			
Pain after JRS	0.8240	1.2459	0.7773	0.7330				
Cognition after JRS	0.4978	-0.1830	0.5790					
Dexterity after JRS	0.4246	0.1733						
Mobility after JRS	0.1523							
Vision before JRS								2.2333
Hearing before JRS							2.3949	1.0078
Speech before JRS						1.7750	0.3585	0.4544
Pain before JRS					0.0670	-0.4567	0.0934	0.0521
Cognition before JRS				0.5861	-0.0083	-0.3161	-0.3701	-0.1678
Dexterity before JRS			2.9333	0.5287	0.2653	-0.7172	0.9480	-0.0200
Mobility before JRS		1.0131	0.1979	0.3433	0.4723	0.6053	-0.1839	-0.3382
Emotion before JRS	0.5694	-0.0235	0.1241	0.0364	0.3124	0.8460	0.1625	0.3143

The coefficients were estimated from an ordinal logistic regression model with proportional odds.

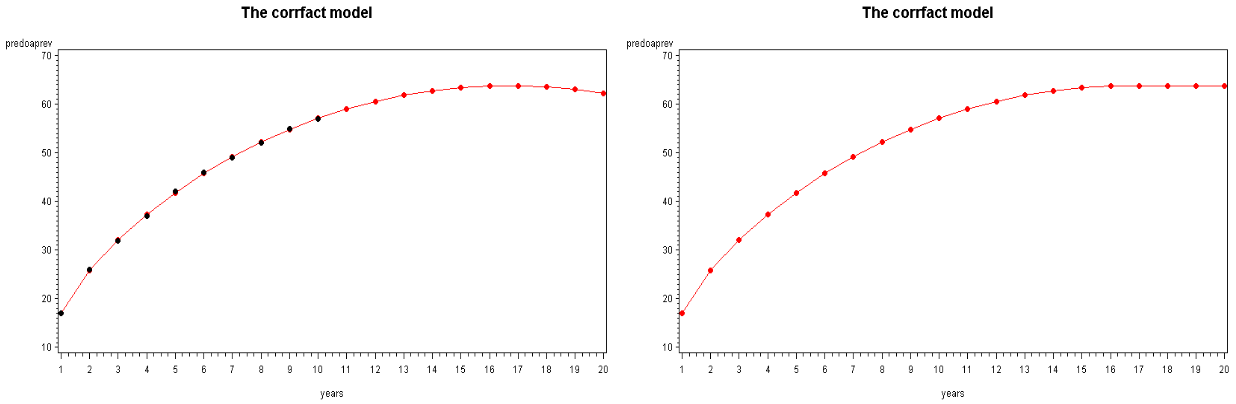

Figure A1-1: Modeling of the effect of run-in time on OA prevalence

Table A1-19: Comparison of population age/sex structure between MSM-OA and CCHS: Proportion (%) of males and females aged 70 years or older.

Sex	MSM-OA 2018	CCHS 2018
Male	11.9	13.2
Female	14.8	14.8
Total	13.4	14.0

MSM-OA = microsimulation model – osteoarthritis; CCHS = Canadian Population Health Survey

Appendix 2: Selection of scenarios

Table A2-1: QALYs for scenarios within the medical treatment strategy for specific drugs and drug combinations

Medication class	Target pain level	Odds multiplier	QALY gained/1000 population	
			Age 20-69	Age 70+
Base-case	n/a	1 (base-case)	0	0
Intervention targeting single pain levels				
Acetaminophen	2	Max	2.56	4.29
NSAIDs	2	Max	2.09	**-2.72**
NSAIDs	3	Max	7.44	6.99
NSAIDs	4	Max	14.56	30.03
Coxibs	2	Max	3.05	**-6.47**
Coxibs	3	Max	10.38	4.51
Coxibs	4	Max	19.87	39.89
Opioids	2	Max	**-0.38**	**-13.39**
Opioids	3	Max	7.88	1.84
Opioids	4	Max	20.00	44.67
Scenarios targeting multiple pain levels				
Acetaminophen	All	0.5	**-4.34**	**-20.71**
Acetaminophen	All	3	10.71	33.86
Acetaminophen	Optimal	3	10.61	35.34
NSAIDs	All	0.5	**-11.08**	**-13.09**
NSAIDs	All	3	17.15	13.21
NSAIDs	Optimal	3	19.28	42.67
NSAIDs	Optimal	Max	39.99	78.82
Coxibs	All	0.5	**-2.71**	**-2.80**
Coxibs	All	3	8.65	8.29
Coxibs	Optimal	3	8.97	11.35
Coxibs	Optimal	Max	54.88	99.65
Opioids	All	0.5	**-4.48**	**-10.51**
Opioids	All	3	11.67	24.67
Opioids	Optimal	3	12.28	26.83
Opioids	Optimal	Max	48.63	104.61
All	All	0.5	**-24.91**	**-51.63**
All	All	1.5	15.87	26.53
All	All	2	26.82	41.79
All	All	3	41.53	57.04
All	All	Max	68.23	**-200.16**
All	Optimal	2	28.17	63.38
All	Optimal	3	44.09	93.51
All	Optimal	Max	107.83	136.03
All	All	0	**-70.29**	**-194.85**

Max multiplier indicates that all people in the target group receive medication. Optimal intervention is defined as an increase in medication use in the pain-age combinations that benefit from the intervention, that is: for NSAIDs and Coxibs pain 2-5 in people age 20-69 and pain 3-5 in people aged 70+; for opioids, pain 3-5 in all age groups; for acetaminophen – all people with pain 2-5. QALYs gained depend on QALY gain/loss per person treated and number of people in the target group. Scenarios with negative gains (losses) in QALYs relative to base-case are shown in bold. The selected scenarios are shaded.

Table A2-2: QALYs and effectiveness ratios for scenarios within the surgical treatment strategy.

Target sub-population		Rate multiplier	QALY gained/1000 population	Effectiveness ratio
Age	Pain level			
n/a	n/a	1 (base-case)	0	0
<50	2	0.5	**-0.02**	0.03
50-69	4	0.5	**-5.49**	0.27
70+	4	0.5	**-15.65**	0.30
<50	2	2	0.20	0.15
<50	3	2	0.24	0.09
<50	4	2	0.50	0.23
<50	5	2	0.47	0.31
50-69	2	2	1.98	0.11
50-69	3	2	6.78	0.20
50-69	4	2	8.16	0.30
50-69	5	2	7.51	0.40
70+	2	2	6.22	0.20
70+	3	2	18.14	0.26
70+	4	2	20.38	0.31
70+	5	2	21.05	0.35
<50	2	20	1.20	0.08
<50	3	20	3.24	0.11
<50	4	20	4.45	0.22
<50	5	20	4.08	0.37
50-69	2	20	18.69	0.12
50-69	3	20	43.20	0.18
50-69	4	20	48.69	0.28
50-69	5	20	41.64	0.44
70+	2	20	45.49	0.21
70+	3	20	104.93	0.26
70+	4	20	113.56	0.32
70+	5	20	106.05	0.39
All	2-5	2	83.01	0.25
All	2-5	3	129.29	0.24
All	2-5	20	252.56	0.17

Effectiveness ratio can be interpreted as QALYs gained in the population per 1 extra JRS performed (or QALYs lost per 1 JRS eliminated). Scenarios with negative gains (losses) in QALYs relative to base-case are shown in bold. The selected scenarios are shaded.

Appendix 3: Sensitivity analysis

We estimated the impact of uncertainty in 17 key model parameters on the results in a one-way sensitivity analysis. The parameters, their confidence intervals, and source of data are listed in Table A3-1.

Table A3-1: Model parameters selected for sensitivity analysis.

Parameter	Mean	Lower limit	Upper limit	Source
Obesity RR on OA male	1.69	1.06	2.69	NPHS analysis
Obesity RR on OA female	2.03	1.49	2.76	NPHS analysis
NSAIDs pain % reduction	38.2	36.4	40.0	Literature [40]
Coxibs pain % reduction	38.2	36.4	40.0	Literature [40]
Acetaminophen pain % reduction	28.2	26.5	29.9	Literature [40]
Opioids pain % reduction	40.2	38.4	42.0	Literature [40]
NSAIDs CVD RR	1.3	1.1	1.5	Literature [26,30]
NSAIDs GI excess incidence	7.4	5.4	9.4	Literature [30]
OA pain effect (intercept 2)	-3.6410	-3.8243	-3.4577	CCHS analysis
OA pain effect (intercept 3)	-4.1129	-4.3001	-3.9258	CCHS analysis
OA pain effect (intercept 4)	-5.0737	-5.2543	-4.8931	CCHS analysis
OA pain effect (intercept 5)	-6.3890	-6.5813	-6.1968	CCHS analysis
OA pain effect (coefficient)	1.0004	0.8997	1.1011	CCHS analysis
Post-surgery OA pain intercept 2	-7.6135	-10.1303	-5.0967	VGH Study analysis
Post-surgery OA pain intercept 3	-5.5282	-7.7533	-3.3030	VGH Study analysis
Post-surgery OA pain intercept 4	-3.6067	-5.7366	-1.4768	VGH Study analysis
Post-surgery OA pain intercept 5	-2.4114	-4.5156	-0.3072	VGH Study analysis

NPHS = National Population Health Survey [21]; CCHS = Canadian Community Health Survey [19]; VGH Study = Vancouver General Hospital Study [37]; RR = relative risk

For each parameter, we ran the model for the base-case and three intervention scenarios (one per strategy) assuming three values for the parameter, the mean, lower limit, and upper limit of the 95% confidence interval. We selected the Medication x2, Surgery x2 and BMI-0.3 scenarios for this analysis. For each value of each parameter, we computed lifetime QALYs per person for the base-case scenario and each intervention scenario. We specified the sample size as 1 million simulated individuals for the duration of the simulation, which resulted in a population of about 360,000 in 2020. It may be noted that the sample size for the sensitivity analysis was 1/10 of the sample size for the main analysis (this was necessary for feasibility). As a result, standard error due to random variation in the model was around 0.018-0.019, which is significantly larger than uncertainty associated with some of the model parameters (e.g., reduction in pain due to medication). Therefore, the impact of these parameters should be interpreted with caution.

The results are presented numerically (actual QALYs) in Tables A3-2 – A3-5 and graphically in Figures A3-1 – A3-3 (tornado plots). The plots show the differences in QALYs between base-case and intervention scenarios for the mean, lower, and upper limit of the confidence interval for each parameter.

Table A3-2. Life-time QALYs per person in the Canadian population age 20+ for the mean, lower limit, and upper limit of the estimated 95% confidence interval for each parameter: Base-case

Parameter	Mean	Lower limit	Upper limit
Obesity RR on OA male	29.48794	29.45884	29.51122
Obesity RR on OA female	29.48794	29.46615	29.50779
NSAIDs pain % reduction	29.48794	29.48135	29.49506
Coxibs pain % reduction	29.48794	29.48709	29.4897
Acetaminophen pain % reduction	29.48794	29.48417	29.49331
Opioids pain % reduction	29.48794	29.48611	29.48981
NSAIDs CVD RR	29.48794	29.45187	29.52577
NSAIDs GI excess incidence	29.48794	29.4849	29.49011
OA pain effect (intercept 2)	29.48794	29.32438	29.63867
OA pain effect (intercept 3)	29.48794	29.40622	29.5637
OA pain effect (intercept 4)	29.48794	29.41423	29.55521
OA pain effect (intercept 5)	29.48794	29.44037	29.52954
OA pain effect (coefficient)	29.48794	29.431	29.54539
Post-surgery OA pain intercept 2	29.48794	29.39048	29.55509
Post-surgery OA pain intercept 3	29.48794	29.36009	29.52483
Post-surgery OA pain intercept 4	29.48794	29.41968	29.50072
Post-surgery OA pain intercept 5	29.48794	29.4641	29.48946
Obesity RR on OA male & female	29.48794	29.43706	29.53106

Table A3-3: Life-time QALYs per person in the Canadian population age 20+ for the mean, lower limit, and upper limit of the estimated 95% confidence interval for each parameter: Medication x2 scenario

Parameter	Mean	Lower limit	Upper limit
Obesity RR on OA male	29.54821	29.52261	29.56845
Obesity RR on OA female	29.54821	29.52994	29.56539
NSAIDs pain % reduction	29.54821	29.54327	29.55701
Coxibs pain % reduction	29.54821	29.54761	29.54951
Acetaminophen pain % reduction	29.54821	29.54367	29.55478
Opioids pain % reduction	29.54821	29.54624	29.55088
NSAIDs CVD RR	29.54821	29.5095	29.58927
NSAIDs GI excess incidence	29.54821	29.54443	29.55099
OA pain effect (intercept 2)	29.54821	29.39023	29.70037
OA pain effect (intercept 3)	29.54821	29.46713	29.62397
OA pain effect (intercept 4)	29.54821	29.47697	29.61468
OA pain effect (intercept 5)	29.54821	29.50284	29.58838
OA pain effect (coefficient)	29.54821	29.49782	29.60148
Post-surgery OA pain intercept 2	29.54821	29.47196	29.60397
Post-surgery OA pain intercept 3	29.54821	29.44272	29.58182
Post-surgery OA pain intercept 4	29.54821	29.49043	29.55892
Post-surgery OA pain intercept 5	29.54821	29.52785	29.54964
Obesity RR on OA male & female	29.54821	29.50435	29.58562

Table A3-4: Life-time QALYs per person in the Canadian population age 20+ for the mean, lower limit, and upper limit of the estimated 95% confidence interval for each parameter: Surgery x2 scenario

Parameter	Mean	Lower limit	Upper limit
Obesity RR on OA male	29.57192	29.54735	29.59088
Obesity RR on OA female	29.57192	29.55444	29.58745
NSAIDs pain % reduction	29.57192	29.56649	29.57888
Coxibs pain % reduction	29.57192	29.57119	29.57364
Acetaminophen pain % reduction	29.57192	29.56821	29.57722
Opioids pain % reduction	29.57192	29.57026	29.57374
NSAIDs CVD RR	29.57192	29.53622	29.60899
NSAIDs GI excess incidence	29.57192	29.56885	29.57413
OA pain effect (intercept 2)	29.57192	29.41119	29.7188
OA pain effect (intercept 3)	29.57192	29.49211	29.64509
OA pain effect (intercept 4)	29.57192	29.49982	29.63669
OA pain effect (intercept 5)	29.57192	29.52732	29.61174
OA pain effect (coefficient)	29.57192	29.52237	29.62466
Post-surgery OA pain intercept 2	29.57192	29.43937	29.65553
Post-surgery OA pain intercept 3	29.57192	29.40265	29.61637
Post-surgery OA pain intercept 4	29.57192	29.48517	29.58779
Post-surgery OA pain intercept 5	29.57192	29.54078	29.57395
Obesity RR on OA male & female	29.57192	29.52988	29.60642

Table A3-5: Life-time QALYs per person in the Canadian population age 20+ for the mean, lower limit, and upper limit of the estimated 95% confidence interval for each parameter: BMI-0.3 scenario

Parameter	Mean	Lower limit	Upper limit
Obesity RR on OA male	29.61932	29.60566	29.62859
Obesity RR on OA female	29.61932	29.60795	29.62941
NSAIDs pain % reduction	29.61932	29.61323	29.62532
Coxibs pain % reduction	29.61932	29.61801	29.62048
Acetaminophen pain % reduction	29.61932	29.61521	29.62434
Opioids pain % reduction	29.61932	29.61751	29.62117
NSAIDs CVD RR	29.61932	29.58434	29.65628
NSAIDs GI excess incidence	29.61932	29.61606	29.62143
OA pain effect (intercept 2)	29.61932	29.45983	29.76647
OA pain effect (intercept 3)	29.61932	29.53938	29.69182
OA pain effect (intercept 4)	29.61932	29.54647	29.68369
OA pain effect (intercept 5)	29.61932	29.57466	29.65783
OA pain effect (coefficient)	29.61932	29.56791	29.6702
Post-surgery OA pain intercept 2	29.61932	29.53671	29.67801
Post-surgery OA pain intercept 3	29.61932	29.51034	29.65134
Post-surgery OA pain intercept 4	29.61932	29.56087	29.63032
Post-surgery OA pain intercept 5	29.61932	29.59867	29.62053
Obesity RR on OA male & female	29.61932	29.59429	29.63868

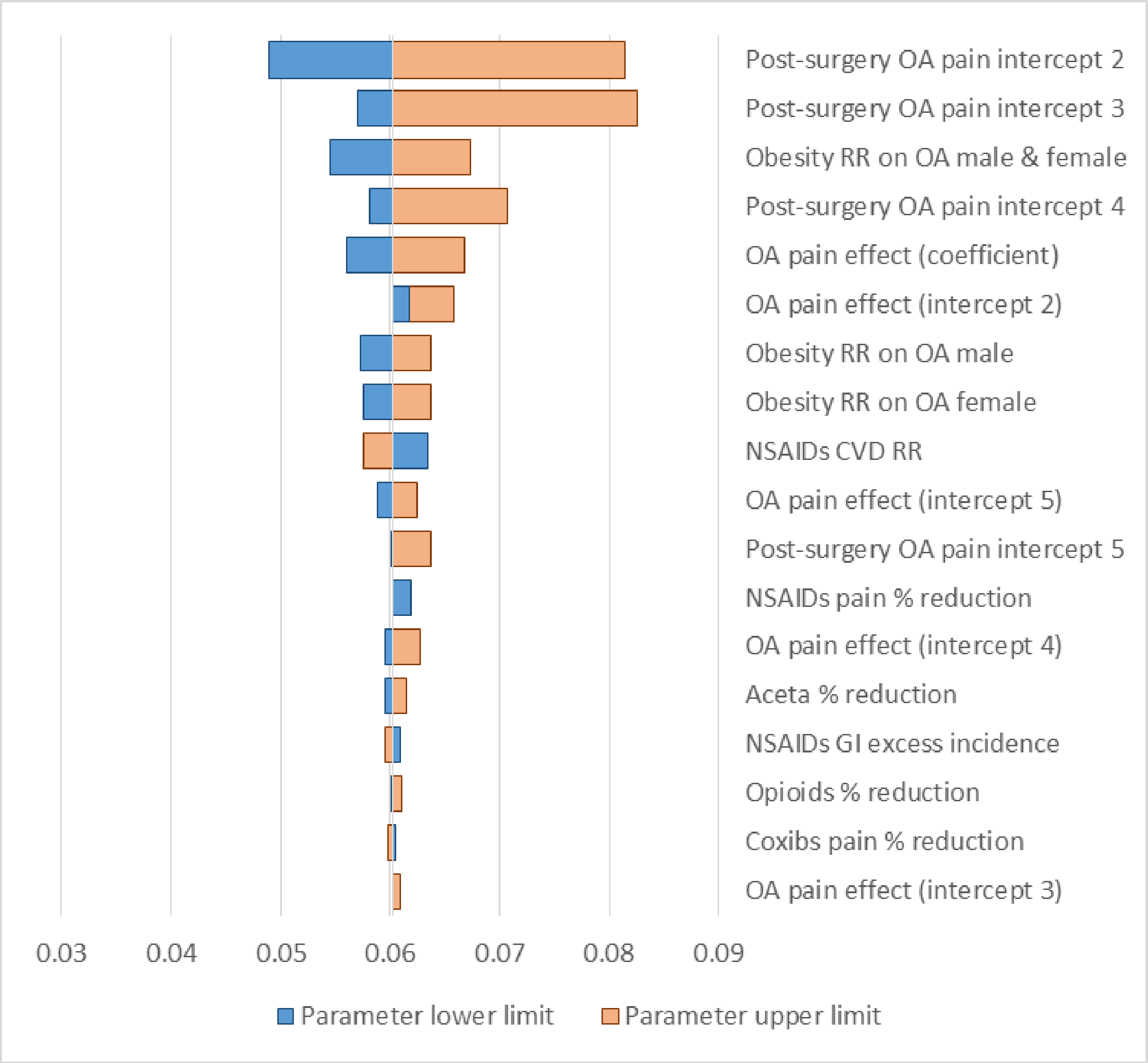

Figure A3-1: Tornado plot of the impact of uncertainty in 17 parameters on the difference in lifetime QALYs per person between base-case and Medication x2 intervention scenario.

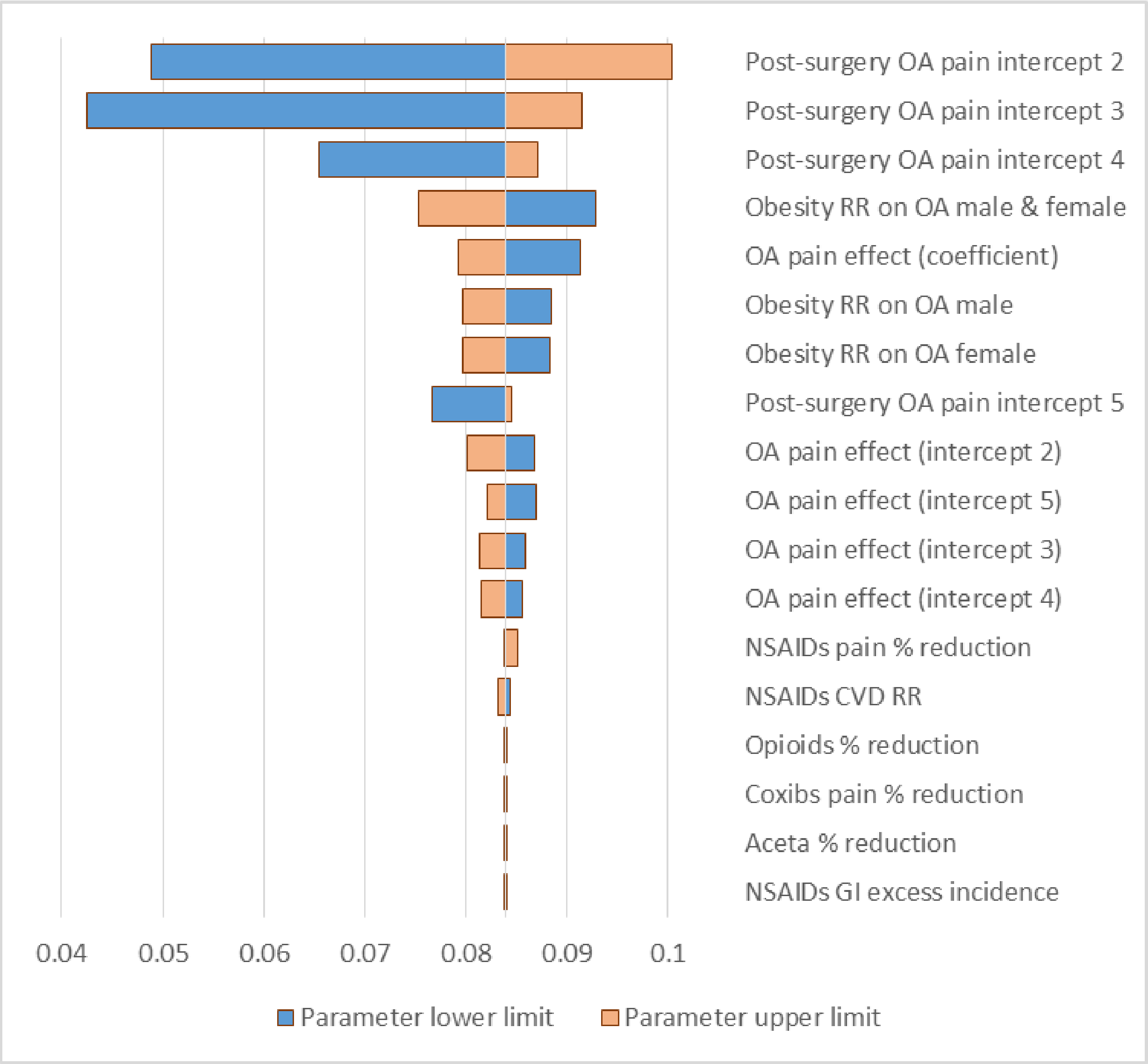

Figure A3-2: Tornado plot of the impact of uncertainty in 17 parameters on the difference in lifetime QALYs per person between base-case and Surgery x2 intervention scenario.

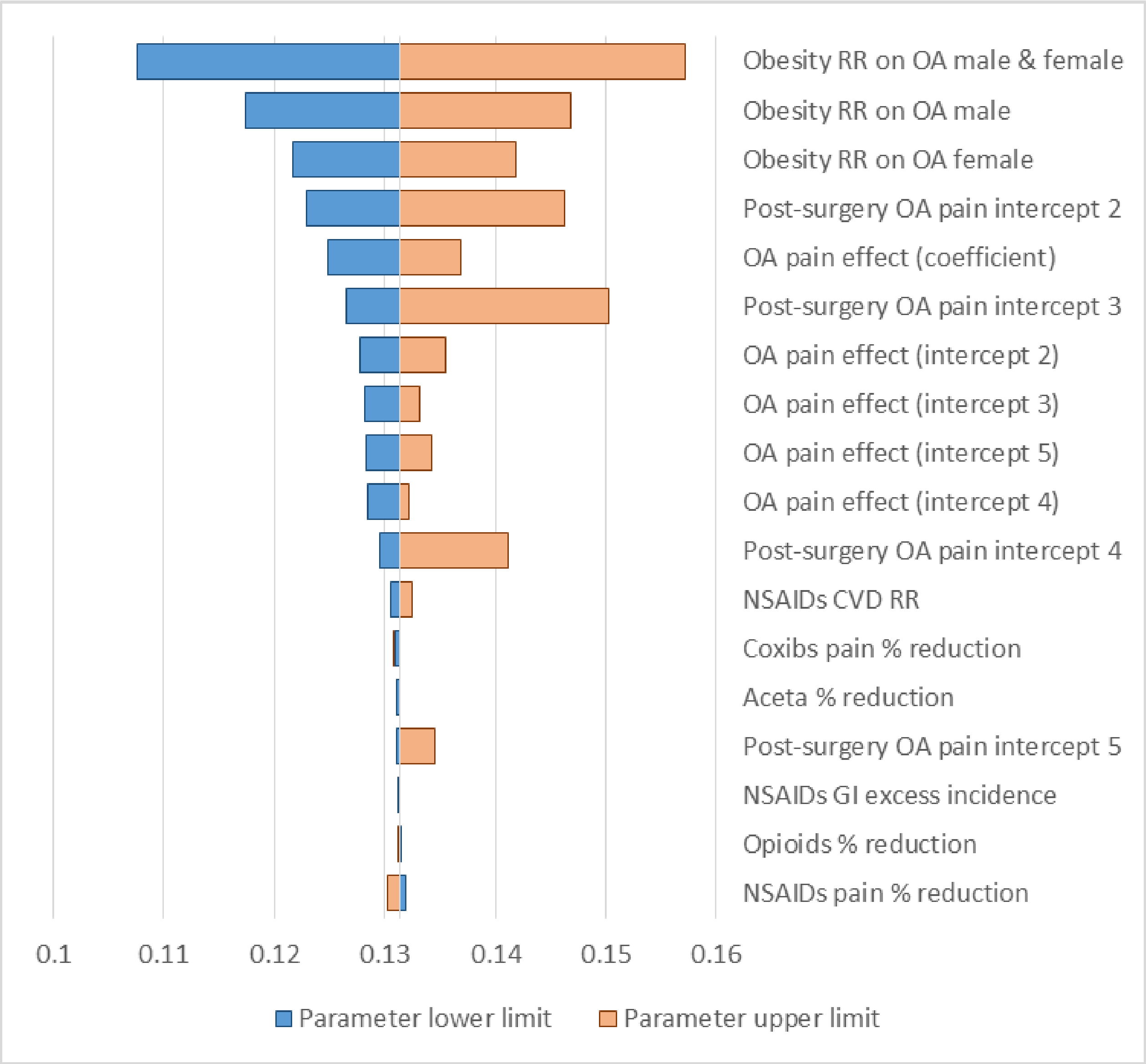

Figure A3-3: Tornado plot of the impact of uncertainty in 17 parameters on the difference in lifetime QALYs per person between base-case and BMI-0.3 intervention scenario.
